# Interpretable multi-omics integration across mixed-order tensors with MANTRA

**DOI:** 10.1038/s44320-026-00223-8

**Published:** 2026-06-18

**Authors:** Kevin De Azevedo, Yusuf Berk Oruc, Florian Buettner

**Affiliations:** 1 Institute of Informatics, Goethe University Frankfurt, Frankfurt am Main, Germany; 2 German Cancer Consortium (DKTK), Partner Site Frankfurt/Mainz, a partnership between DKFZ and UCT Frankfurt-Marburg, Frankfurt am Main, Germany; 3 German Cancer Research Center (DKFZ), Heidelberg, Germany; 4 Department of Medicine, Goethe University Frankfurt, Frankfurt am Main, Germany; 5 Frankfurt Cancer Institute (FCI), Frankfurt am Main, Germany

**Keywords:** Computational Biology

## Abstract

The integration of multi-modal molecular data is crucial for understanding complex diseases, but existing methods struggle with modern experimental designs that generate datasets with mixed-order tensors—for example, a third-order drug–response tensor alongside a second-order transcriptomics matrix. Here, we present MANTRA (Multi-view ANalysis with Tensor and matRix Alignment), a probabilistic framework that integrates collections of tensors of different orders, combining the strengths of group factor analysis and tensor decomposition. MANTRA learns interpretable latent factors and naturally handles missing data through a Bayesian approach with structured sparsity priors. On a Chronic Lymphocytic Leukemia (CLL) dataset, the joint analysis of a third-order drug–response tensor and a second-order RNA-seq matrix with MANTRA revealed clinically relevant patient subgroups that were missed by single-view or matrix-based analyses. In a single-cell multi-omics study of Acute Lymphoblastic Leukemia (ALL), MANTRA identified a novel patient subgroup defined by a distinct molecular program in plasmacytoid dendritic cells (pDCs), linking disease heterogeneity to a specific cell type. By explicitly modeling higher- order data structures, MANTRA provides an interpretable tool to uncover hidden biological variation from complex experimental data.

## Synopsis

MANTRA is a Bayesian framework for integrating matrices and higher-order tensors in multi-omics studies, enabling interpretable discovery of clinically relevant patient structure and cell-type-specific disease programs.

- MANTRA jointly models mixed-order datasets, preserving higher-dimensional structure that is lost in standard matrix-based integration.
- MANTRA learns sparse, interpretable latent factors while naturally accommodating missing data through Bayesian inference.
- MANTRA outperforms matrix-based baselines in identifying clinically meaningful subgroups in Chronic Lymphocytic Leukemia (CLL) data.
- MANTRA uncovers a plasmacytoid dendritic cell (pDC)-associated disease program in pediatric B-cell acute lymphoblastic leukemia (B-ALL) that is not detected by conventional 2D integration methods (e.g., MOFA + , MOFA-FLEX) or specialized 3D approaches such as scITD.

## Abstract (web summary)

MANTRA is a Bayesian framework for integrating matrices and higher-order tensors in multi-omics studies, enabling interpretable discovery of clinically relevant patient structure and cell-type-specific disease programs.

## Graphical abstract

**Figure:**
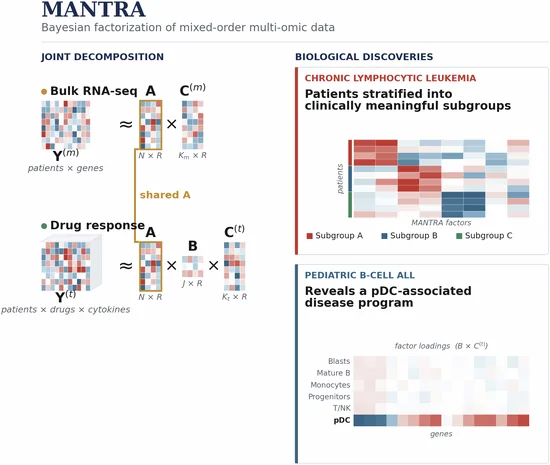

**Figure:**
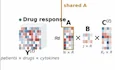

## Introduction

The increasing availability of high-dimensional, multi-modal molecular data offers unprecedented opportunities for advancing personalized medicine. Comprehensive characterization of patients across multiple molecular layers has already led to the discovery of novel prognostic subgroups and mechanisms of therapeutic resistance (Jayavelu et al, 2022; Li et al, 2023; Mani et al, 2022; Qoku et al, 2023). These multi-modal approaches are particularly promising for understanding diseases with high inter-patient heterogeneity, where the underlying sources of variation are largely unknown. In such settings, unsupervised modeling approaches can reveal and characterize the main axes of variation in an interpretable, data-driven manner.

Current computational strategies for unsupervised multi-omics integration predominantly fall into two categories. The first includes matrix decomposition methods, such as Principal Component Analysis (PCA) and its multi-view generalization, Multi-Omics Factor Analysis (MOFA), which aim to find a shared low-dimensional representation of the data (Argelaguet et al, 2018). The second category involves deep learning approaches, such as variational autoencoders, which can capture non-linear relationships (Gayoso et al, 2021). However, deep learning models often produce embeddings with limited biological interpretability. Furthermore, both paradigms struggle to handle missing data in a principled way - a frequent challenge in clinical studies where not all omics layers can be profiled for every patient (Argelaguet et al, 2018; Qoku and Buettner, 2023). A more fundamental limitation is that these methods treat features within each modality as an unstructured set, ignoring potential relationships between them.

In many experimental designs, however, data have a higher-order structure that is best represented as a tensor. For example, single-cell profiling of multiple tissues from a patient cohort naturally yields a third-order tensor (patients × genes × tissues) (Mitchel et al, 2025). Similarly, high-throughput drug screens can produce a tensor of patients × drugs × conditions (Bruch et al, 2022). Recently, tensor decomposition has been adapted to jointly model expression variation across multiple cell types, successfully identifying coordinated gene program patterns of variation across multiple cell types with the scITD tool (Mitchel et al, 2025). This method, however, is constrained to analyzing a single data tensor at a time. This limitation is significant, as many studies generate complementary datasets that do not share the same structure. For instance, a study might generate a third-order drug response tensor alongside a standard second-order RNA-seq matrix (patients × genes). Similarly, in the case of modeling inter-patient variation across tissues, patients are often not only profiled in terms of gene expression alone (patients×genes×tissues), but also via additional modalities such as ATAC-seq to measure chromatin accessibility (Chen et al, 2022). Finally, scITD requires each cell type to be observed for every donor, without allowing for missing values. There is currently no framework that can jointly model such a collection of tensors of varying orders.

To address this gap, we present MANTRA (Multi-view ANalysis with Tensor and matRix Alignment), a probabilistic framework that integrates collections of tensors of different orders. MANTRA can be viewed as a generalization of group factor analysis to tensors, or conversely, as a generalization of tensor decomposition to multiple views. We propose a Bayesian model that employs structured sparsity priors, based on Automatic Relevance Determination (ARD) (MacKay, 1994), to learn interpretable latent factors. This framework provides several key advantages: (1) flexible integration of tensors of different orders, (2) inference of interpretable embeddings via structured sparsity, (3) principled handling of missing values, and (4) fast and scalable model training using variational inference.

We first validate MANTRA on simulated data, demonstrating its ability to accurately model collections of tensors and recover sparse, interpretable solutions. We then showcase its broad applicability on two complex, real-world datasets. First, we analyze drug–response data from 192 Chronic Lymphocytic Leukemia (CLL) patients, integrating a third-order drug viability tensor with a second-order RNA-seq matrix (Bruch et al, 2022). MANTRA learns patient embeddings that are strongly associated with key clinical covariates, a signal that is not completely captured when analyzing the views separately. Second, we apply MANTRA to a single-cell multi-omics study of KMT2A-rearranged leukemia, integrating third-order tensors from scRNA-seq and scATAC-seq data (Chen et al, 2022). Our analysis reveals interpretable multi-modal patterns capturing structured variation across patients, cell types, and modalities that were associated with disease state and could be explained via cell type- and modality-specific pathway activities.

## Results

**Figure 1:**
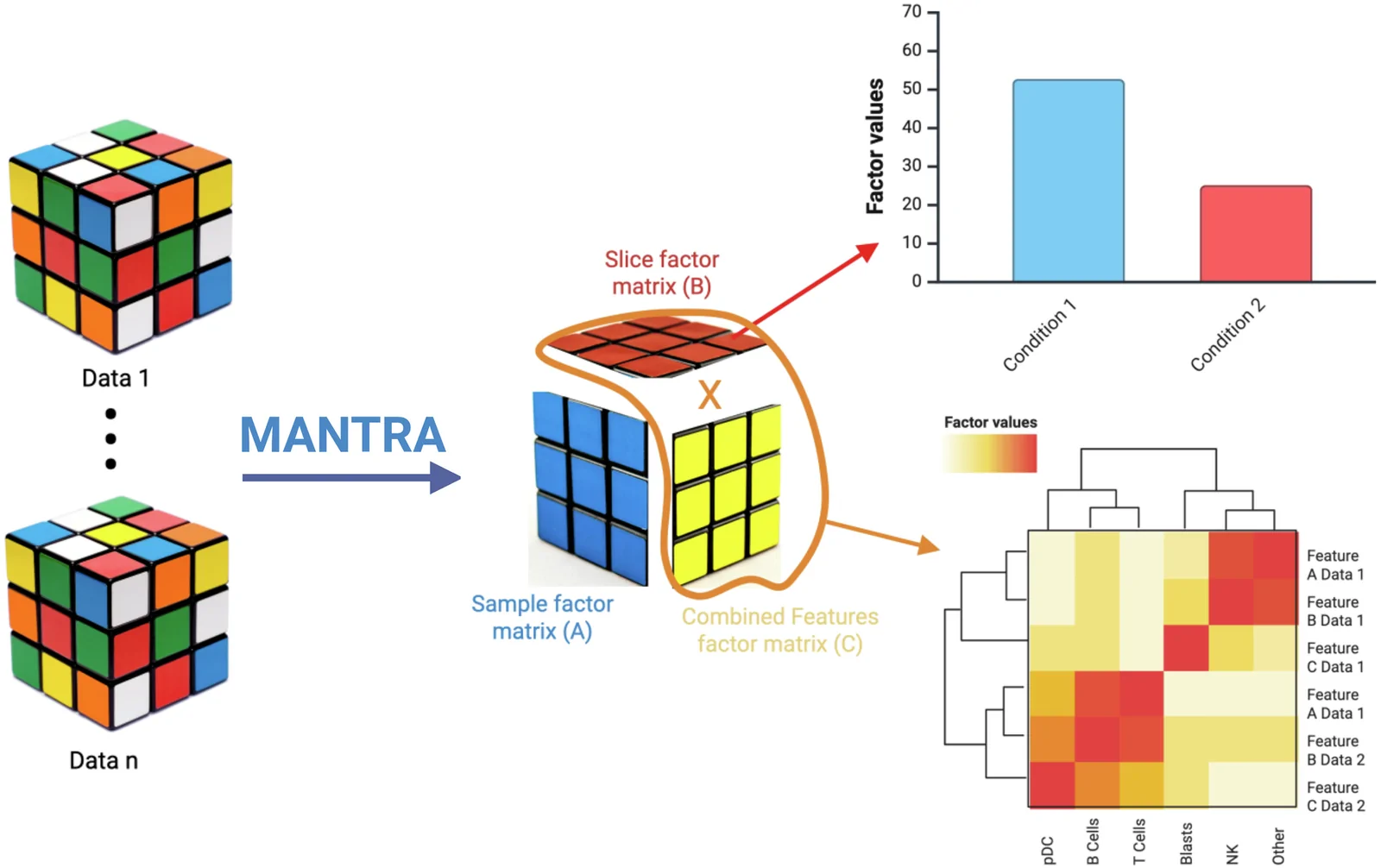
MANTRA model overview. MANTRA decomposes several datasets in the form of tensors into common latent factor matrices (or embeddings), one for each dimension in the data. Individual factor embeddings can be investigated for differences between variables of interest. A combination of factor embeddings into factor loadings by matrix multiplication can reveal unknown interactions in the latent space between two aspects of the data, such as the cell types of the samples and the features in the different omics layers.

We developed MANTRA, a Bayesian multi-view tensor decomposition framework. At a high level, MANTRA takes a collection of data tensors, which can have different dimensions (e.g., a 3D drug–response tensor and a 2D gene expression matrix), and jointly decomposes them into a set of shared, low-dimensional factors. These factors represent the principal axes of variation across the different data types. For example, one set of factors represents shared patterns across patients, while other factors capture patterns across features like genes or drugs. By using a Bayesian approach with structured sparsity, MANTRA learns interpretable factors and can naturally handle missing data. A detailed model description is provided in the Methods section. For clarity, we also refer to the latent factors as “embeddings” or “factors”, and the product of two embedding matrices as “loadings” (Fig. 1).

### MANTRA accurately reconstructs data and yields sparse, interpretable solutions

To validate MANTRA, we first assessed its performance on simulated data. We aimed to confirm that MANTRA can accurately reconstruct data from a single tensor, comparing it to a state-of-the-art implementation of PARAFAC from the Tensorly package (Kossaifi et al, 2019). We generated synthetic 3D tensor datasets and compared the relative root-mean-square error (RMSE) between the original and reconstructed data for both methods.

**Figure 2:**
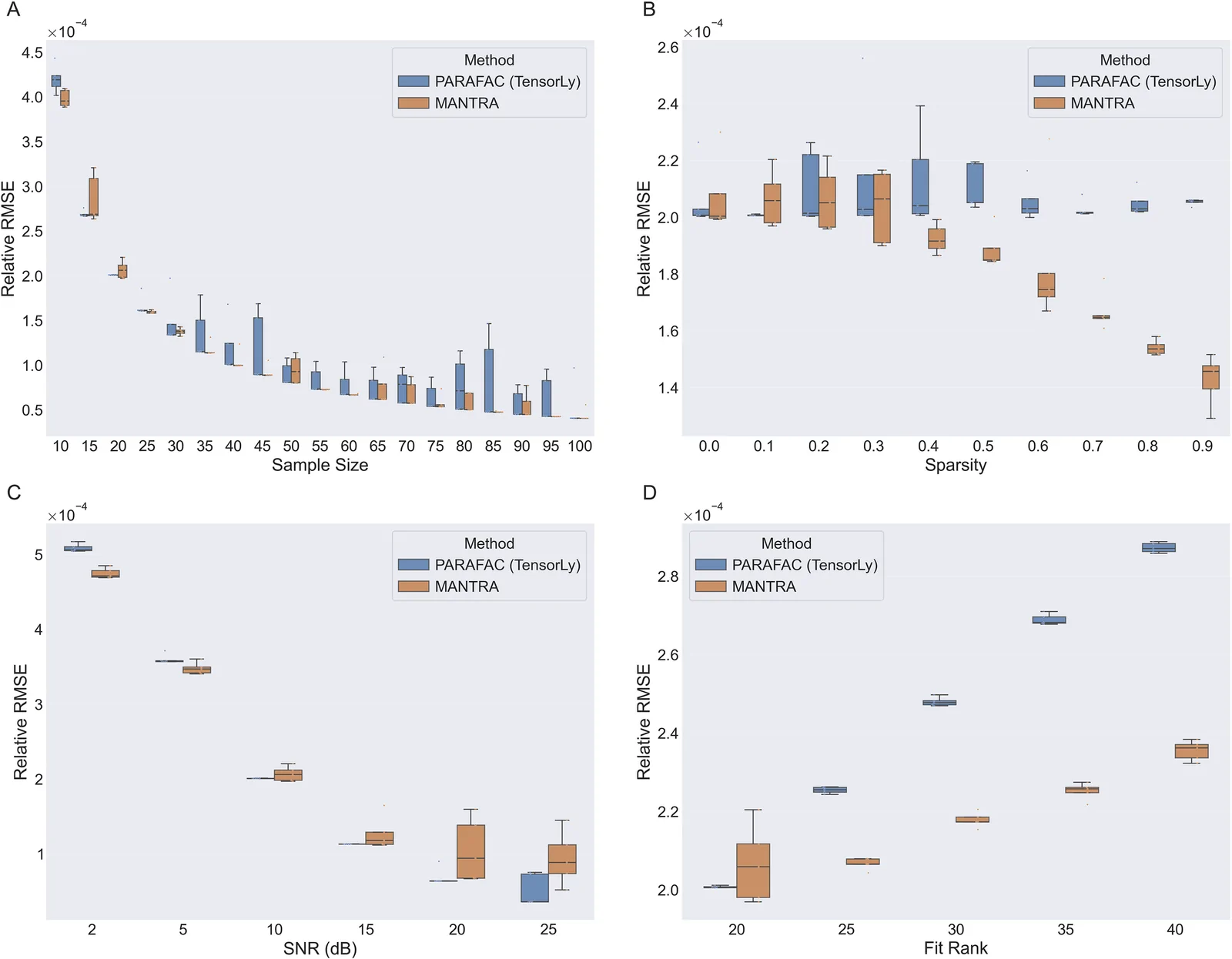
MANTRA achieves robust reconstruction accuracy across diverse data regimes compared to PARAFAC. (**A**) Relative RMSE of Tensorly’s PARAFAC method and MANTRA across varying sample sizes. (**B**) Relative RMSE across different sparsity levels. (**C**) Relative RMSE across signal-to-noise ratios (dB, decibels). (**D**) Relative RMSE across fitted ranks. RMSE was calculated relative to the norm of the simulated ground-truth tensor to facilitate interpretation. In the box plots (e.g., **A**), the box denotes the interquartile range (IQR), defined as the range between the 25th (Q1) and 75th (Q3) percentiles. Whiskers extend to the most extreme data points within 1.5× the IQR. The central line represents the median, and individual points denote individual observations.

To characterize MANTRA’s behavior across typical data environments and modeling choices, we established a default setting (Methods) and systematically varied one factor at a time, specifically investigating: (i) number of samples (Fig. 2A), (ii) sparsity in the data (Fig. 2B), (iii) signal-to-noise ratio (SNR) (Fig. 2C), (iv) number of features (Appendix Fig. S1A), (v) the number of latent factors (Appendix Fig. S1B) and (vi) random noise (Appendix Fig. S1C). In addition, we mimicked real-world applications where it is unclear what the true rank of the data is and assessed how robust MANTRA and PARAFAC are against mis-specified ranks (Fig. 2D). These results all together revealed that MANTRA has comparable performance to PARAFAC in the high SNR/high sample ratio and a substantially improved reconstruction error in the noisy data regime (moderate SNR, low sample size, moderate random noise and sparsity), mimicking real-world datasets with moderate-to-low SNR and moderate-to-high SNR (Fig. 2). In addition, due to its Bayesian formulation with sparsity priors, MANTRA was substantially more robust against rank mis-specification than PARAFAC as the structured sparsity prior encourages the model to switch off un-needed factors (i.e., drive the factor scores to 0). A complementary multi-view simulation benchmark in which the underlying ranks differed between views confirmed this robustness (Appendix Fig. S2A,B).

Next, we performed a time complexity analysis to evaluate MANTRA’s scalability across the parameters most frequently varied in practical applications: the number of samples, features, and ranks. MANTRA is computationally efficient both in terms of per-epoch execution time (Appendix Fig. S3A–C) and wall time per epoch (Appendix Fig. S3D–F). Finally, we conducted a hyperparameter sensitivity analysis (“Methods”) for ${\alpha }_{\sigma }$ and ${\beta }_{\sigma }$, which control the shape and scale of the inverse-Gamma prior over feature and view-specific noise variances, across varying data conditions. MANTRA is able to recover latent factors and minimize reconstruction error across a broad range of hyperparameter values, under both moderate-sparsity (Appendix Fig. S4, left) and high-sparsity (Appendix Fig. S4, right) regimes. This confirms that the choice of α=1 and β=0.5 provides a reliable prior for the inverse-Gamma distribution (Methods), ensuring the model’s robustness with respect to the underlying data sparsity.

In addition to resulting in improved robustness in the noisy real-world data regime, MANTRA’s structured sparsity aids in the interpretation of the learned factors by highlighting the most relevant features. Finally, MANTRA is designed to handle missing values in a principled manner and to integrate multiple tensors, capabilities that are neither supported by standard PARAFAC implementations nor by specialized tools with a focus on molecular data such as scITD (Mitchel et al, 2025).

### MANTRA leverages multi-view integration to uncover clinically relevant patient subgroups in CLL

Having validated its core performance, we next sought to demonstrate MANTRA’s ability to gain new biological insights by jointly modeling tensors of different orders. We analyzed a viability screening dataset from Bruch et al (Bruch et al, 2022), who investigated the influence of 17 microenvironmental stimuli on 12 drugs in 192 Chronic Lymphocytic Leukemia (CLL) patient samples. This yielded a third-order drug viability tensor (patients × drugs × cytokines) and a second-order RNA-seq matrix (patients × genes).

**Figure 3:**
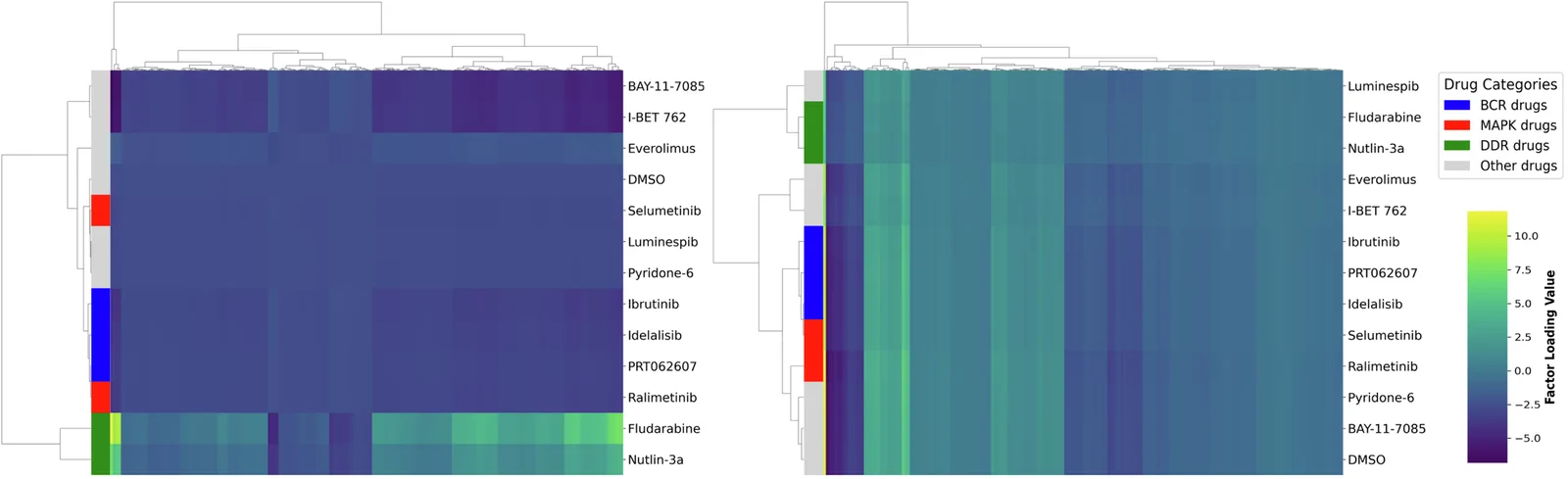
Multi-view integration refines patient–drug factor loadings and enhances biological separation. Heatmaps of factor loadings for patients relative to drugs in a single-view MANTRA model trained on the drug viability tensor only (left) and a multi-view MANTRA model trained on both the drug viability tensor and the RNA-seq matrix (right). Factor 11 (single-view model) and Factor 6 (multi-view model) were the most discriminative with respect to drug category in their respective models. Integration of RNA-seq data further improved the learned representations, enhancing separation between drug groups. The *x* axis represents patients, and the *y* axis represents drugs.

**Figure 4:**
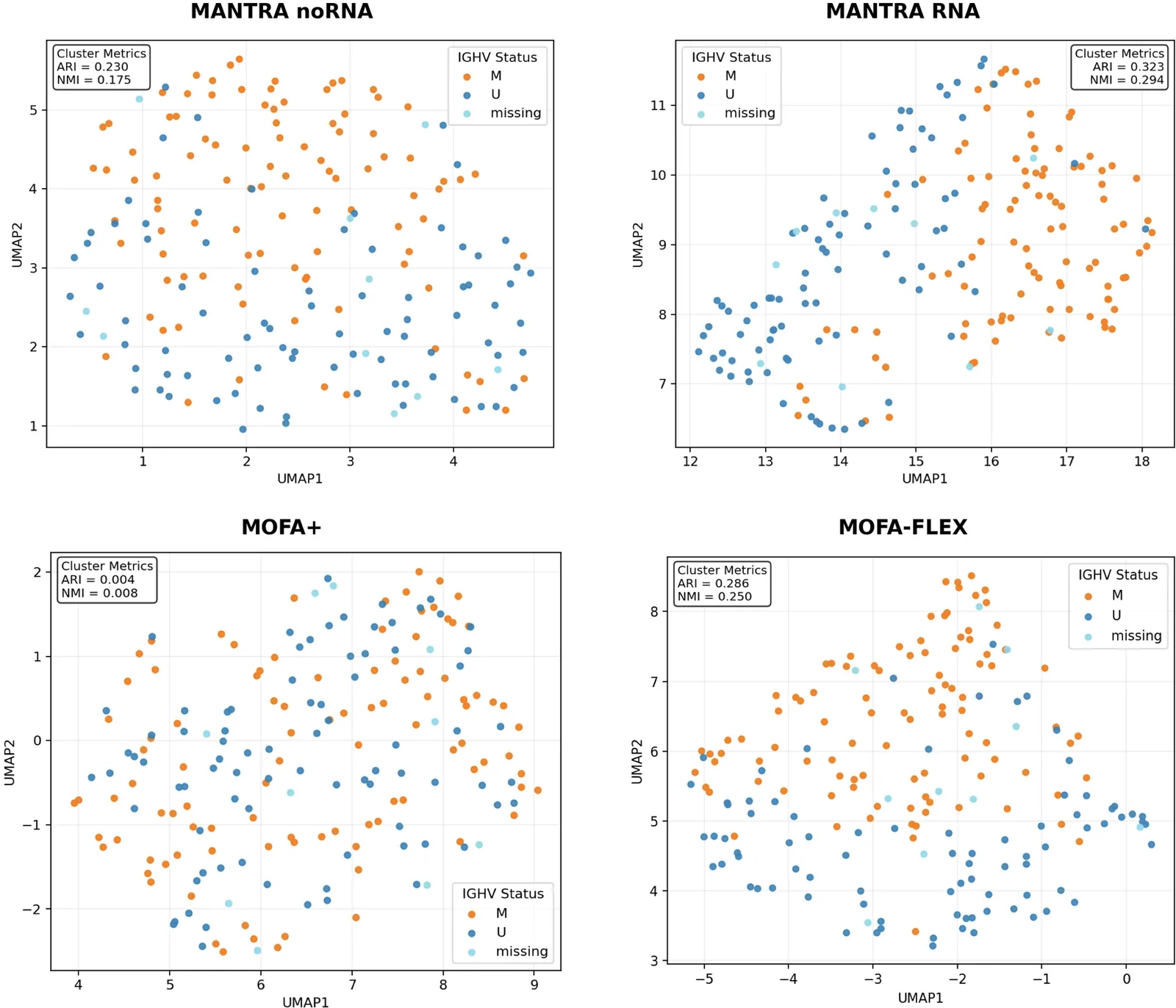
Multi-view MANTRA improves the structure of patient embeddings compared to existing methods. UMAP of patient embeddings learned by MANTRA (single-view, top left), MANTRA (multi-view, top right), MOFA + (bottom left), and MOFA-FLEX (bottom right). Both MOFA+ and MOFA-FLEX were trained based on all slices of the drug viability tensor as well as the RNA-seq view. Adjusted Rand Index (ARI) and Normalized Mutual Information (NMI) with respect to Leiden clusters were computed to quantify how well individual methods could separate patients grouped by IGHV status.

We first ran MANTRA using only the drug response tensor to establish a baseline. While the patient–drug loadings captured mode of action for BCR and DDR drugs (Fig. 3, left), the patient embeddings learned from this single view only showed a partial association with IGHV status (Normalized Mutual Information (NMI) 0.174; Fig. 4, top left). IGHV mutation status is one of the most important prognostic markers in CLL: patients with unmutated IGHV (U-CLL) typically experience more aggressive disease progression and shorter time to first treatment compared to those with mutated IGHV (M-CLL) (Hamblin et al, 1999).

We then jointly modeled the drug response tensor and the RNA-seq matrix and found that the learned drug loading (Fig. 3, right) as well as the patient embeddings (Fig. 4) were substantially refined. In this multi-view setting, MANTRA uncovered a clearer separation of patients based on their IGHV mutation status (Fig. 4, top right). In addition, the model also successfully recovered known biological relationships in the drug space, with drugs targeting the B-cell receptor (BCR), MAPK, and DNA damage response (DDR) pathways clustering together in the loadings (Fig. 3, right). To systematically assess the consistency between the patient embeddings learned from the single-view and multi-view settings, respectively, we computed pair-wise correlation coefficients between factors of both models. This revealed that indeed Factor 11 in the single-view model (trained on the drug viability tensor only) was strongly correlated to Factor 19 (Appendix Fig. S5) in the multi-view model (Pearson’s *r* = −0.6). Importantly, this did not hold vice versa, and Factor 6 (Appendix Fig. S5) in the RNA model was only weakly correlated to the factors learned without RNA (absolute Pearson’s *r* ≤0.09). This demonstrates that MANTRA can leverage information from the transcriptomic view to uncover clinically relevant patient stratification that was hidden when analyzing the drug response data alone by explicitly modeling the higher-order structure of the drug response data.

We next quantified how well MANTRA was able to learn a discriminative representation that was able to separate patients based on IGHV status and compared the this to state-of-the-art multi-omics matrix factorization tools MOFA+ (Argelaguet et al, 2018, 2020) and MOFA-FLEX (Qoku et al, 2025), using the drug response tensor as well as the RNA data as input (treating each slice in the tensor as a data view). To this end, we followed standard practice from the omics data integration literature (Luecken et al, 2022); we first clustered the learnt latent space using leiden clustering and then quantified how well clusters aligned with mutation status by computing the adjusted Rand index (ARI) as well as the Normalized Mutual Information (NMI) (Fig. 4). This confirmed that (i) MANTRA was able to learn better representations (in terms of clustering performance) compared to other multi-view integration methods and that (ii) the MANTRA model with RNA learnt quantifiably better representations than the model trained without RNA information. Similarly, we found the MANTRA also clustered patients by Trisomy 12 status better than baselines (Appendix Figs. S6 and S7). Trisomy 12, present in approximately 10–20% of CLL patients, is an intermediate-risk cytogenetic abnormality associated with atypical immunophenotype and distinct drug response profiles (Quijano et al, 2018). The ability of MANTRA to learn patient embeddings that separate patients by established prognostic markers IGHV and Trisomy 12 demonstrates that the model captures clinically meaningful axes of biological variation relevant to disease stratification and treatment response.

### MANTRA disentangles cell-type-specific disease patterns in single-cell multi-omics data

**Figure 5:**
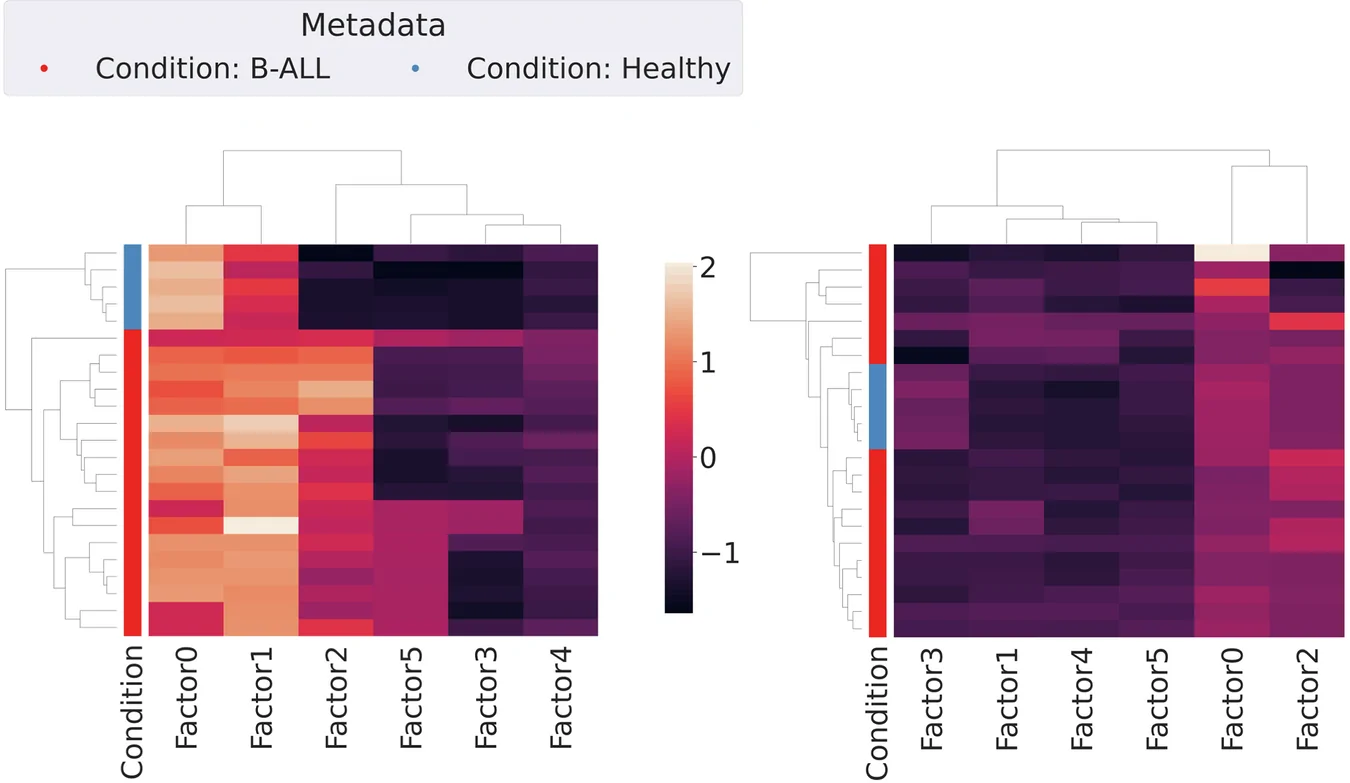
Joint RNA-seq and ATAC-seq analysis reveals coherent multi-omic factor latent space. Factor scores for a single-view MANTRA model learned on the RNA-seq tensor only (left) and a multi-view MANTRA model integrating RNA-seq and ATAC-seq data (right). Both models clearly separate healthy donors from ALL patients and reveal heterogeneity between patients.

To showcase the applicability of MANTRA to model high-dimensional single-cell data, we analyzed a multi-omics dataset from pediatric Acute Lymphoblastic Leukemia (ALL) patients and healthy donors (Chen et al, 2022). We created two third-order tensors (patients × cell types × features), one for scRNA-seq and one for scATAC-seq, by aggregating normalized values on a patient and cell type level as cell-type-specific pseudo-bulk data (Mitchel et al, 2025) while allowing for missing values if a cell type was not observed in a given patient. We first ran MANTRA on the RNA data only to establish a baseline and facilitate comparison to scITD. We thereby aimed to identify coordinated patterns of variation across patients, cell types, and molecular modalities that are associated with the disease state. Since scITD requires all cell types to be observed in all donors (i.e., no missing values in the tensor), it filtered out 7 donors and ran on 11 B-all patients and 5 healthy donors only. Both MANTRA and scITD were able to learn patient embeddings that separate healthy individuals from ALL patients (Figs. 5, left and 6A). However, only MANTRA further stratified the ALL patients into two distinct subgroups, a separation primarily driven by Factor 5 (Figs. 5, left and 7A,B).

**Figure 6:**
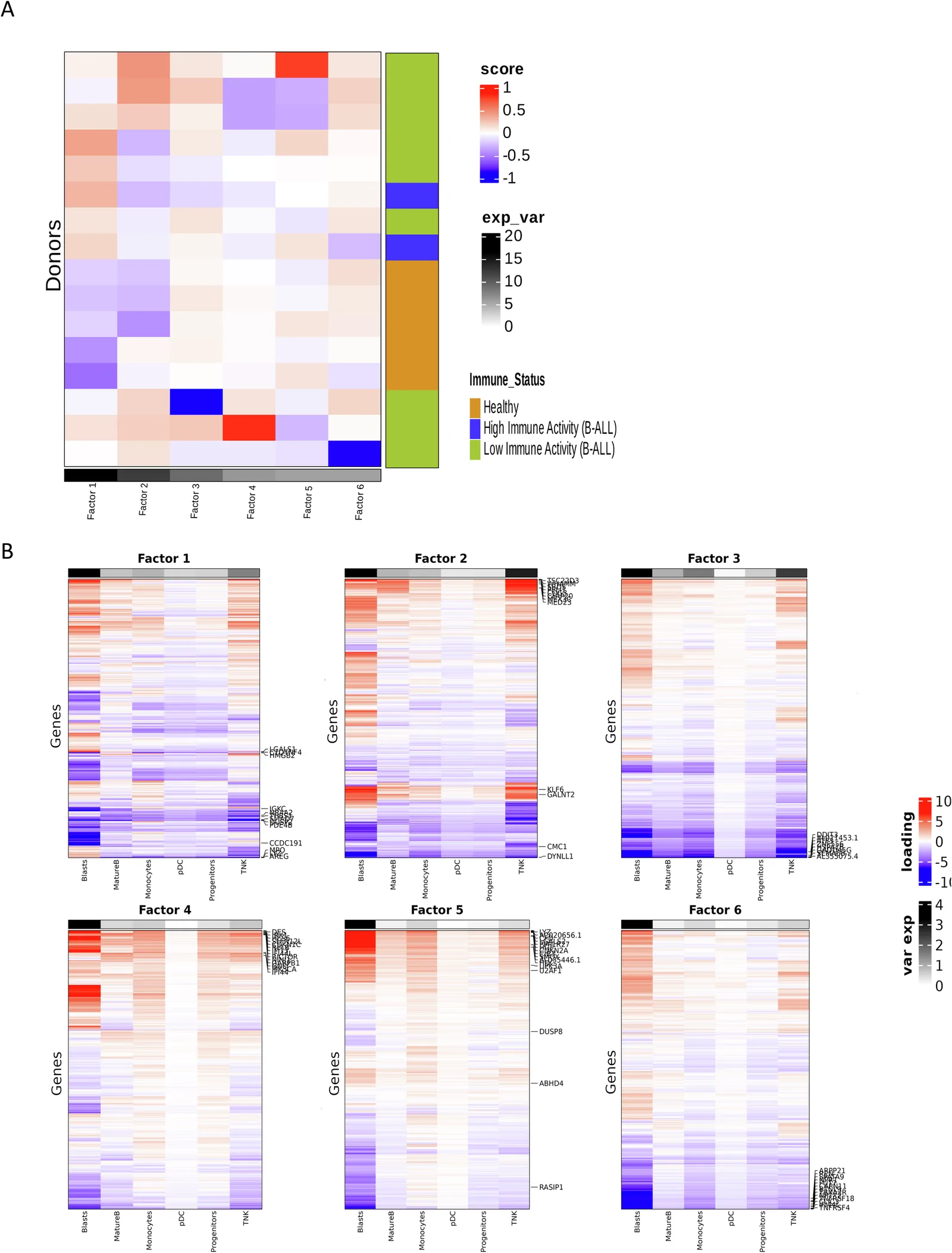
scITD does not recover the pDC-associated factor identified by MANTRA. (**A**) Donor factor scores. scITD separates healthy donors from ALL patients via Factor 1 but does not recover the additional structured inter-patient variation identified by MANTRA. (**B**) Cell type–gene factor loadings learned by scITD, which primarily capture variation driven by blasts and do not identify the pDC-associated factor recovered by MANTRA.

**Figure 7:**
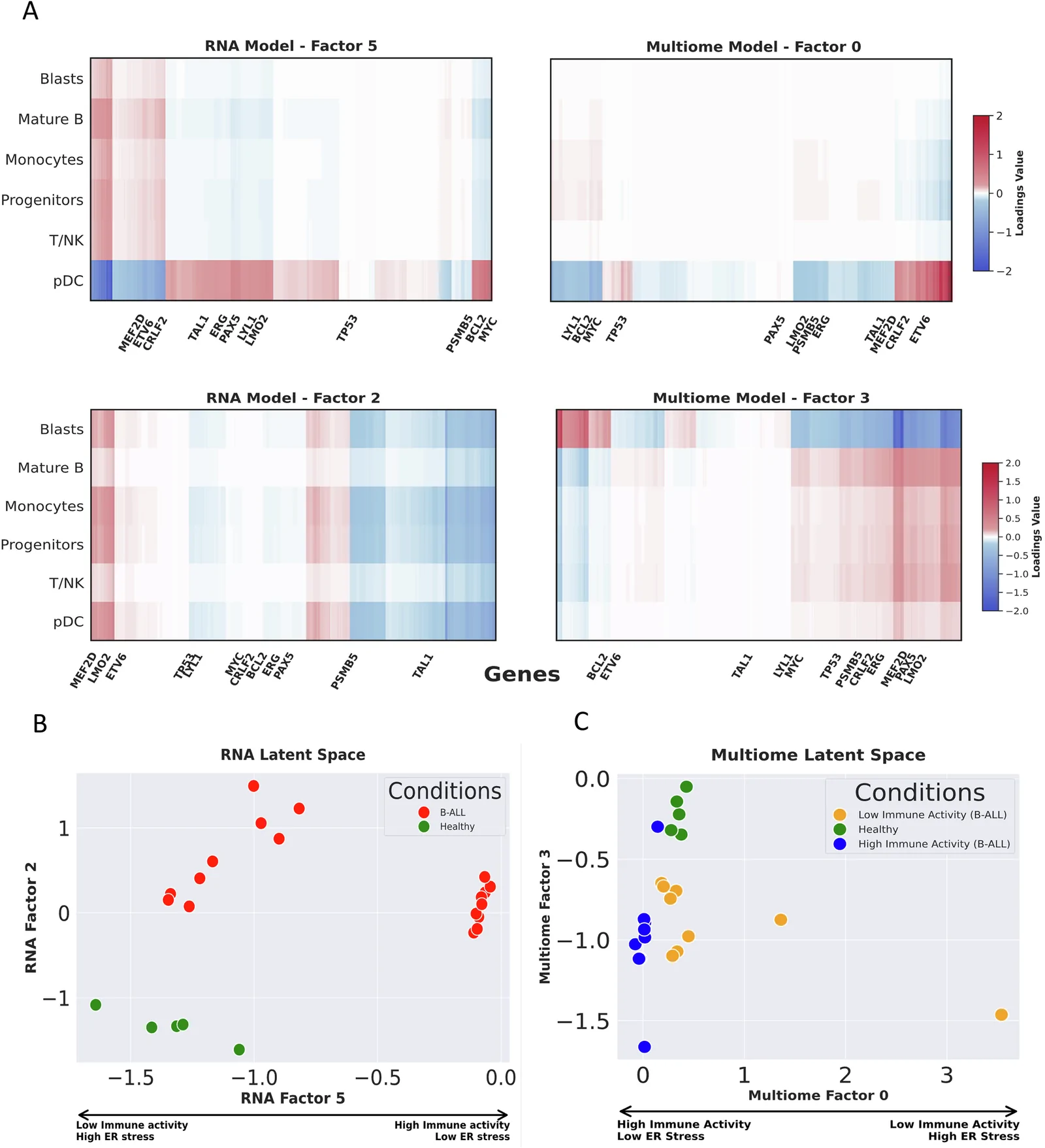
MANTRA resolves clinically relevant subpopulations within B-ALL while distinguishing them from healthy donors. (**A**) Cell-type loading plots for the RNA model (left) show that Factor 5 is primarily active in pDCs, whereas Factor 2 explains variation in most cell types. Cell-type loading plots for the Multiome model (right) show patterns that are consistent with the RNA model, with cell-type attributions more focused on individual cell-types than for the RNA model. (**B**) RNA Latent Space scatter plot separates healthy donors (green) from B-ALL patients (red) and reveals a B-ALL sub-population characterized by high-immune activity/low ER stress. (**C**) The Multiome Latent Space scatter plot demonstrates the consistency between embeddings. Patients from the high-immune activity/low ER stress cluster identified in the RNA latent space are shown in blue, while patients from the low-immune activity/high ER stress cluster are shown in yellow. Healthy donors are shown in green.

**Figure 8:**
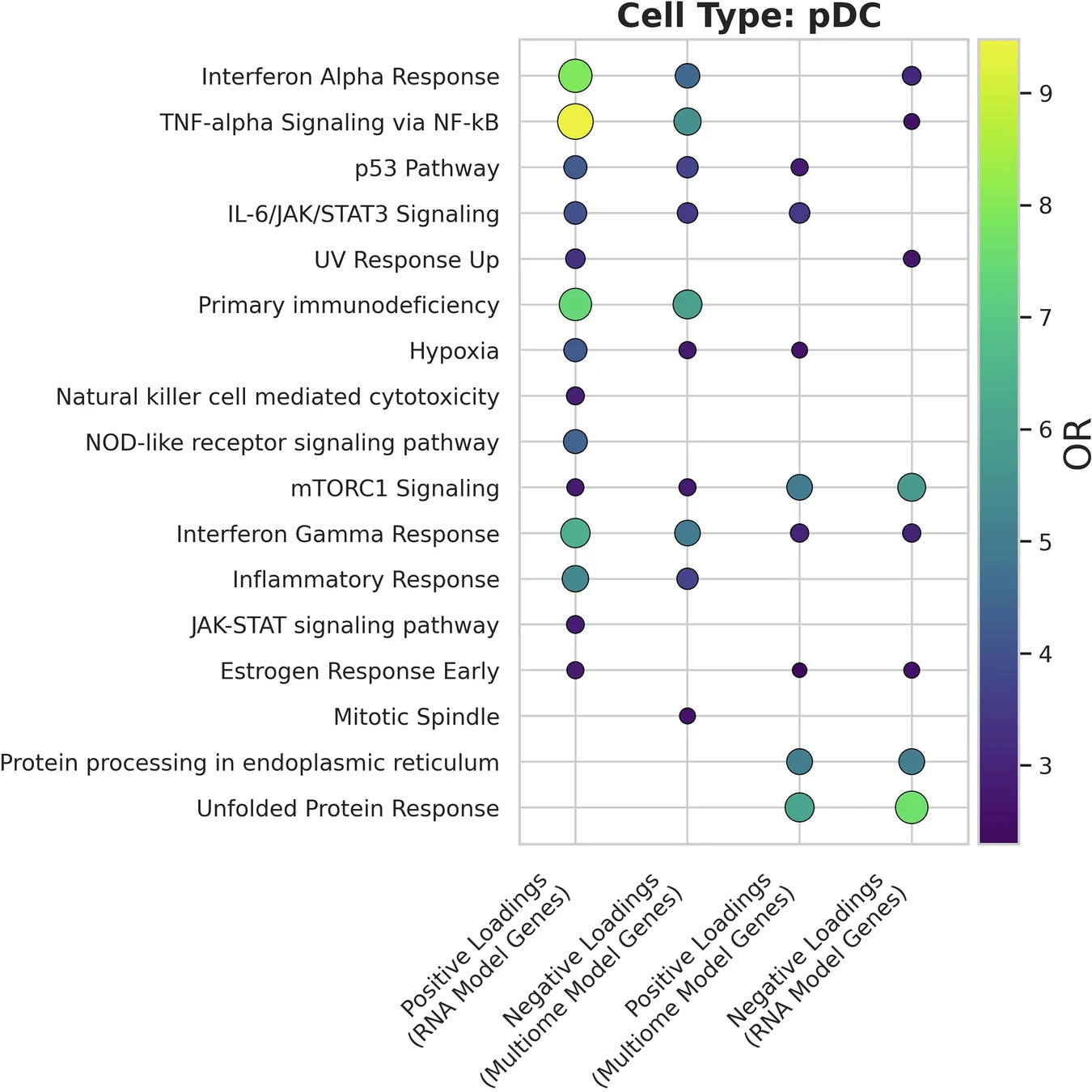
Gene loadings of the pDC-associated factor are enriched for immune and ER stress-related pathways. Pathway enrichment analysis of the loadings of the pDC factor via over-representation analysis (ORA). We performed separate ORA enrichments for positive and negative loadings, for both the RNA-based MANTRA model and the multi-view MANTRA model.

To understand the biological basis of this factor, we examined the corresponding cell-type loadings. This revealed that Factor 5 was predominantly active in plasmacytoid dendritic cells (pDCs), showing a pronounced contrast between pDCs and all other cell types (Fig. 7A, top left). Pathway analysis of the genes associated with Factor 5 in pDCs via its cell-type and gene-specific loadings underscored the biological distinction between these two patient subgroups. Factor loadings were highly enriched in immune-active pathways such as Interferon signaling as well as pathways related to ER stress (Fig. 8; Appendix Fig. S8). At the gene level, this corresponded to low factor loadings of lymphoid transcription factors like ETV6 and higher loadings for genes typically implicated in T-cell lineage ALL, such as BCL2 and MYC, as well as LYL1 (Roberts 2018; Inaba and Mullighan 2020).

Factor 2 (Fig. 7A, bottom left) was the main MANTRA factor that drive the separation between the healthy and ALL patients (Fig. 7B) Pathway analysis of Factor 2 revealed reduced enrichment of cell-cycle–associated pathways together with increased enrichment of ribosomal and translational programs (Appendix Fig. S9), a combination that has been linked to stem and progenitor-like cellular states in blasts in prior single-cell studies of young KMT2A-rearranged ALL patients (Chen et al, 2022). Notably, scITD identified only the major source of variation between patients and healthy donors driven by blasts and did not identify the pDC factor stratifying patients that MANTRA had revealed, as all scITD factors were mainly active in blast cells (Fig. 6B). In addition, running MOFA+ and MOFA-FLEX on the multi-view data in a slice-wise manner did not reveal the pDC factor (Appendix Fig. S10).

We next integrated the RNA-seq tensor and the ATAC-seq tensor using MANTRA. The resulting Multiome model was globally refined to the factor values (Fig. 5, right). MANTRA again identified a pDC-driven factor (Factor 0) (Fig. 7A, top right) separating patients along an axis defined by immune activity and ER stress (Figs. 7C and 8). Compared to the RNA model, the Multiome model discovered two outlier patients in the low-immunity B-ALL cohort (Fig. 7C). Similarly, it identified a factor separating healthy donors from ALL patients, which was mainly active in blasts (Factor 3) (Fig. 7A, bottom right). Pathway analysis of Factor 3 confirmed the HSPC-like program and in addition showed enrichment for immune-related processes, suggesting the presence of a regulatory potential not fully reflected at the transcriptional level (Appendix Fig. S9). Note that due to the sign-invariance of factor models, the signs of the loadings as well as factor scores are flipped in comparison to the RNA model (i.e., for the RNA model, patients with high-immune activity have high factor scores, while for the Multiome model, patients with high-immune activity have low factor scores). These findings suggest that MANTRA was able to capture a clinically relevant axis of heterogeneity in ALL, which orders patients along a continuum spanning from high-immune activity to low-immune activity within pDCs.

**Figure 9:**
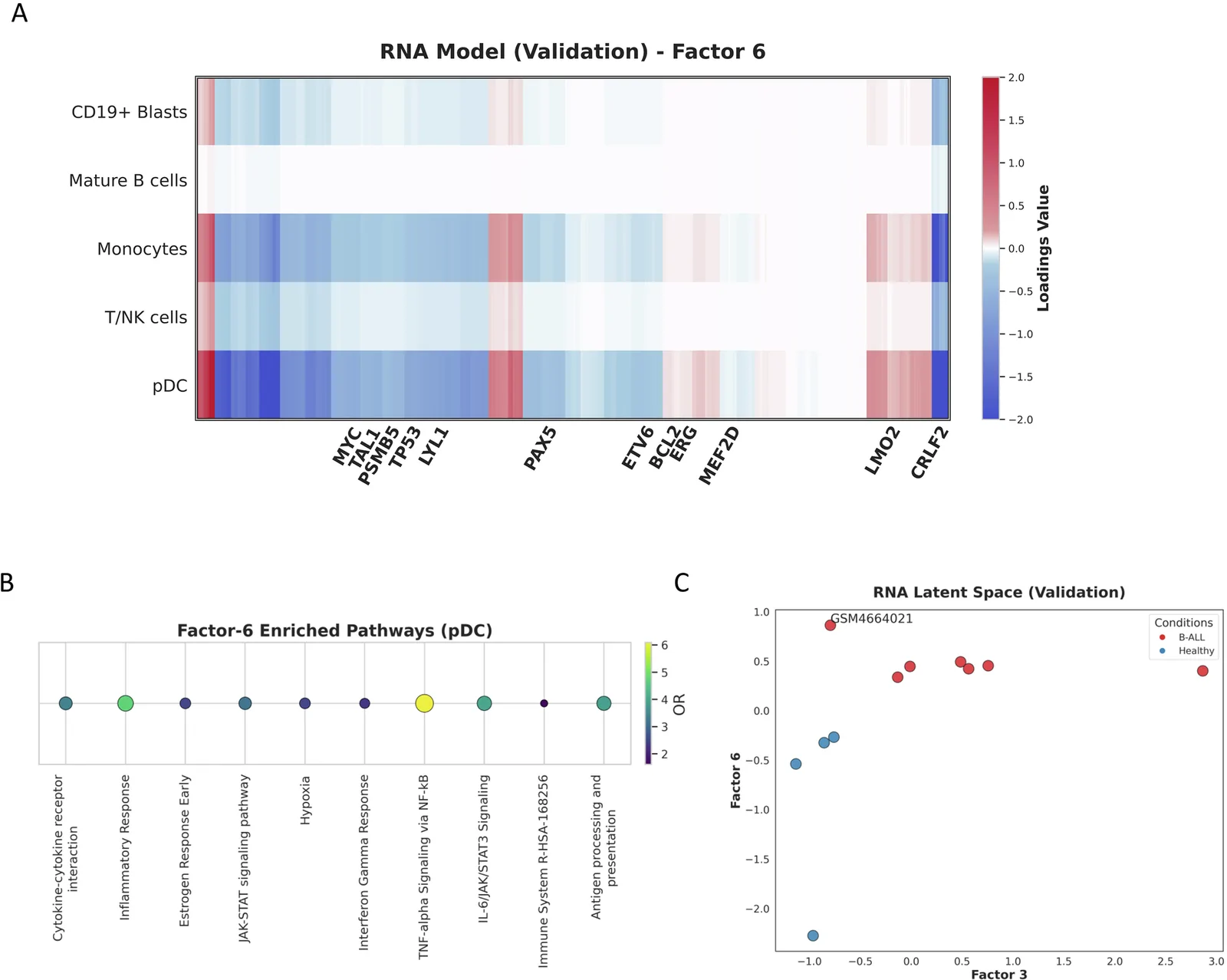
The pDC-associated factor generalizes to an independent pediatric B-ALL cohort. (**A**) MANTRA identifies a latent factor primarily driven by pDCs when applied to the RNA-seq tensor from an independent pediatric B-ALL cohort. (**B**) Over-representation analysis (ORA) of gene loadings reveals enrichment of immune-related pathways, consistent with findings from the discovery cohort. (**C**) Scatter plot showing separation between healthy donors and B-ALL patients. The patient with the lowest immune activity (the highest score of Factor 6) is highlighted.

To validate this hypothesis in an external patient cohort, we analyzed scRNA-seq data of an independent cohort of 7 pediatric B-ALL patients and 4 healthy donors (Bailur et al, 2020). We ran MANTRA on the patients×cell types×genes tensor, which again revealed that one of the factors learned by MANTRA was primarily active in pDCs (Fig. 9A). We next investigated whether this factor was again driven by pathways related to immune activation. Supporting our hypothesis derived from the discovery cohort, pathway analysis of the loadings again identified immune-active pathways similar to those identified in the discovery cohort (Figs. 8 and 9B).

To further assess the consistency between the MANTRA models learned on the discovery and validation cohorts, respectively, we projected the donors from the validation cohort into the MANTRA space learned on the discovery cohort (Appendix Fig. S11). To this end, we froze both cell type loadings and gene loadings, learning only patient-level scores by optimizing the ELBO. Projected donor scores matched those of the immune activity levels learned directly from the validation cohort. That is, validation patients for which MANTRA inferred high pDC immune activity when trained directly on the validation cohort, clustered with discovery patients with high pDC immune activity when projected onto the MANTRA space learned from the discovery cohort (Fig. 9C; Appendix Fig. S11). Similarly, healthy donors from the validation cohort clustered with healthy donors from the discovery cohort. Taken together, this confirmed that pDCs are a driver of inter-patient heterogeneity in pediatric B-ALL patients, separating patients with pDCs in a high-immune-active and low-immune-active state. Importantly, when applying MANTRA to a cohort of pediatric T-ALL patients (Bhasin et al, 2023) as a negative control, we did not find such pDC factor driving inter-patient variability (Appendix Fig. S12), suggesting that our finding is specific to B-ALL. These findings demonstrate MANTRA’s ability to deconvolve complex multi-omics data to pinpoint cell-type-specific molecular signatures that stratify patients.

## Discussion

We have developed MANTRA, a Bayesian framework for integrating multi-modal datasets that are naturally represented as a collection of tensors of varying orders. MANTRA has diverse use cases ranging from drug–response studies to single-cell multi-omics data and generalizes tensor decomposition tools as well as group factor analysis-based approaches. We demonstrate the broad applicability of MANTRA on two real-world datasets and show that the ability to jointly decompose tensors of different ranks is crucial to identifying patient embeddings that are associated with relevant clinical covariates.

MANTRA is an unsupervised model and as such able to identify multicellular patterns with high variance across patients. However, this means that not all factors will be linked to known covariates, and while the extension of this work towards supervised factorizations is in principle possible via the introduction of additional terms to the loss function (Lee et al, 2024), this comes with new challenges around overfitting and is something we leave to future work. The linear nature of MANTRA makes the model inherently interpretable and can reveal biological pathways driving the observed co-variation patterns via a generalized loadings matrix. To comprehensively reveal non-linearities in such complex datasets would require adapting the factorization process, for example, via non-linear kernels, but this would likely come at the cost of the model’s interpretability, which is a key strength of our approach.

Despite its conceptual and practical advantages, MANTRA has certain limitations that warrant consideration. First, similar to other latent variable and factorization frameworks, the model faces inherent challenges regarding identifiability and rotational invariance. Second, there are specific settings where mixed-order modeling may not substantially outperform standard matrix-based approaches. In datasets where structural dependencies are negligible, or where extreme sparsity and high noise levels impede the robust estimation of multi-dimensional components, simpler matrix factorizations may prove equally effective or more stable. Finally, while the fully probabilistic Bayesian inference introduces a higher baseline wall-clock time compared to standard deterministic methods like PARAFAC, MANTRA exhibits highly favorable scaling properties. Thus, while applying the model to population-level atlases may eventually necessitate further algorithmic optimizations, MANTRA efficiently scales to large, complex multi-omics datasets.

In summary, we present MANTRA, a probabilistic framework for multi-view tensor decomposition that accommodates collections of tensors with varying orders and incorporates structured sparsity priors for interpretability. We have demonstrated that MANTRA enables the discovery of biologically meaningful molecular patterns linked to specific data dimensions, such as cell types or drug conditions. By extending beyond the single-view setting of classical tensor decomposition and explicitly modeling the higher-order interactions lost in matrix-based methods like MOFA-FLEX, MANTRA fills an important gap in the multi-omics analysis toolkit.

## Methods

### MANTRA framework

MANTRA (Multi-view ANalysis with Tensor and matRix Alignment) unifies the statistical frameworks of group factor analysis and tensor decomposition into a Bayesian approach for multi-modal data integration. It can be interpreted as a generalization of group factor analysis (e.g., MOFA (Argelaguet et al, 2018)) to tensor-valued data, or equivalently as a generalization of tensor decomposition methods (e.g., PARAFAC (Kossaifi et al, 2019) and scITD (Mitchel et al, 2025)) to a multi-view setting in which multiple datasets of potentially different orders are modeled jointly.

The model is designed to address key requirements of multi-modal studies, including: (i) scalable training via variational inference, (ii) inference of interpretable embeddings through structured sparsity, (iii) principled handling of missing values, and (iv) flexible integration of tensors of different orders. MANTRA takes as input a collection of tensors, each representing a data view (e.g., an omics modality), and jointly decomposes them into shared low-dimensional latent factors across each mode (e.g., patients, tissues, drugs, or genes). These latent factors are inferred within a Bayesian framework using variational inference, enabling efficient optimization and robust uncertainty handling. To promote interpretability, the model incorporates structured sparsity priors that encourage selective activation of relevant features, thereby identifying the main drivers of variation across views. In this work, we distinguish between embeddings and loadings as follows: embeddings (or factor scores) denote the latent representation of each mode, while loadings correspond to interactions between factor matrices across modes.

### Model specification

Let $G=\{{{{{\boldsymbol{Y}}}}}^{{{{\bf{1}}}}},\ldots ,{{{{\boldsymbol{Y}}}}}^{{{{\boldsymbol{M}}}}}\}$ be a collection of tensors of rank 2 (matrices) and rank 3. Let ${G}_{T}=\{{{{{{\boldsymbol{Y}}}}}_{{{{\boldsymbol{T}}}}}}^{({m}_{T})}\}$ be the subset of ${M}_{T}$ three-way tensors of dimension $N\times J\times {K}_{T}^{m}$, with *N* samples (e.g., patients), *J* slices (e.g., tissues), and ${K}_{T}^{m}$ features (e.g., genes). Let ${G}_{M}=\{{{{{{\boldsymbol{Y}}}}}_{{{{\boldsymbol{M}}}}}}^{(m)}\}$ be the subset of ${M}_{M}$ two-way tensors of dimension $N\times {K}_{M}^{m}$, with *N* samples and ${K}_{M}^{m}$ features, such that ${G}_{T}\cup {G}_{M}=G$.

Note that all tensors in ${G}_{T}$ share the number of samples *N* and slices *J*, but have different feature sets. All matrices in ${G}_{M}$ share the number of samples *N* with all tensors in *G*, but have different feature sets. MANTRA jointly decomposes these tensors as follows:

$$\begin{array}{c}{y}_{{ijk}}^{({m}_{T})}={\sum }_{r=1}^{R}{a}_{{ir}}{b}_{{jr}}{c}_{{kr}}^{({m}_{T})}+{\epsilon }_{k}^{({m}_{T})}\\ {y}_{{il}}^{({m}_{M})}={\sum }_{r=1}^{R}{a}_{{ir}}{c}_{{lr}}^{({m}_{M})}+{\epsilon }_{l}^{({m}_{M})}\end{array}$$

where ${{{{\boldsymbol{a}}}}}_{i}$ is the *R*-dimensional embedding for the *i*-th sample (shared across all tensors in *G*), ${{{{\boldsymbol{b}}}}}_{j}$ is the *R*-dimensional embedding for the *j*-th slice (shared across all tensors in ${G}_{T}$), and ${{{{\boldsymbol{c}}}}}_{k}^{({m}_{T})}$ and ${{{{\boldsymbol{c}}}}}_{l}^{({m}_{M})}$ are the view-specific *R*-dimensional feature embeddings for tensors in ${G}_{T}$ and ${G}_{M}$, respectively. The terms ${\epsilon }_{k}^{({m}_{T})}$ and ${\epsilon }_{l}^{({m}_{M})}$ denote feature- and view-specific residual noise. We assume a Gaussian noise model with variance ${\sigma }_{k}^{2(m)}$ and place a conjugate inverse Gamma prior on the variance and normal priors on the sample and slice embeddings (see Supplement for details). For clarity, we refer to the latent representations (***A***, ***B***, ***C***) as “embeddings”, and the product of two embedding matrices as “loadings”.

### Structured sparsity via horseshoe priors

To learn interpretable factors and disentangle view-specific effects, we place a multi-scale shrinkage prior on the feature embedding matrices ${{{{\boldsymbol{C}}}}}^{(m)}$. This is achieved using a Horseshoe prior, which is well-suited for variational inference (Carvalho et al, 2010; Qoku and Buettner, 2023). Specifically, each element of ${{{{\boldsymbol{C}}}}}^{(m)}$ is modeled as:

$${c}_{k,r}^{(m)}{{{\mathscr{\sim }}}}{{{\mathscr{N}}}}\left(0,{\left({\tau }^{(m)}{\delta }_{r}^{(m)}{\lambda }_{k,r}^{(m)}\right)}^{2}\right),$$

where we place positive Cauchy priors on each scale parameter:

$$\begin{array}{ccc}{\tau }^{(m)}\sim {{{{\mathscr{C}}}}}^{+}(0,1), & {\delta }_{r}^{(m)}\sim {{{{\mathscr{C}}}}}^{+}(0,1), & {\lambda }_{k,r}^{(m)}\sim {{{{\mathscr{C}}}}}^{+}(0,1).\end{array}$$

Here, ${\tau }^{(m)}$ is a global scale parameter for view *m*. The term ${\delta }_{r}^{(m)}$ acts as an Automatic Relevance Determination (ARD) prior (MacKay, 1994) for factor *r* in view *m*, allowing factors to be active in only a subset of views. The term ${\lambda }_{k,r}^{(m)}$ provides local, element-wise regularization, encouraging sparsity within each factor.

#### Variational inference

We approximate the posterior distributions of the variables of interest ${{{\boldsymbol{\Theta }}}}$ by introducing a fully factorized family of parameterized distributions, where ${{{\boldsymbol{\Theta }}}}={{{\boldsymbol{A}}}},{{{\boldsymbol{B}}}},{{{\boldsymbol{C}}}},{{{\boldsymbol{\Lambda }}}},{{{\boldsymbol{\Delta }}}},{{{\boldsymbol{\tau }}}},{{{\boldsymbol{\Psi }}}}$, and

$$\begin{array}{c}{{{\boldsymbol{\Psi }}}}=\left\{{\sigma }_{k}^{(m)}\right\},{{{\boldsymbol{A}}}}=\left\{{a}_{i,r}\right\},{{{\boldsymbol{B}}}}=\left\{{b}_{j,r}\right\},{{{\boldsymbol{C}}}}=\left\{{c}_{k,r}^{(m)}\right\}\\ {{{\boldsymbol{\Lambda }}}}=\left\{{\lambda }_{k,r}^{(m)}\right\},{{{\boldsymbol{\Delta }}}}=\left\{{\delta }_{r}^{(m)}\right\},{{{\boldsymbol{\tau }}}}=\left\{{\tau }^{(m)}\right\}.\end{array}$$

This is achieved by maximizing the evidence lower bound with respect to variational parameters. This, in turn, reduces the gap between the true and the approximate posterior in terms of the KL divergence. We use the family of normal distributions for approximating ${{{{\boldsymbol{C}}}}}^{(m)}$, ***A*** and ${{{{\boldsymbol{B}}}}}^{(m)}$; for the rest of the parameters, we use the log-Normal distribution to ensure positive samples (Ghosh et al, 2018).

### Synthetic data generation

To validate model performance in a controlled setting, we generated synthetic data following a fixed pipeline. First, ground-truth factor matrices (***A***, ***B***, ***C***) were sampled from a standard normal distribution. Second, sparsity was introduced directly in the factor matrices via random dropout (i.e., setting a fraction of entries to zero). Sparsity levels were chosen to reflect real-world patterns observed in ALL data (12% for RNA and 32% for ATAC), with a default sparsity of 0.1. Third, a clean third-order tensor ${{{\mathscr{T}}}}$ was constructed as the outer product of the generated factor matrices.

To evaluate robustness across diverse data regimes, we systematically varied key simulation parameters: (i) the number of latent factors (default: 20) to control underlying complexity, (ii) the number of samples (default: 20) to reflect dataset size, (iii) sparsity (default: 0.1) to mimic varying degrees of missingness, (iv) signal-to-noise ratio (default: 10 dB) to model measurement quality, (v) number of features (default: 5000) to capture high-dimensional settings, and (vi) Gaussian noise level (default *σ*_noise_ = 1) to control noise magnitude.

To enforce a constant signal-to-noise ratio (SNR) across different ranks, we next rescaled the clean tensor before adding noise. The mean signal power of ${{{\mathscr{T}}}}$ was computed as

$${P}_{{{{\rm{current}}}}}=\frac{1}{N}{\sum }_{i,j,k}{{{{\mathscr{T}}}}}_{i,j,k}^{2}=\frac{{{||}{{{\mathscr{T}}}}{||}}_{F}^{2}}{N},N=I\cdot J\cdot K$$

Given the per-entry Gaussian noise standard deviation ${\sigma }_{{{\rm{noise}}}}$ (noise power ${\sigma }_{{{\rm{noise}}}}^{2}$) and the target SNR in dB, ${{{{\rm{SNR}}}}}_{{{{\rm{dB}}}}}$, the desired signal power is

$${P}_{{{{\rm{target}}}}}={\sigma }_{{{{\rm{noise}}}}}^{2}\cdot {10}^{{{{{\rm{SNR}}}}}_{{{{\rm{dB}}}}}/10}$$

Scaling tensor entries by a factor *f* scales the signal power by ${f}^{2}$, hence

$$f=\sqrt{\frac{{P}_{{{{\rm{target}}}}}}{{P}_{{{{\rm{current}}}}}}}$$

Because the tensor is trilinear in the factor matrices, scaling each factor by *γ* scales the tensor by ${\gamma }^{3}$, giving

$$\gamma ={f}^{1/3}={\left(\frac{{P}_{{{{\rm{target}}}}}}{{P}_{{{{\rm{current}}}}}}\right)}^{1/6}$$

We then reconstructed the scaled clean tensor and finally added i.i.d. Gaussian noise with standard deviation ${\sigma }_{{{\rm{noise}}}}$ to obtain the observed tensor. This SNR definition follows standard signal-processing conventions (Oppenheim and Schafer, 2014).

We also added an additional experiment to mimic real-world applications where it is unclear what the true rank of the data is by simulating data with rank 20 and training tensor factorization models with up to 40 latent factors. To disentangle the effect of these individual factors, we chose a default (given in parentheses above) and varied one factor at a time. RMSE was calculated relative to the norm of ${{{\mathscr{T}}}}$ to ease interpretability. This allowed us to assess the model’s ability to accurately reconstruct the data and recover the ground-truth factors when compared to standard PARAFAC.

#### Multi-view data generation

For the multi-view benchmark, we generated two synthetic multi-view tensors sharing the same sample and condition modes but differing in their true latent rank. Each dataset contained 20 samples and 5 conditions, with 3000 features in first view and 5000 features in the second view. Sparse Gaussian factor matrices were generated with sparsity fixed at 0.3, and the two views were assigned rank pairs of (5,10), (5,20), (5,30), (10,20) and (10,30). Clean tensors were constructed from these factors, scaled to a target signal-to-noise ratio of 10 dB, and corrupted with Gaussian noise with standard deviation 1.0. For each rank-gap setting, we ran 5 trials using different random seeds and fitted each dataset at a rank equal to the larger of the two true ranks. We compared native multi-view MANTRA and TensorLy PARAFAC applied to the concatenated tensor. Performance was assessed using overall and per-view RMSE, and effective rank recovery was estimated from the feature-mode factor matrix.

#### Scalability analysis

We compared MANTRA and TensorLy PARAFAC by measuring wall-clock runtime and per-epoch (MANTRA)/per-iteration (PARAFAC) time on synthetic tensors, varying one problem dimension at a time while holding the others fixed. Datasets were generated with 20 samples, 5 conditions, 5000 features, rank 20, a target signal-to-noise ratio (SNR) of 10 dB, noise standard deviation of 1.0, 5000 training epochs for MANTRA (early stopping was deactivated to be consistent between runs), and three independent trials per setting using different random seeds. Sparsity levels of 0.1 and 0.3 were evaluated separately. The scaling dimensions were feature size (1000, 2000, 5000, 10,000), number of patients (10, 20, 50, 100, 200), and CP rank (10, 20, 30, 50). All experiments were executed on a dedicated GPU node of an HPC cluster equipped with two Intel Xeon Silver 4216 processors (32 physical cores/64 threads total) and an NVIDIA Quadro RTX 5000 GPU (16 GB GDDR6). MANTRA was accelerated on the GPU, whereas TensorLy PARAFAC was executed on the CPU, as GPU execution was not used in that baseline implementation. Wall-clock runtimes were measured using time.perf_counter (), with torch.cuda.synchronize () calls ensuring correct GPU timing for MANTRA.

#### Hyperparameter stability analysis

Hyperparameter stability was assessed by evaluating MANTRA across a grid of inverse-Gamma prior hyperparameters under varying sparsity and noise conditions. Synthetic tensors were generated with 20 samples, 5 conditions, 5000 features, and true rank 20 using sparse Gaussian factor matrices, then rescaled to fixed target signal-to-noise ratios before adding Gaussian noise with standard deviation 1.0. The hyperparameter sweep tested five $(\alpha ,\beta )$ pairs, (0.5,0.25), (1.0,0.5), (2.0,1.0), (3.0,1.5), and (5.0,2.5), across sparsity levels of 0.1 and 0.3 and SNR values of 2, 5, 10, 15, 20, and 25 dB. For each (${{\rm{sparsity}}}$,${{\rm{SNR}}}$,*α*,*β*) combination, 5 replicate datasets were generated using different random seeds. For each replicate, MANTRA was fit to the noisy tensor with rank fixed at 20 for 5000 epochs using one particle, learning rate 0.005, clipped optimization, and GPU execution. Reconstruction performance was quantified primarily against the clean tensor using RMSE metric. This design was used to determine whether MANTRA’s reconstruction accuracy remained stable across prior settings as data sparsity and noise level changed.

### Datasets and preprocessing

We demonstrate the versatility of MANTRA to disentangle inter-patient heterogeneity in complex study designs via two publicly available datasets:

#### CLL drug–response dataset

We used a publicly available dataset from Bruch et al (Bruch et al, 2022) studying Chronic Lymphocytic Leukemia (CLL). The study profiled 192 primary CLL patient samples, measuring cell viability in response to 12 drugs under 17 different microenvironmental stimuli (cytokines). This dataset was complemented by bulk RNA-seq for the same patients. We structured this data into two views:

1. A 3rd-order tensor of (patients × drugs × cytokines) with dimensions 192×12×17, containing the log-normalized cell viability values.
2. A 2nd-order tensor (matrix) of (patients × genes) from the RNA-seq data, containing the log-normalized counts of the 5000 most variable genes.

We ran MANTRA on this multi-view dataset with R=20 latent factors for 7000 epochs with a learning rate of 0.001. For comparison, we also ran a single-view analysis on the drug–response tensor alone for 5000 epochs with a learning rate of 0.005.

#### ALL single-cell multi-omics dataset

We used a single-cell multi-omics dataset from Chen et al (Chen et al, 2022), which profiled 18 pediatric patients with KMT2A-rearranged Acute Lymphoblastic Leukemia (ALL) and 5 healthy donors. We used the provided scRNA-seq and scATAC-seq data. Following Mitchel et al (Mitchel et al, 2025), we generated pseudo-bulk tensors by aggregating the normalized data for each patient and cell type. This resulted in two 3rd-order tensors, one for each modality:

1. An scRNA-seq tensor of (patients × cell types × genes).
2. An scATAC-seq tensor of (patients × cell types × peaks).

For preprocessing, we filtered out cells with fewer than 200 total RNA counts and selected the union of the 4000 most variable features from healthy and ALL samples for each modality. We used the Seurat-normalized values provided in the original study. We trained a first MANTRA model on the RNA tensor only and a second MANTRA model on both tensors; we trained for 3000 epochs with a learning rate of 0.005. To interpret the factor loadings, we performed over-representation analysis on the top/bottom 500 features per cell type using the ‘gseapy’ package. To confirm these analyses, we also performed pre-ranked GSEA directly on the ranked loadings with a diverse set of pathway databases, namely GO (Consortium, 2019), KEGG (Kanehisa et al, 2017), REACTOME (Milacic et al, 2024), Wikipathways (Agrawal et al, 2024), and MSigDB Hallmark/Oncogenic signatures (Liberzon et al, 2015). We ran scITD on the same input tensor, but since scITD is not able to account for missing values, 7 out of 18 patients were filtered out, and the algorithm only analyzed data from 11 patients.

#### B-ALL single-cell validation dataset

For validation, we used scRNA-seq data of 7 pediatric B-ALL patients and 4 healthy donors from an independent cohort (Bailur et al, 2020) and constructed the 3D tensor as described above. We ran MANTRA with the same settings as for the discovery cohort and performed an analogous downstream analysis.

#### T-ALL scRNA-seq dataset

As a negative control, we analyzed a cohort of T-ALL patients (Bhasin et al, 2023). We followed the same training protocol as for the discovery and validation cohorts, followed by the same downstream analysis.

### Comparison with MOFA+, MOFA-FLEX, and scITD

To benchmark MANTRA against a matrix-based method, we compared its performance on the CLL dataset to MOFA (Argelaguet et al, 2018). Since MOFA cannot model tensor data directly, we reshaped the 3rd-order drug–response tensor into a set of 2D matrices. Specifically, we treated the drugs and genes as features and modeled each of the 17 cytokine conditions as a separate view, in addition to the RNA-seq view. This resulted in 18 views in total. We ran the MOFA+ implementation (Argelaguet et al, 2020) with the same number of factors (R=20) as MANTRA and used the same data normalization. Similarly, we compared MANTRA to MOFA-FLEX (Qoku et al, 2025), a factor analysis framework designed for customizable modeling across diverse multi-omics data scenarios. As for MOFA + , we decomposed the 3D tensors into sets of 2D matrices and trained a MOFA-FLEX model using a default configuration.

On the B-ALL data, we further compared MANTRA to scITD; we used the same tensor derived from the scRNA-seq data as input for both tools. We ran scITD with default settings, except for reducing the donor_min_cells parameter to 0 in order to maximize the number of patients that could be analyzed with scITD.

For the CLL dataset, we performed a quantitative evaluation of how well embeddings captured the clinically relevant IGHV status of patients. To this end, we followed approaches for measuring biological conservation (Luecken et al, 2022) and constructed a kNN graph based on the learned sample embeddings, followed by Leiden clustering using scanpy with default settings. We then computed the Adjusted Rand Index (ARI) as well as Normalized Mutual Information (NMI) scores to quantify how well clusters overlapped with mutation status (Luecken et al, 2022).

## Supplementary information

## Data Availability

MANTRA is implemented in Python using the probabilistic programming library Pyro (Bingham et al, 2019). The source code is publicly available on GitHub at https://github.com/MLO-lab/MANTRA. The source data of this paper are collected in the following database record: biostudies:S-SCDT-10_1038-S44320-026-00223-8.
