# Supplementary material for: Interpretable multi-omics integration across mixed-order tensors with MANTRA
Source: Mol Syst Biol. 2026 Jun 18;22(9):1415–29. doi: 10.1038/s44320-026-00223-8 (PMC13538497; doi:10.1038/s44320-026-00223-8)
Supplement: Supplementary file 1 [file 44320_2026_223_MOESM1_ESM.pdf]

Appendix for "Interpretable multi-omics integration across mixed-order tensors with MANTRA"

Table of Contents

A1. Full generative model 2

A2. Joint probability distribution 3

Appendix Figure S1....4

Appendix Figure S2....5

Appendix Figure S3....5

Appendix Figure S4....6

Appendix Figure S5....6

Appendix Figure S6....7

Appendix Figure S7....8

Appendix Figure S8....9

Appendix Figure S9....10

Appendix Figure S10....11

Appendix Figure S11....12

Appendix Figure S12....13

A1. Full generative model

MANTRA is defined as

$$
\begin{aligned}
y_{ijk}^{(m_T)} &\sim \mathcal{N}\left(\sum_{r=1}^{R} a_{ir}\, b_{jr} c_{kr}^{(m_T)}, \sigma_k^{2(m_T)}\right) \\
y_{il}^{(m_M)} &\sim \mathcal{N}\left(\sum_{r=1}^{R} a_{ir}\, c_{lr}^{(m_M)}, \sigma_k^{2(m_T)}\right) \\
a_{ir} &\sim \mathcal{N}(0,1) \\
b_{jr} &\sim \mathcal{N}(0,1) \\
c_{k,r}^{(m)} &\sim \mathcal{N}\left(0, \left(\tau^{(m)} \delta_r^{(m)} \lambda_{k,r}^{(m)}\right)^2\right) \\
\tau^{(m)} &\sim \mathcal{C}^+(0,1) \\
\delta_r^{(m)} &\sim \mathcal{C}^+(0,1) \\
\lambda_{k,r}^{(m)} &\sim \mathcal{C}^+(0,1) \\
\sigma_k^{2(m)} &\sim \Gamma^{-1}(\alpha_\sigma, \beta_\sigma)
\end{aligned}
$$

$\Gamma^{-1}(\alpha, \beta)$ is the inverse-Gamma distribution with shape and scale parameters α and β. We choose $\alpha = 1$ and $\beta = 0.5$.

A2. Joint probability distribution

With θ denoting all parameters in the model, the joint probability distribution can be written as:

$$
\begin{aligned}
p(\{y^{(m_T)}\},\{y^{(m_M)}\},\theta) = & \prod_{m_T}\prod_{i,j,k}\mathcal{N}\left(y_{ijk}^{(m_T)} | \sum_{r=1}^{R} a_{ir}\, b_{jr} c_{kr}^{(m_T)}, \sigma_k^{2(m_T)}\right) \times \\
& \prod_{m_M}\prod_{i,l}\mathcal{N}\left(y_{il}^{(m_M)} | \sum_{r=1}^{R} a_{ir}\, c_{lr}^{(m_M)}, \sigma_k^{2(m_M)}\right) \times \\
& \prod_{i,r}\mathcal{N}\,(a_{ir}|0,1) \times \prod_{j,r}\mathcal{N}\,(b_{jr}|0,1) \times \\
& \prod_{m}\prod_{k,r}\mathcal{N}\left(c_{k,r}^{(m)} | 0, \left(\tau^{(m)}\delta_r^{(m)}\lambda_{k,r}^{(m)}\right)^2\right) \times \\
& \prod_{m}\mathcal{C}^+\,(\tau^{(m)}|0,1) \times \prod_{m}\prod_{r}\mathcal{C}^+\,(\delta_r^{(m)}|0,1) \times \\
& \prod_{m}\prod_{k,r}\mathcal{C}^+\,(\lambda_{k,r}^{(m)}|0,1) \times \\
& \prod_{m}\prod_{k}\Gamma^{-1}\,(\sigma_k^{2(m)}|\alpha_\sigma,\beta_\sigma)
\end{aligned}
$$

Here, the first line represents the likelihood for all rank-3 tensors, the second line represents the likelihood for all matrices. The third line represents the priors for the shared components $\boldsymbol{a}$ and $\boldsymbol{b}$. The fourth line represents the hierarchical prior structure for the view-specific components $\boldsymbol{c}$. The fifth and sixth lines represent the priors for the hierarchical variance component and the last line represents the priors for the observation noise variances.

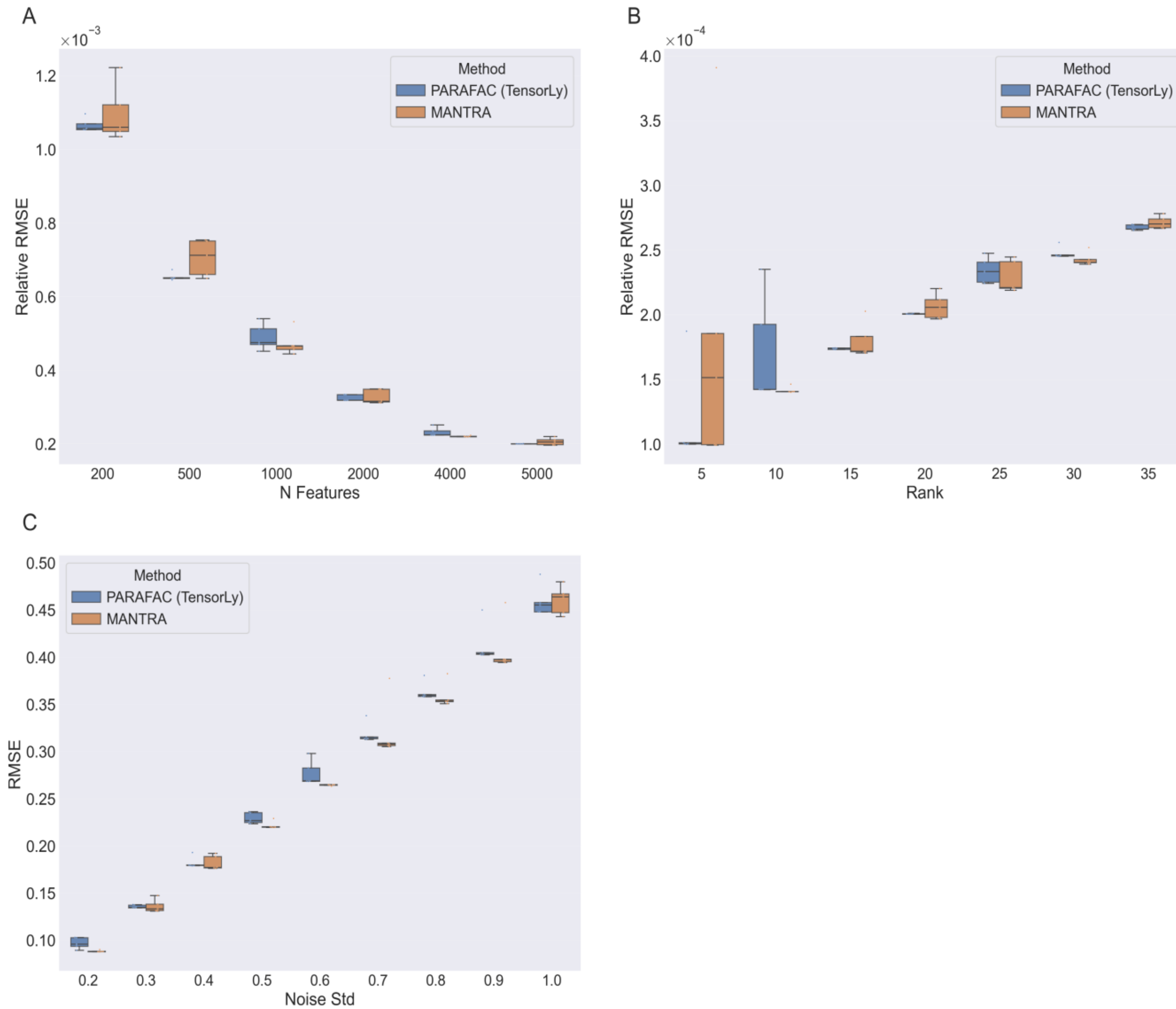

Appendix Figure S1 *Relative RMSE for varying numbers of features (A), rank for simulated data (B) and RMSE for varying random noise (C).*

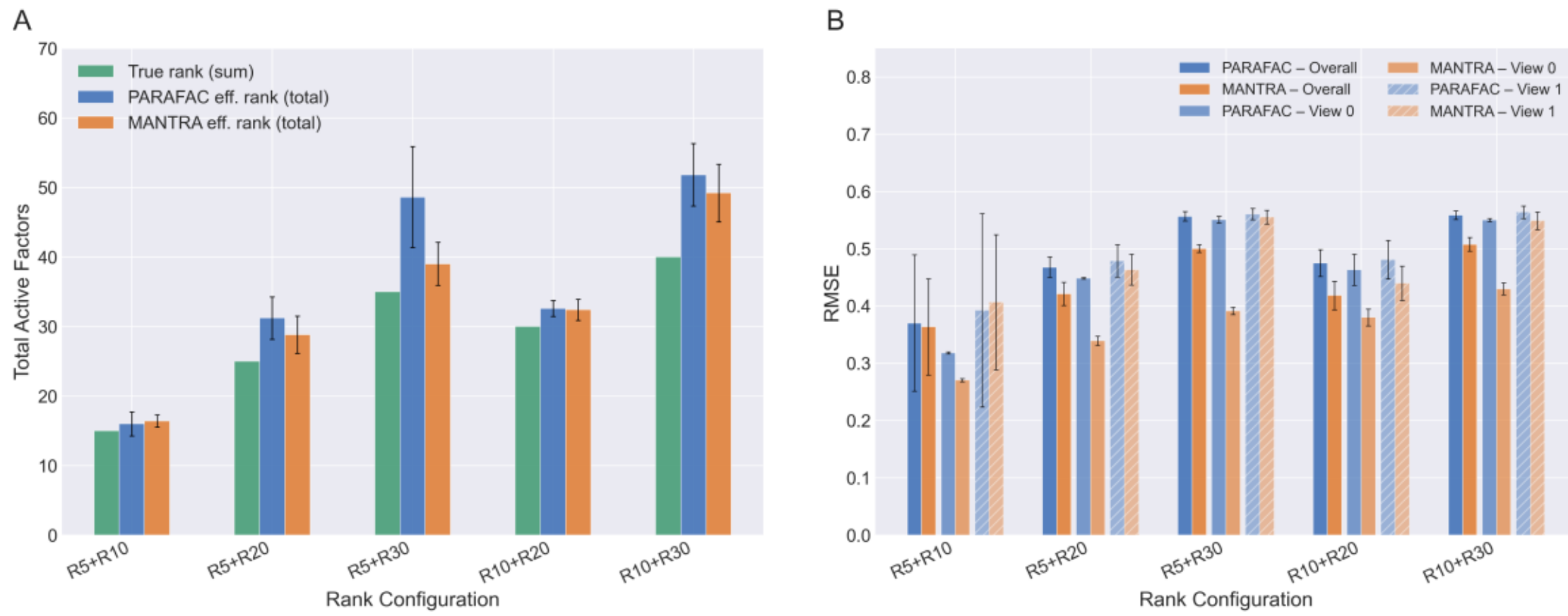

Appendix Figure S2 *Multiview experiment for comparing MANTRAs ability to turn off the factors that are not relevant for specific view. (A) Overall effective rank comparison (B) per view RMSE comparison.*

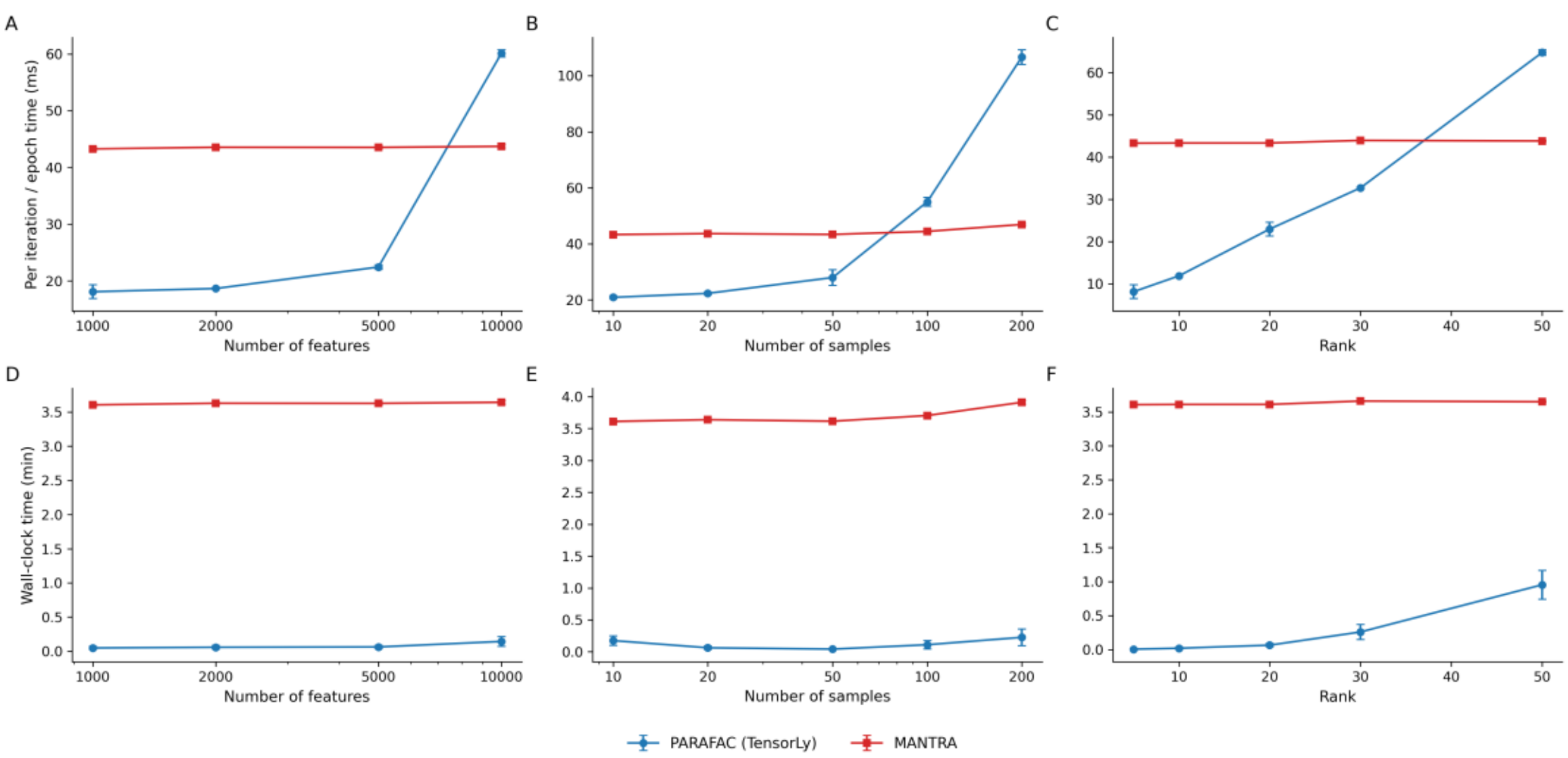

Appendix Figure S3 *Per iteration/epoch time is shown for varying number of features (A), number of samples (B), and rank (C). Total wall-clock time in minutes is shown for varying number of features (D), number of samples (E), and rank (F).*

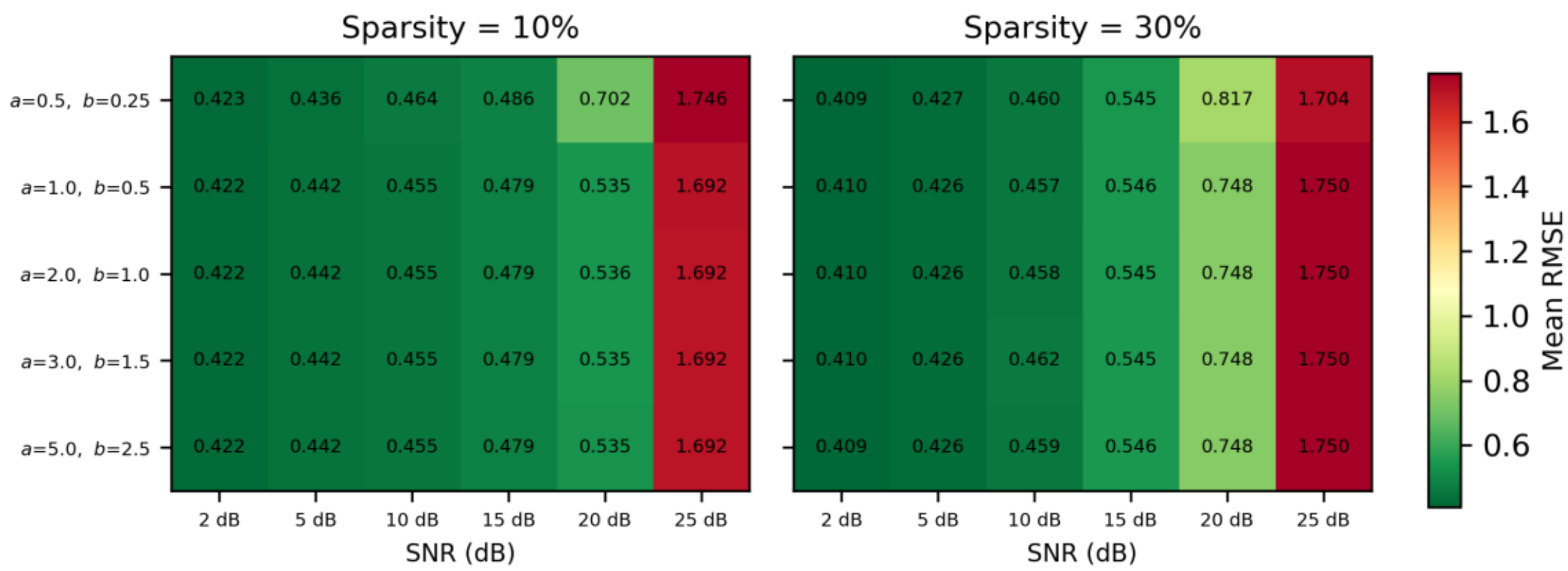

Appendix Figure S4 *Hyperparameter stability analysis of the scale parameters α and β in the MANTRA model for sparsity 0.1 (left) and 0.3 (right).*

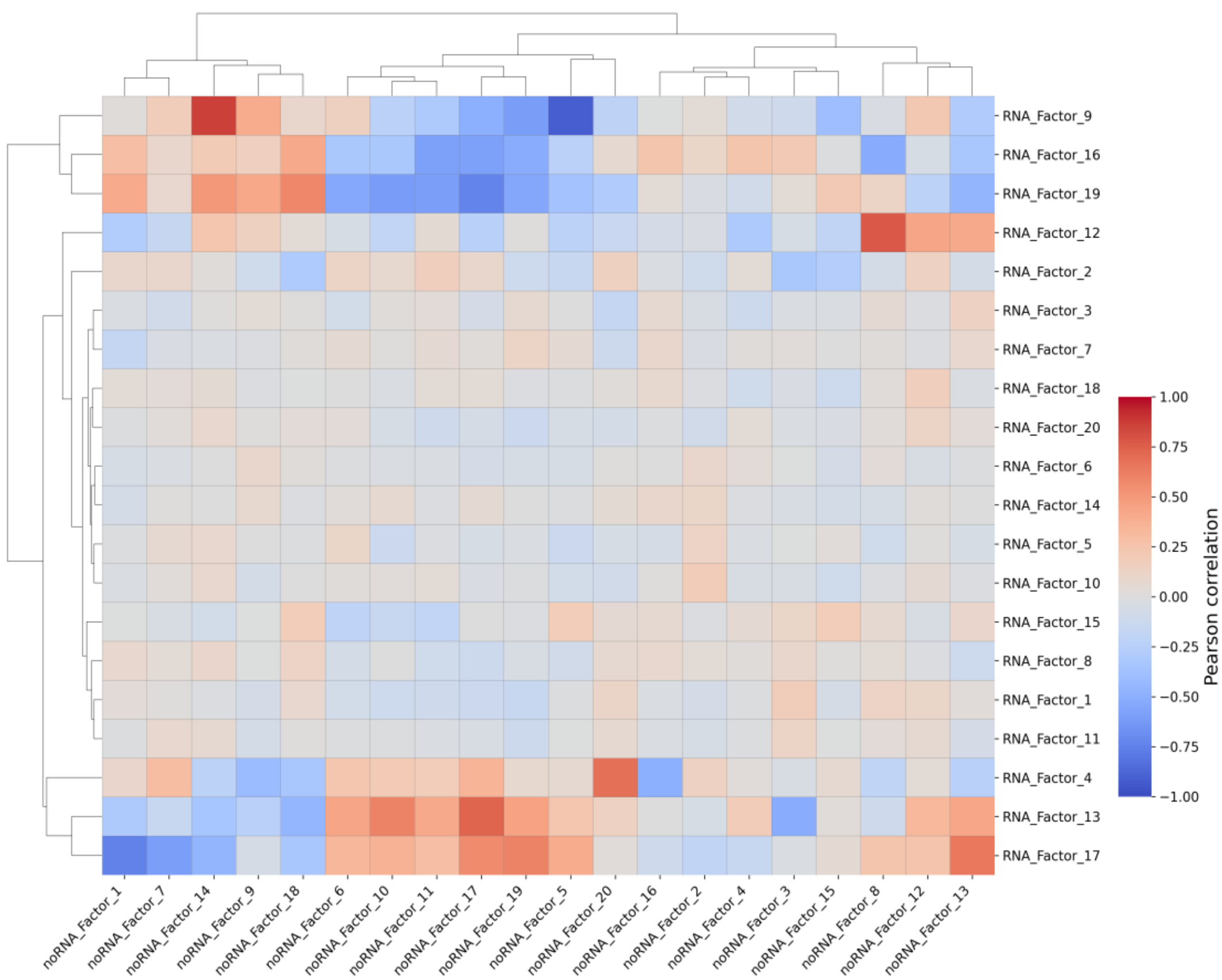

Appendix Figure S5 *Pairwise Pearson correlation of single-view MANTRA factors learnt based on the drug response tensor only and multi-view MANTRA factors based on the both drug response tensor and RNA-seq data. While most factors learnt by the single-view model are also inferred by the multi-view model, this does not hold vice-versa.*

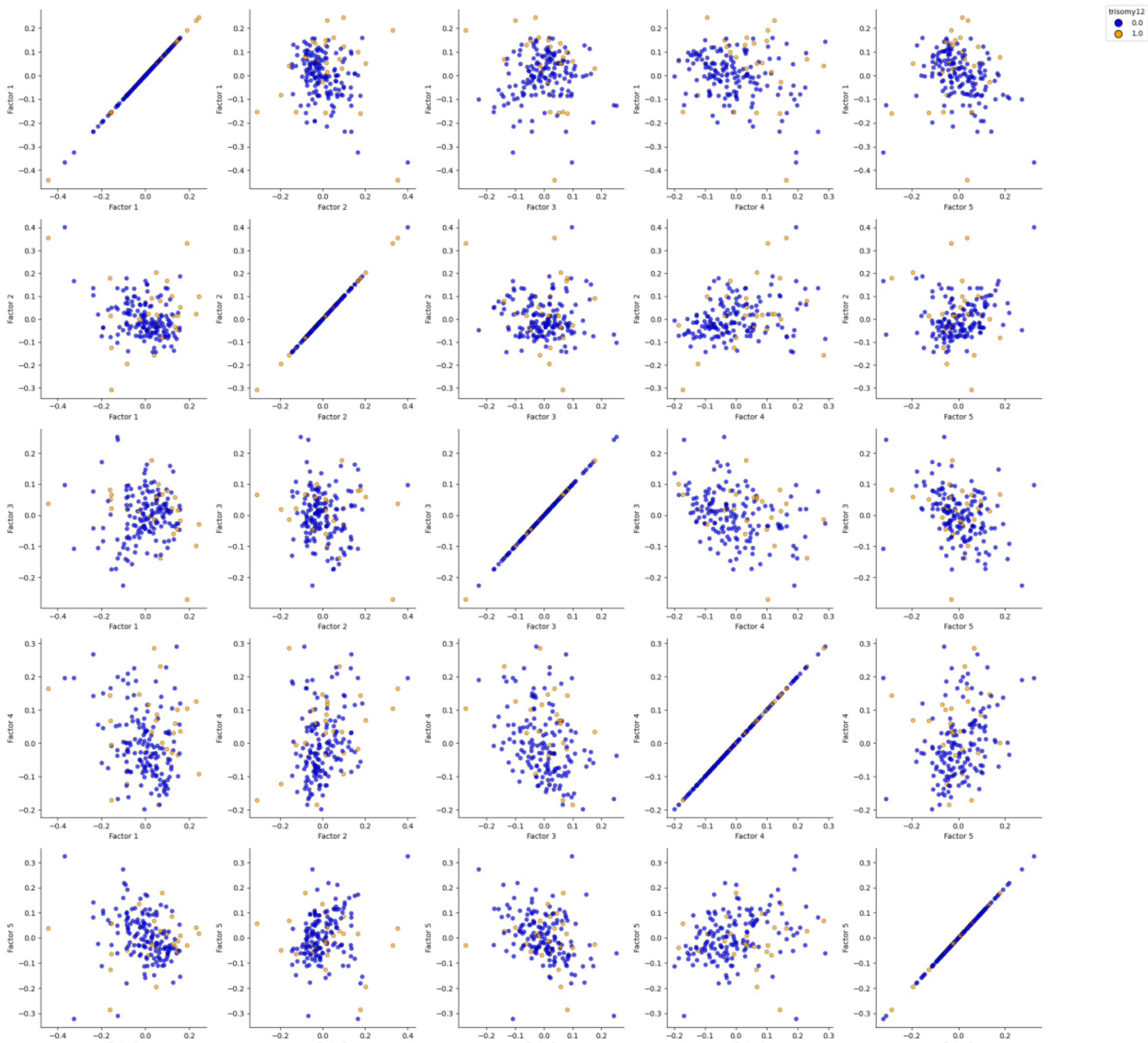

Appendix Figure S6 *Pairwise scatter plot for MOFA-FLEX annotated with Trisomy 12. MOFA-FLEX is not able to capture this covariate.*

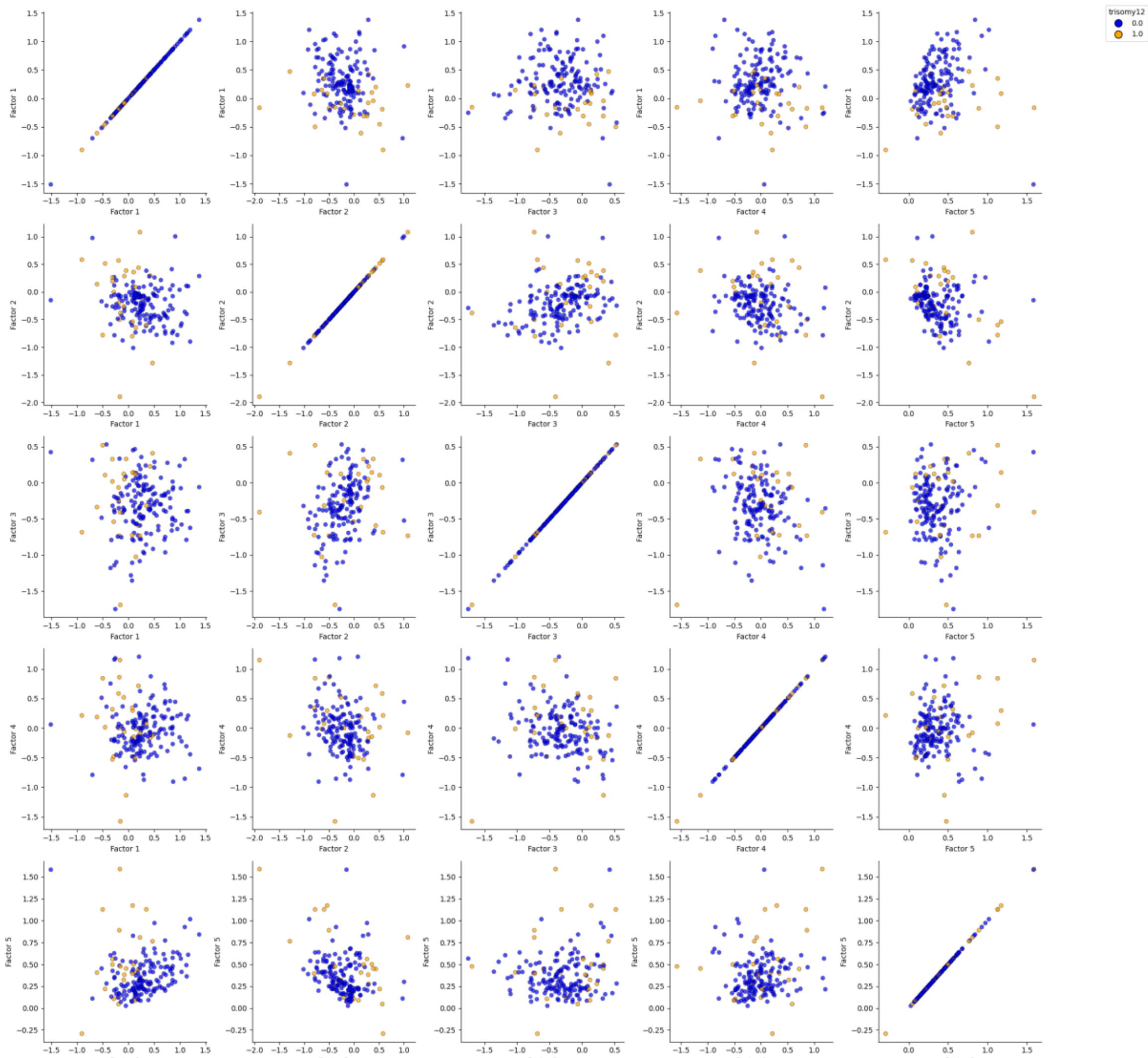

Appendix Figure S7 *Pairwise scatter plot for MANTRA annotated with Trisomy 12. Separation improves over MOFA-FLEX.*

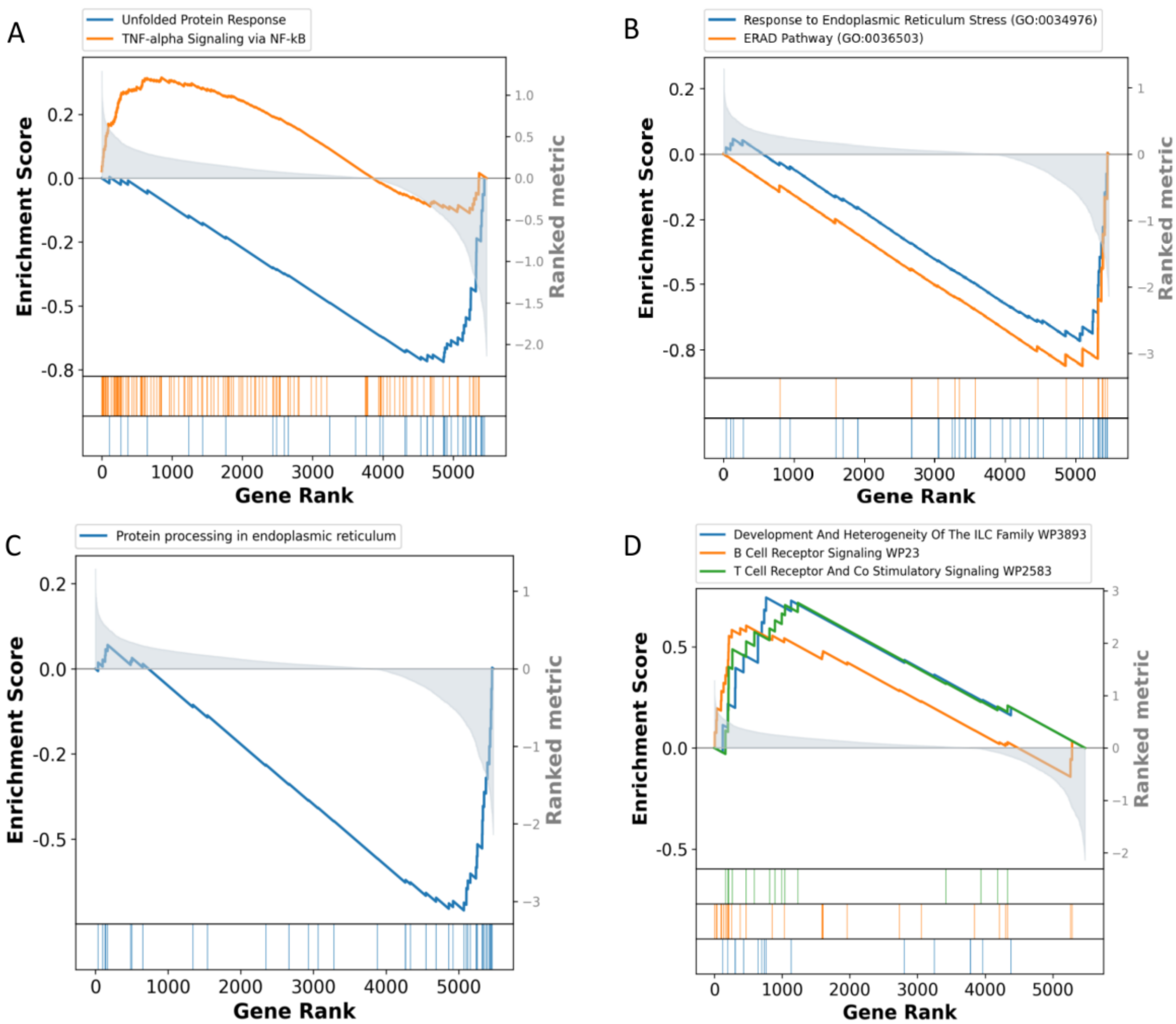

Appendix Figure S8 *GSEA for RNA Model Factor 5 in pDC cells. GSEA performed on factor loadings from RNA model Factor 5 for pDC cells identified pathways from four databases (FDR q-val < 0.05). (A) MSigDB Hallmark. Positive enrichment for TNF-alpha signaling via NF-kB (NES=1.76, FDR=0.018). Negative enrichment for unfolded protein response (NES=-1.90, FDR=0.001). (B) GO Biological Process. Negative enrichment for ER stress response (NES=-1.90, FDR=0.019) and ERAD pathway (NES=-1.84, FDR=0.045). (C) KEGG Pathways. Negative enrichment for protein processing in endoplasmic reticulum (NES=-1.91, FDR=0.003). (D) WikiPathways. Positive enrichment for immune signaling: ILC development (NES=2.05, FDR=0.013), B cell receptor signaling (NES=1.94, FDR=0.034), and T cell receptor signaling (NES=1.94, FDR=0.023). Results confirm concurrent immune activation and ER stress pathways in RNA-Factor 5. Positive NES indicates upregulation; negative NES indicates downregulation.*

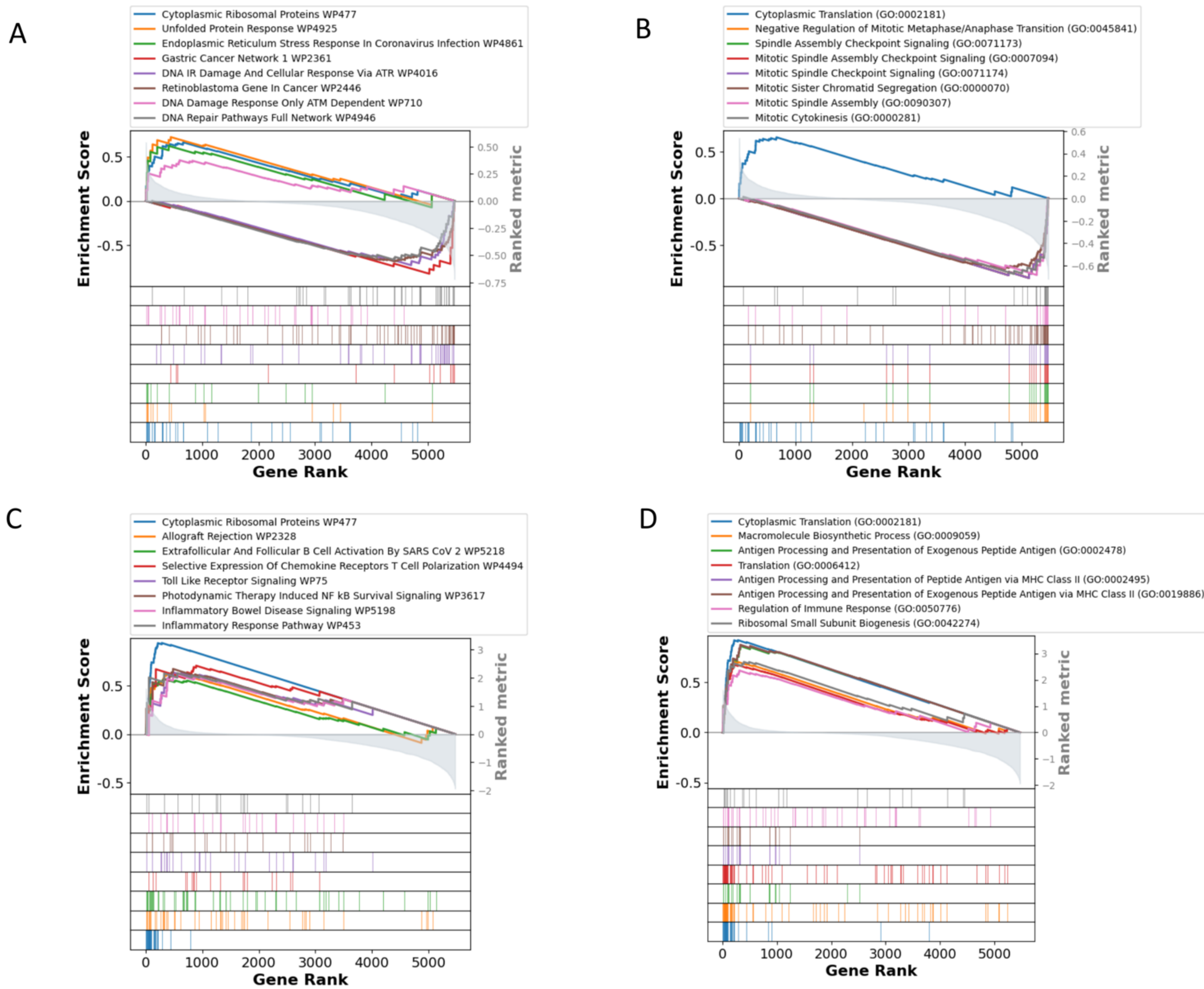

Appendix Figure S9 *Gene Set Enrichment Analysis (GSEA) reveals distinct biological processes associated with Factor 2 (RNA)/Factor 3 (Multiome) in Blasts. GSEA performed on factor loadings from (A–B) RNA model Factor 2 and (C–D) Multiome model Factor 3, both specific to Blasts. Top 8 pathways shown; full results include all significantly enriched pathways (FDR q-val < 0.05). (A) RNA Factor 2 – WikiPathways (8 of 8 shown). Positive enrichment for cytoplasmic ribosomal proteins (NES = 2.35, FDR < 0.001) and unfolded protein response (NES = 2.08, FDR = 0.016). Negative enrichment for cell cycle regulation (NES = -1.60, FDR = 0.037), DNA replication (NES = -1.60, FDR = 0.039), and DNA repair (NES = -1.61, FDR = 0.042). (B) RNA Factor 2 – GO Biological Process (8 of 39 shown). Cytoplasmic translation upregulated (NES = 2.32, FDR = 0.004). Mitotic processes downregulated: spindle organization (NES = -1.77, FDR = 0.002), cytokinesis (NES = -1.83, FDR < 0.001), and spindle checkpoint (NES = -1.88, FDR < 0.001). (C) Multiome Factor 3 – WikiPathways (8 of 11 shown). Strong enrichment for ribosomal proteins (NES = 3.19, FDR < 0.001), allograft rejection (NES = 2.26, FDR < 0.001), B cell activation (NES = 2.14, FDR < 0.001), Toll-like receptor signaling (NES = 2.11, FDR < 0.001), and inflammatory response (NES = 1.90, FDR < 0.001). (D) Multiome Factor 3 – GO Biological Process (8 of 21 shown). Positive enrichment for translation (NES = 3.18, FDR < 0.001), macromolecule biosynthesis (NES = 2.81, FDR < 0.001), antigen processing/presentation (NES = 2.76/2.64, FDR < 0.001), and immune response regulation (NES = 2.34, FDR < 0.001). Positive NES indicates upregulation; negative NES indicates downregulation.*

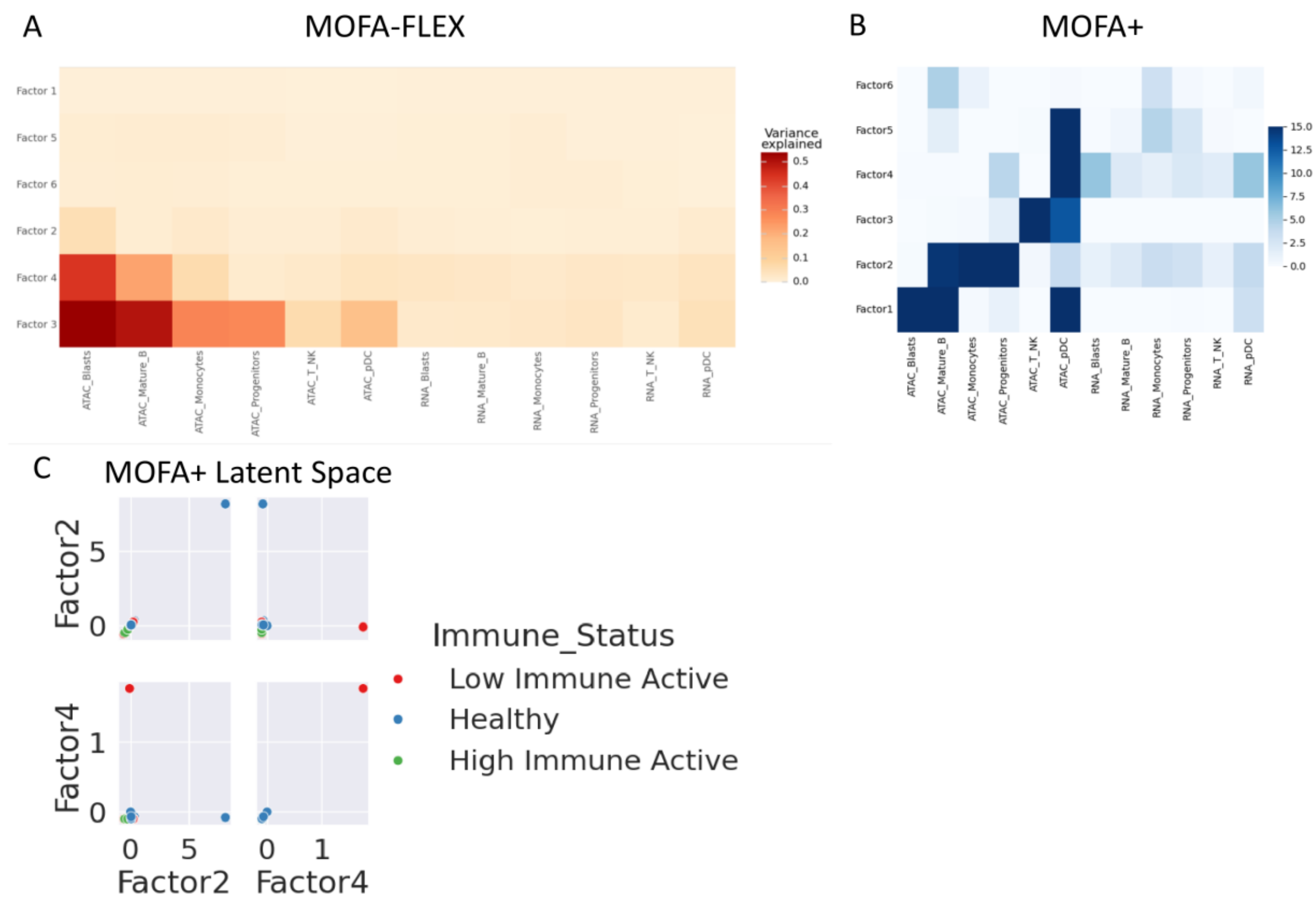

Appendix Figure S10 *MOFA+ and MOFA-FLEX trained on the Multiome data modelling tensor slices as views. None of the factors identified by MOFA-FLEX explains variance in the pDC-RNA view (A). The two factors identified by MOFA+ which do explain some variance in the pDC-RNA view (B) do not capture the immune high/low phenotype revealed by MANTRA (C).*

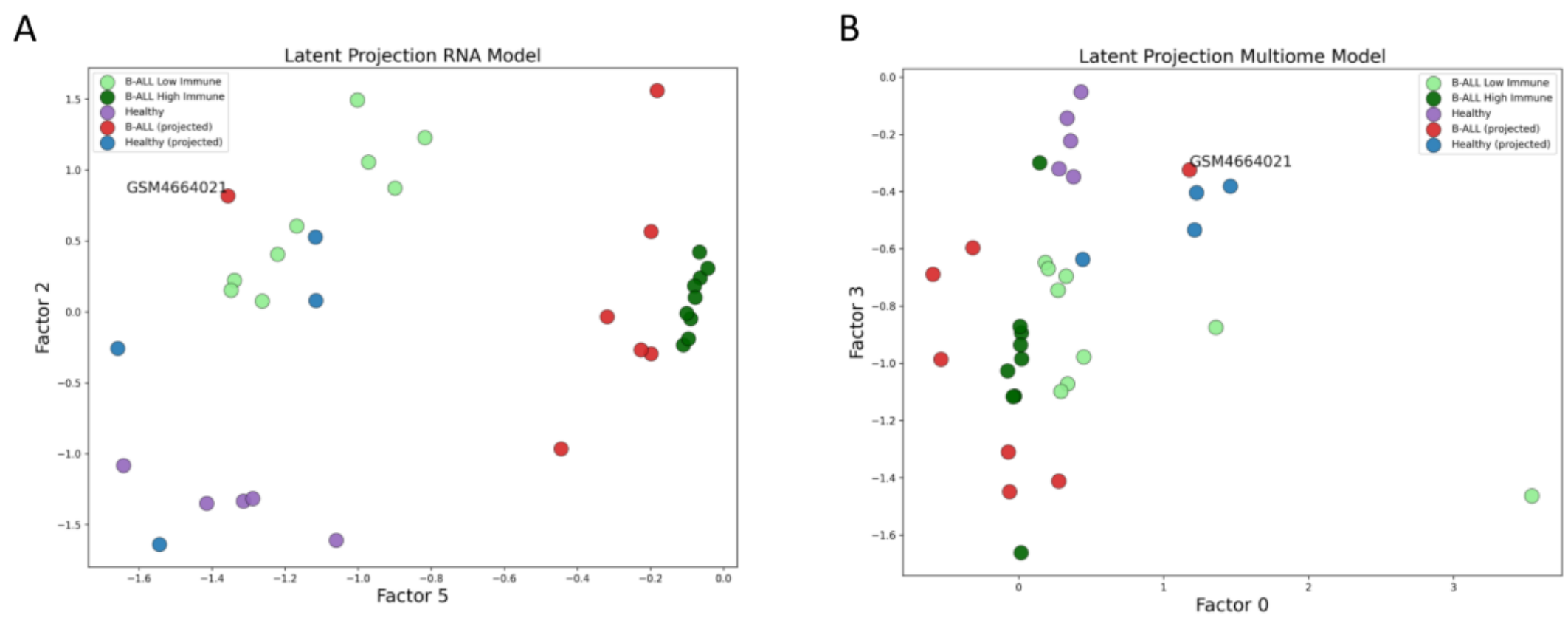

Appendix Figure S11 *Embeddings of MANTRA models trained on the RNA tensor of the discovery cohort only (A) and trained on the Multiome (B). Samples from the validation cohort were projected into this space by freezing the feature embeddings and cell-type embeddings. Healthy donors from the validation cohort cluster with healthy donors from the discovery cohort; patients form the validation cohort cluster largely with the high immune subtype, but notably the sample with the lowest immune activity in the MANTRA model trained directly on the validation cohort (GSM4664021) clusters with the low immune patients of the discovery cohort.*

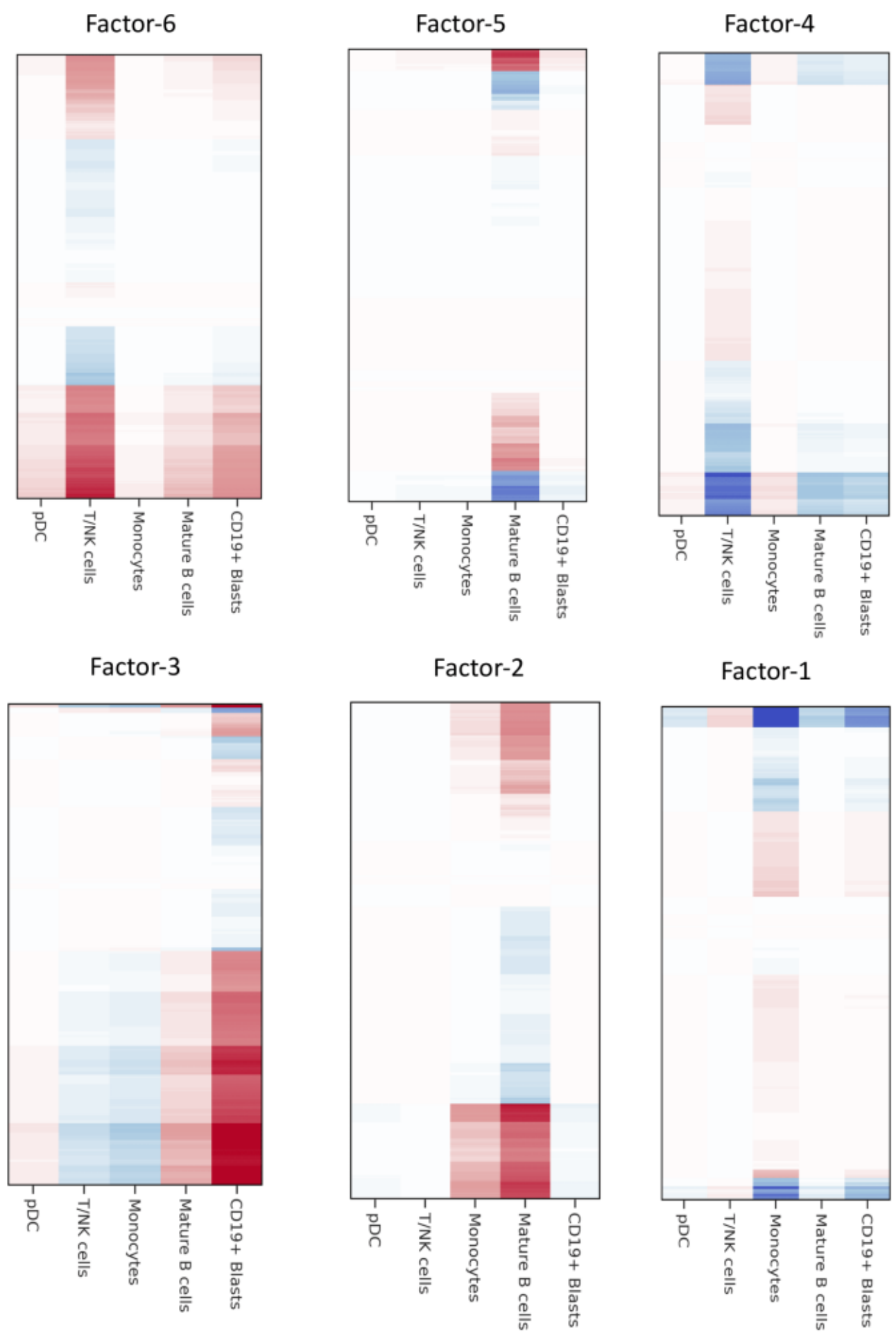

Appendix Figure S12 *Loadings of a MANTRA model trained on a cohort of T-ALL patients. None of the factors was active in pDCs, suggesting that the pDC axis of variation identified by MANTRA in B-ALL is specific to B-ALL.*
