# Supplementary material for: Interpretable multi-omics integration across mixed-order tensors with MANTRA
Source: Mol Syst Biol. 2026 Jun 18;22(9):1415–29. doi: 10.1038/s44320-026-00223-8 (PMC13538497; doi:10.1038/s44320-026-00223-8)
Supplement: Supplementary file 2 [file 44320_2026_223_MOESM2_ESM.pdf]

molecular systems biology

Interpretable multi-omics integration across mixed-order tensors with MANTRA

Kevin de Azevedo, Yusuf Oruc, and Florian Buettner

Corresponding author(s): Florian Buettner (florian.buettner@dkfz-heidelberg.de)

Review Timeline:

Submission Date:	15th Jul 25
Editorial Decision:	5th Sep 25
Revision Received:	1st Feb 26
Editorial Decision:	3rd Mar 26
Revision Received:	18th May 26
Accepted:	3rd Jun 26

Editor: Jingyi Hou

Transaction Report:

(Note: With the exception of the correction of typographical or spelling errors that could be a source of ambiguity, letters and reports are not edited. Depending on transfer agreements, referee reports obtained elsewhere may or may not be included in this compilation. Referee reports are anonymous unless the Referee chooses to sign their reports.)

5th Sep 2025

Manuscript Number: MSB-2025-13234
Title: Interpretable multi-omics integration across mixed-order tensors with MUSIC
Author: Kevin de Azevedo
Florian Büttner

Dear Florian,

Thank you for submitting your work to Molecular Systems Biology. First of all, I would like to apologize for the slow process, which was due to the delays in obtaining reviewers' report. We are still awaiting feedback from Reviewer #3. However, given the alignment in the recommendations of the other two reviewers, I have decided to proceed with a decision at this stage to avoid further delay. Should we receive additional comments from Reviewer #3, we will share them with you, and you may be asked to address any further issues raised.

As you will see from the reports below, the reviewers recognize the potential significance of your study. However, they also raise several concerns that must be addressed in a major revision. Given the clarity of the reviewers' recommendations, I will not reiterate all the points here. However, it is essential that all issues raised are addressed thoroughly in your revised manuscript. In particular, the shared concerns regarding benchmarking require careful attention, and we ask that you perform some level of validation for the patient stratification analysis, as suggested by Reviewer #2.

As you may already know, our editorial policy allows in principle a single round of major revision and it is therefore essential to provide responses to the reviewers' comments that are as complete as possible.

On a more editorial level, we would ask you to address the following issues:

- Please provide a .docx formatted version of the manuscript text (including legends for main figures, EV figures and tables). Please make sure that the changes are highlighted to be clearly visible.

- Please provide individual production quality figure files as .eps, .tif, .jpg (one file per figure).

-Please provide a .docx formatted letter INCLUDING the reviewers' reports and your detailed point-by-point responses to their comments. As part of the EMBO Press transparent editorial process, the point-by-point response is part of the Review Process File (RPF), which will be published alongside your paper.

-Please note that all corresponding authors are required to supply an ORCID ID for their name upon submission of a revised manuscript.

-We replaced Supplementary Information with Expanded View (EV) Figures and Tables that are collapsible/expandable online (see examples in http://msb.embopress.org/content/11/6/812). A maximum of 5 EV Figures can be typeset. EV Figures should be cited as 'Figure EV1, Figure EV2" etc... in the text and their respective legends should be included in the main text after the legends of regular figures.

Additional Tables/Datasets should be labeled and referred to as Table EV1, Dataset EV1, etc. Legends have to be provided in a separate tab in case of .xls files. Alternatively, the legend can be supplied as a separate text file (README) and zipped together with the Table/Dataset file.
For the figures and tables that you do NOT wish to display as Expanded View figures, they should be bundled together with their legends in a single PDF file called *Appendix*, which should start with a short Table of Content. Each legend should be below the corresponding Figure/Table in the Appendix. Appendix figures and tables should be referred to in the main text as: "Appendix Figure S1, Appendix Figure S2, Appendix Table S1" etc. See detailed instructions regarding expanded view here: https://www.embopress.org/page/journal/17444292/authorguide#expandedview.

-Before submitting your revision, primary datasets (and computer code, where appropriate) produced in this study need to be deposited in an appropriate public database (seehttp://msb.embopress.org/authorguide - dataavailability https://www.embopress.org/page/journal/17444292/authorguide#dataavailability).
Please remember to provide a reviewer password if the datasets are not yet public.
The accession numbers and database should be listed in a formal "Data Availability" section (placed after Materials & Method) that follows the model below (see also https://www.embopress.org/page/journal/17444292/authorguide#dataavailability).

Please note that the Data Availability Section is restricted to new primary data that are part of this study.

Data availability
The datasets (and computer code) produced in this study are available in the following databases:
- RNA-Seq data: Gene Expression Omnibus GSE46843 (https://www.ncbi.nlm.nih.gov/geo/query/acc.cgi?acc=GSE46843)
- [data type]: [name of the resource] [accession number/identifier/doi] ([URL or identifiers.org/DATABASE:ACCESSION])
*** Note - All links should resolve to a page where the data can be accessed. ***

- At EMBO Press we ask authors to provide source data for the main manuscript figures. You will receive a separate email with instructions for providing source data with your revised manuscript, including how to upload and organize the files.

Additional information on source data and instruction on how to label the files are available

- Our journal encourages inclusion of *data citations in the reference list
* to directly cite datasets that were re-used and obtained from public databases. Data citations in the article text are distinct from normal bibliographical citations and should directly link to the database records from which the data can be accessed. In the main text, data citations are formatted as follows: "Data ref: Smith et al, 2001". In the Reference list, data citations must be labeled with "[DATASET]". A data reference must provide the database name, accession number/identifiers and a resolvable link to the landing page from which the data can be accessed at the end of the reference. Further instructions are available at .

- We updated our journal's competing interests policy in January 2022 and request authors to consider both actual and perceived competing interests. Please review the policy https://www.embopress.org/competing-interests and update your competing interests if necessary.
Please use the heading "Disclosure statement and competing interests".

- All Materials and Methods need to be described in the main text using our 'Structured Methods' format. According to this format, the Methods section includes a Reagents and Tools Table (listing key reagents, experimental models, software and relevant equipment and including their sources and relevant identifiers) followed by a Methods and Protocols section describing the methods, ideally using a step-by-step protocol format. The aim is to facilitate adoption of the methodologies across labs. Please download and fill our Reagents and Tools Table template (.docx), which you can find in our author guidelines: https://www.embopress.org/page/journal/17444292/authorguide#structuredmethods.
When submitting your revised manuscript, please do not include the Reagents and Tools Table in the Methods section of the manuscript but upload it as a separate file choosing the file type "Reagent Table".

An example of a Method paper with Structured Methods can be found here:
https://www.embopress.org/doi/10.15252/msb.20178071.

-Regarding data quantification:
Please ensure to specify the name of the statistical test used to generate error bars and P values, the number (n) of independent experiments (please specify technical or biological replicates) underlying each data point and the test used to calculate p-values in each figure legend. Discussion of statistical methodology can be reported in the materials and methods section, but figure legends should contain a basic description of n, P and the test applied.
Graphs must include a description of the bars and the error bars (s.d., s.e.m.).
Please also include scale bars in all microscopy images.

- Please provide a "standfirst text" summarizing the study in one or two sentences (approximately 250 characters, including space), three to four "bullet points" highlighting the main findings and a "synopsis image" (550px width and 400-600 px height, PNG format) to highlight the paper on our homepage.
Here are a couple of examples:
https://www.embopress.org/doi/10.15252/msb.20199356
https://www.embopress.org/doi/10.15252/msb.20209475
https://www.embopress.org/doi/10.15252/msb.209495

When you resubmit your manuscript, please download our CHECKLIST (https://www.embopress.org/pb-assets/embo-site/EMBO%20Press%20Author%20Checklist-1642513524327.xlsx) and include the completed form in your submission. *Please note* that the Author Checklist will be published alongside the paper as part of the transparent process (https://www.embopress.org/page/journal/17444292/authorguide#transparentprocess).

If you feel you can satisfactorily deal with these points and those listed by the referees, you may wish to submit a revised version of your manuscript. Please attach a covering letter giving details of the way in which you have handled each of the points raised by the referees. A revised manuscript will be once again subject to review and you probably understand that we can give you no guarantee at this stage that the eventual outcome will be favorable.

I look forward to receiving your revised manuscript soon.

Kind regards,
Jingyi

Jingyi Hou, PhD
Senior Editor
Molecular Systems Biology

We realize that it is difficult to revise to a specific deadline. In the interest of protecting the conceptual advance provided by the work, we recommend a revision within 3 months (4th Dec 2025). Please discuss the revision progress ahead of this time with the editor if you require more time to complete the revisions. Use the link below to submit your revision:

https://msb.msubmit.net/cgi-bin/main.plex

As a matter of course, please make sure that you have correctly followed the instructions for authors as given on the submission website.

*** PLEASE NOTE *** As part of the EMBO Press transparent editorial process initiative (see our Editorial at https://dx.doi.org/10.1038/msb.2010.72), Molecular Systems Biology publishes online a Review Process File with each accepted manuscripts. This file will be published in conjunction with your paper and will include the anonymous referee reports, your point-by-point response and all pertinent correspondence relating to the manuscript. If you do NOT want this File to be published, please inform the editorial office at contact@molsystbiol.org within 14 days upon receipt of the present letter.

Reviewer #1:

The authors represent MUSIC for multi-view tensor data analysis with differing dimensions. in a clearly written manuscript and demonstration of usage which previous multi-omits factor analysis methods would not provide. Here a few comments:

- Description of figure 2 is inadequate. The plot shows lower RMSE for MUSIC especially as the number of samples increases, but no explanation of this behavior in the text. Also some details about the parameters needed to comprehend the plot, e.g. how many factors were considered for data reconstruction in each case?

- Why Figure 3 is labeled "patients loading" whereas the plot shows a different loading (color intensity) for each patient-drug element of the matrix?

- Furthermore, is it OK that the loadings are totally different with versus without considering the RNA-seq data? Wouldn't it mean that results are unreliable because by considering any new modality of information the previous results would become totally irrelevant? How do you interpret such a big instability?

- "In this Multiview setting, MUSIC uncovered a clear separation of patients based on their IGHV mutation status and Trisomy 12 status, two well-characterized prognostic markers in CLL (Fig. ??)." Figure number missing, if Figure 4b is meant, the separation is not clear in fact. Could you quantify a good versus bad separation?

- In the model specification section parameters T (capital) and m (small) are not described.

- In the same section, in equation 1, after summation over r indices, the variable should not remain on the Left Hand Side in superscribe of y.

- Why do the authors choose to compare with MOFA rather than its more advanced developments such as MAFISTO?

Reviewer #2:

This manuscript de Azevedo and Buettner introduces MUSIC (MUltiview BayeSIan Tensor DeComposition), a probabilistic framework for integrating multi-modal datasets represented as tensors of varying orders. This addresses a key challenge in current multi-omics integration approaches by jointly modeling higher-order and mixed-order data structures - entities which

become more and more prevalent in recent multi-omics studies. The method uses a Bayesian approach using with structured sparsity priors to handle missing data and allows for interpretability and scalability.

The authors apply their framework to simulated data and apply it to two biomedically relevant data sets: a drug response and RNA-seq data study in Chronic Lymphocytic Leukemia (CLL), and to scRNA-seq and scATAC-seq analyses in pediatric Acute Lymphoblastic Leukemia (ALL). Their MUSIC tool identified biologically and clinically relevant substructures that were not previously identified by conventional matrix-based methods or single tensor decompositions.

While the manuscript addresses a timely challenge of heterogeneous multiomics integration, which is relevant for many systematic molecular biomedical studies, and is developing an innovative approach that can be of high interests for bioinformaticians, the manuscript appears to be in a still pre-mature state for MSB.

Major points:

1. Overall, the ms lacks a more rigorous benchmarking and crucial details. While efforts have been made to perform comparison for the synthetic data with PARAFAC, the details of the data and potential dependence on the data size were not addressed. Similarly, the analysis of the 2 biomedical applications remains on a rather superficial level both on the benchmarking as well as for the biomedical interpretation. For benchmarking, the authors should not only consider MOFA but also more recent developments such totalVI, MOFA+, scITD, MONFIT) and also use additional measures like ARI, silhouette score, or explained variance. Furthermore, a benchmarking should also include dependency on MUSIC model parameter choices, structured sparsity and linearity which should be also addressed in a corresponding limitation analysis.

2. The overserved improved classification of patients looks indeed promising but so far it is more an interesting observation and would require some validation either by targeted experiments or independent data. In this respect, a more detailed biomedical description as well as discussion is lacking like for the IGHV and Trisomy 12 status in the disease context.

3. The manuscript could also be better organised. For instance, the Methods section seems to be misplaced, and some figures are not referred to or are not included in the general story (see also diverse comments on minor points below). Some figures require also more explanations and should be discussed in more detail. Overall, the ms gives the impression that the submission was rushed before summer break.

Minor points:

• Typo in introduction: "unsupervised modelling approaches are can reveal..." → remove "are".
• Typo in first sentence of results and first sentence in 2.1: space between "MUSIC" and the "," should be removed. This also holds for "MUSIC 's" ... so probably the latex shortcut must be modified.
• The synthetic data used for performance test should be better detailed (and also mentioned in the Results text and not only in the Methods? What size were the tensors? How does the size affect the comparison?
• Fig. 3 seems to be referred as Fig. 4 in the text? Colour schemes for the column and raw colours are not defined. Maybe worth to put a label to the colour bar? The claim of the improved patient embedding should be better quantified by at least giving the cluster composition. The meaning for Fig. 4 remains unclear?
• Missing or broken figure ref in "In this multi- view setting, MUSIC uncovered a clear separation of patients based on their IGHV mutation status and Trisomy 12 status, two well-characterized prognostic markers in CLL (Fig. ??)." on page 6.
• Typo in caption of Fig. 5 "We can see than MOFA is not able to disentangle ... " - change "than" into "that".
• Supplementary Figures should be referred to as Supplementary figures and not consecutive figs.
• For me the separation between Healthy and ALL patients in Fig. 6 (a) seems to be driven by Factor 3 and not Factor 0? More explanation needed here as well as for the patterns in Fig. 6b.
• Caption of Fig. 7 seems to be incomplete.
• "What do the authors mean with "Special cases of MUSIC are group factor analysis as implemented in MOFA ..."?
• "Space" issue at the end of page 6 after "(Fig. 6)" and "."
• Use "\times" instead of "x".
• Methods seems to be a bit deplaced ... should be wither before results or after Discussion.
• Choices for alpha and betha for the inverse Gamma distribution should be commented.

Overall, the manuscript presents a compelling and original framework for multi-omics integration with mixed ranked tensors. However, the lack of some details, comprehensive benchmarking and critical discussion on the methodology and the biological findings give the impression that the submission was rushed. Hence, in its current form, the ms doesn't reach the standard of MSB.

We thank the reviewers for their constructive comments.
We respond to all comments below in a point-by-point manner, with our response highlighted in blue and have revised the manuscript accordingly (changes in the manuscript are highlighted in red).
In particular, we have substantially extended our synthetic benchmark, added quantitative comparisons to state-of-the-art baselines and performed a validation study on an external patient cohort to confirm our findings on the pediatric B-ALL patient cohort.

To avoid confusion with the existing MUSIC (MUlti-Subject SIngle Cell deconvolution) tool, we have also decided to rename MUSIC to MANTRA (Multi-view ANalysis with Tensor and matRix Alignment) and refer to our tool only as MANTRA in the revised manuscript as well as in this point-by-point response. We apologize for any inconvenience this may cause during the reviewing process.

Response to Reviewer #1

Comment: Description of figure 2 is inadequate. The plot shows lower RMSE for MUSIC especially as the number of samples increases, but no explanation of this behavior in the text. Also some details about the parameters needed to comprehend the plot, e.g. how many factors were considered for data reconstruction in each case?
Response: We have now substantially extended our synthetic benchmark to better characterize the behaviour of MANTRA across multiple relevant data characteristics and modelling choices. In particular, we now systematically vary (i) the number of latent factors (default:20), (ii) number of samples (default:20), (iii) sparsity in the data (default:0.1), (iv) signal-to-noise ratio (default:10 dB), (v) n_features (default:5000) and (vii) random noise (default = 1). Sparsity was determined by the following observations in real-world RNA tensor sparsity (%12 in the ALL data) and ATAC tensor sparsity (32% in the ALL data). Additionally we have run an additional experiment to mimic real-world applications where it is unclear what the true rank of the data is. In this experiment, we assess how robust MANTRA and Parafac are against mis-specified ranks. To disentangle the effect of these individual factors, we chose a default (given in parenthesis) and varied one factor at a time. This revealed that MANTRA has comparable performance to Parafac in the high SNR/high sample ratio and a substantially improved reconstruction error in the noisy data regime (low SNR,low sample_size, high std and high sparsity), mimicking real-world datasets with moderate-to-low SNR and moderate-to-high SNR (Fig-2). In addition, due to its sparsity priors it is substantially more robust against rank mis-specification than Parafac as the structured sparsity prior encourages the model to switch off un-needed factors (i.e. drive the factor scores to 0).
We have now added a detailed description of this benchmark to the Methods and Results section.

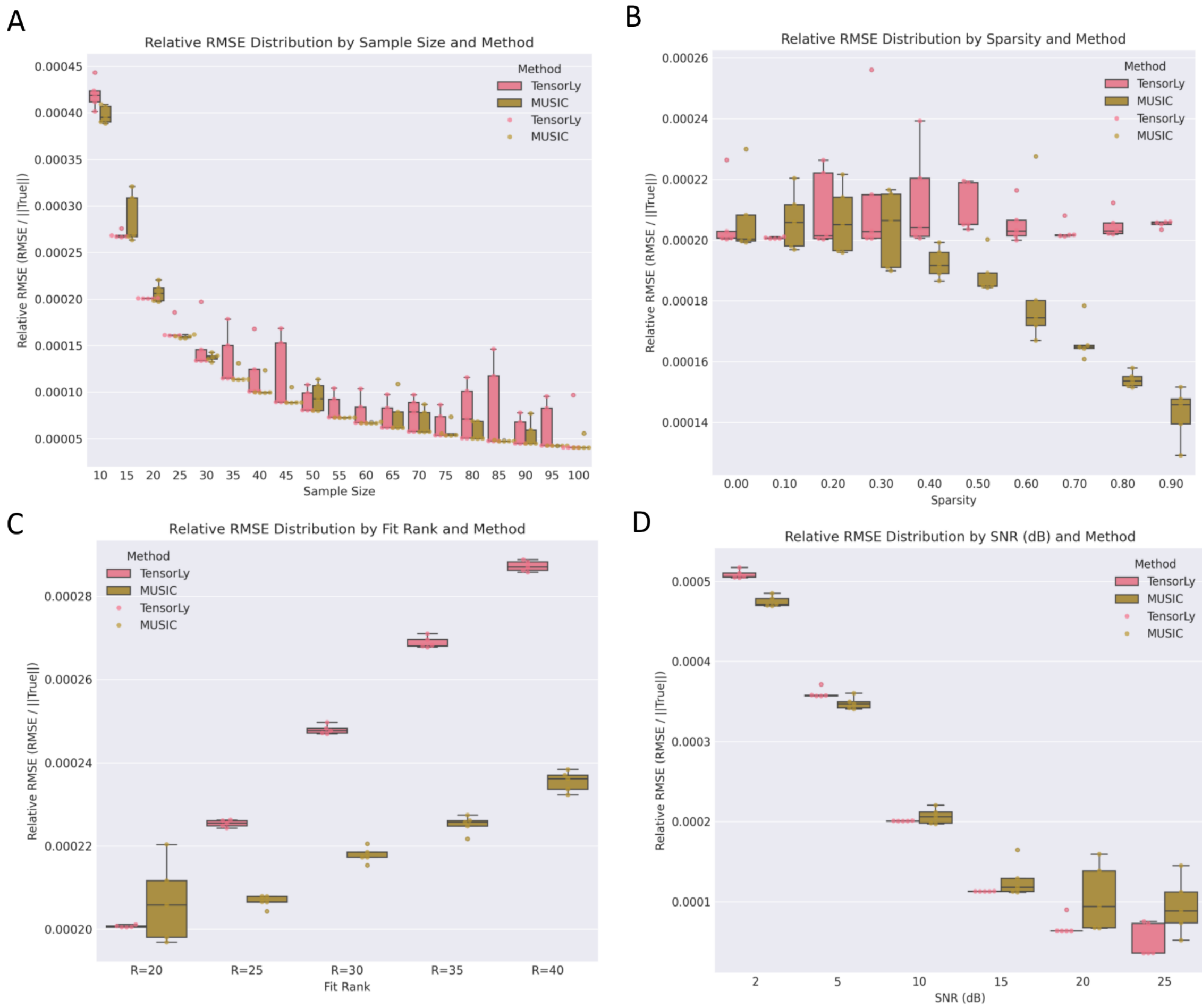

Fig. 2: Relative RMSE across typical data characteristics and modelling choices for Tensorly's Parafac method and MANTRA. Both methods are benchmarked for varying sample size (A), sparsity levels (B), rank (C) and signal-to-noise-ratio (D). RMSE was computed relative to the norm of the simulated (true) tensor to aid interpretability.

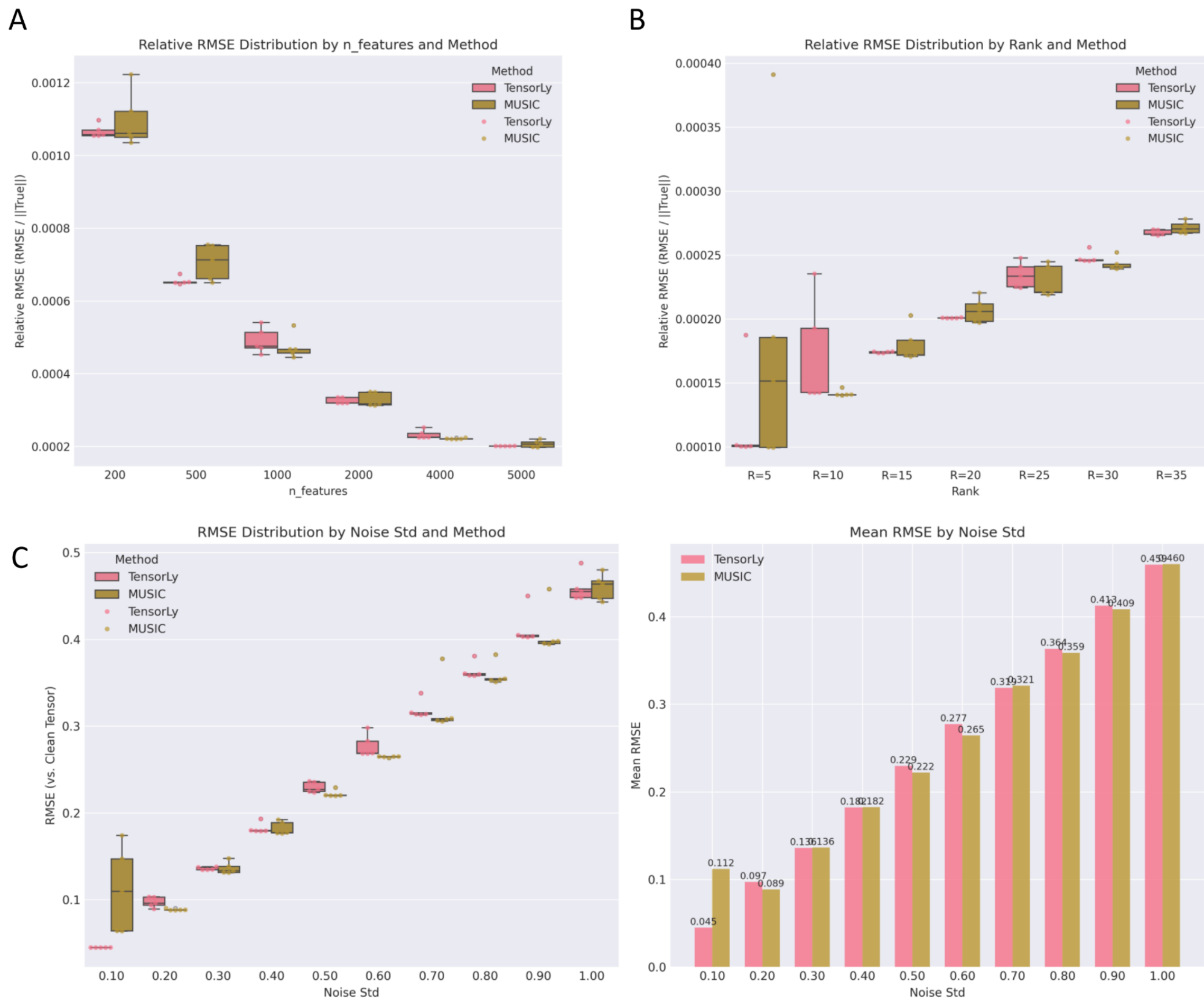

Supplementary Figure S1: Relative RMSE for varying numbers of features(A) and rank for simulated data (B) and for random noise (C).

Comment: Why Figure 3 is labeled "patients loading" whereas the plot shows a different loading (color intensity) for each patient-drug element of the matrix?
Response: This label was indeed misleading. We have now clarified that this figure shows the patient-drug loadings, computed as the outer product of the patient factor matrix (samples x factors) and the drug factor matrix (drug x factors) resulting in the (drugs x patients) loading matrix in the figure.

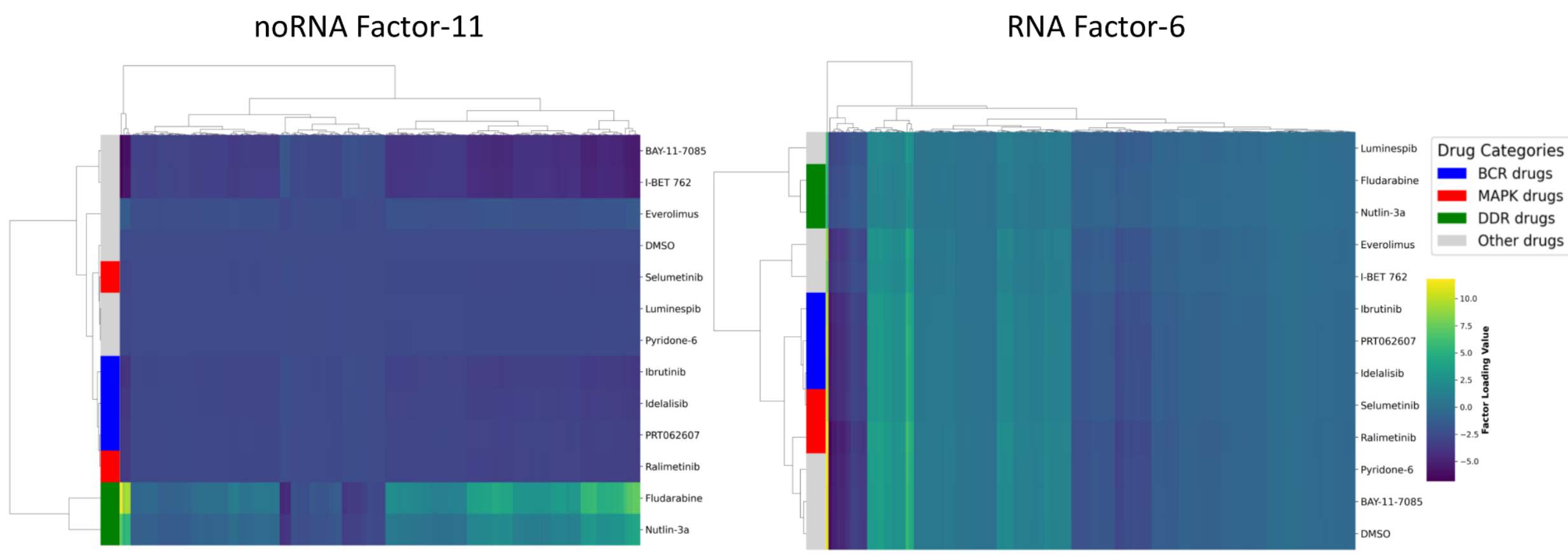

Fig. 3: Heatmap of the factor loadings for patients relative to drugs for a single-view MANTRA model trained on the drug viability tensor only (left) and a multi-view MANTRA model trained on the drug viability tensor as well as the RNA-seq matrix (right). Factor 11 and Factor 6 were the most discriminative factors with respect to drug category for both MANTRA models respectively. Integrating RNA-seq data further improved learnt representations in terms of discriminating drugs. The x-axis represents the patients,the y-axis represents drugs.

During the revision, we noticed that in the initially submitted manuscript, we had wrongly labelled two non-BCR drugs as BCR. We have corrected this and now explicitly show BCR, MAPK and DDR for the factors that were most discriminative wrt those categories. We apologise for this mistake.

Comment: Furthermore, is it OK that the loadings are totally different with versus without considering the RNA-seq data? Wouldn't it mean that results are unreliable because by considering any new modality of information the previous results would become totally irrelevant? How do you interpret such a big instability?
Response: It would indeed be unexpected for the factors to be uncorrelated when comparing with/without RNA-seq data. The mismatch between the factors is a result from the invariance property of factor analysis: factor 1 in the model with RNA does not necessarily match factor 1 in the model without RNA. We have now computed a cross-correlation plot, where we compare pair-wise correlation coefficients between factors of the models run with/without RNA. This

revealed that indeed Factor 11 in the noRNA model was strongly correlated to factor 19 in the RNA model (Pearson's r=-0.6). Importantly, this did not hold vice-versa and Factor 6 in the RNA model was only weakly correlated to the factors learnt without RNA, reflecting that the model was indeed able to learn unique representations in the multi-modal setup.

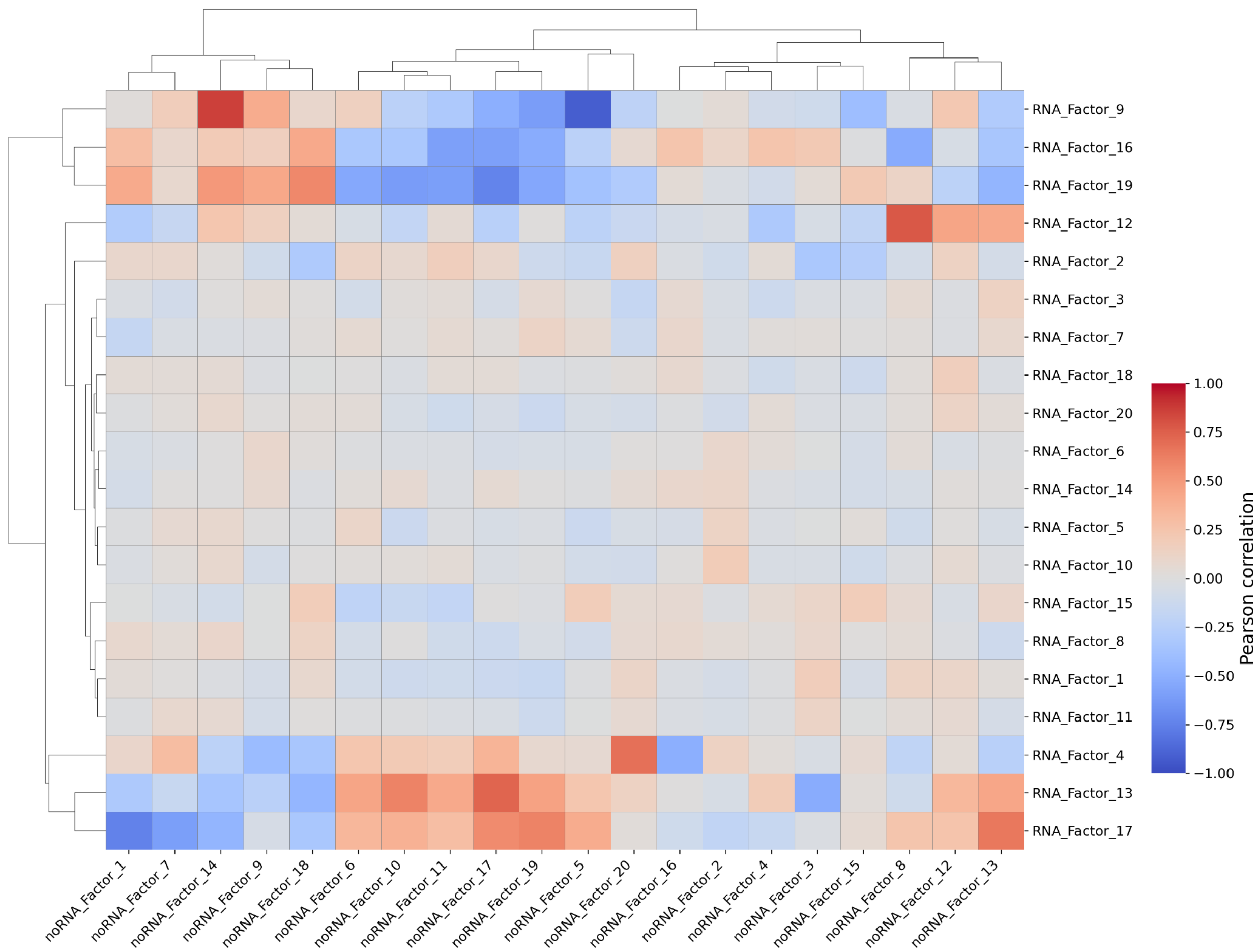

Supplementary Figure S2: Pairwise Pearson correlation of single-view MANTRA factors learnt based on the drug response tensor only and multi-view MANTRA factors based on the both drug response tensor and RNA-seq data. While most factors learnt by the single-view model are also inferred by the multi-viewmodel, this does not hold vice-versa.

Comment: "In this Multiview setting, MUSIC uncovered a clear separation of patients based on their IGHV mutation status and Trisomy 12 status, two well-characterized prognostic markers in CLL (Fig. ??)." Figure number missing, if Figure 4b is meant, the separation is not clear in fact. Could you quantify a good versus bad separation?

Response: We have now included a quantitative comparison to other methods (MOFA+, MOFA-FELX) using ARI and NMI on the learnt latent space (Fig. 4). This confirmed that (i) MANTRA was able to learn better representations (in terms of clustering performance) compared to multi-view integration methods and that (ii) the MANTRA model with RNA learnt quantifiably better representations than the model without RNA.

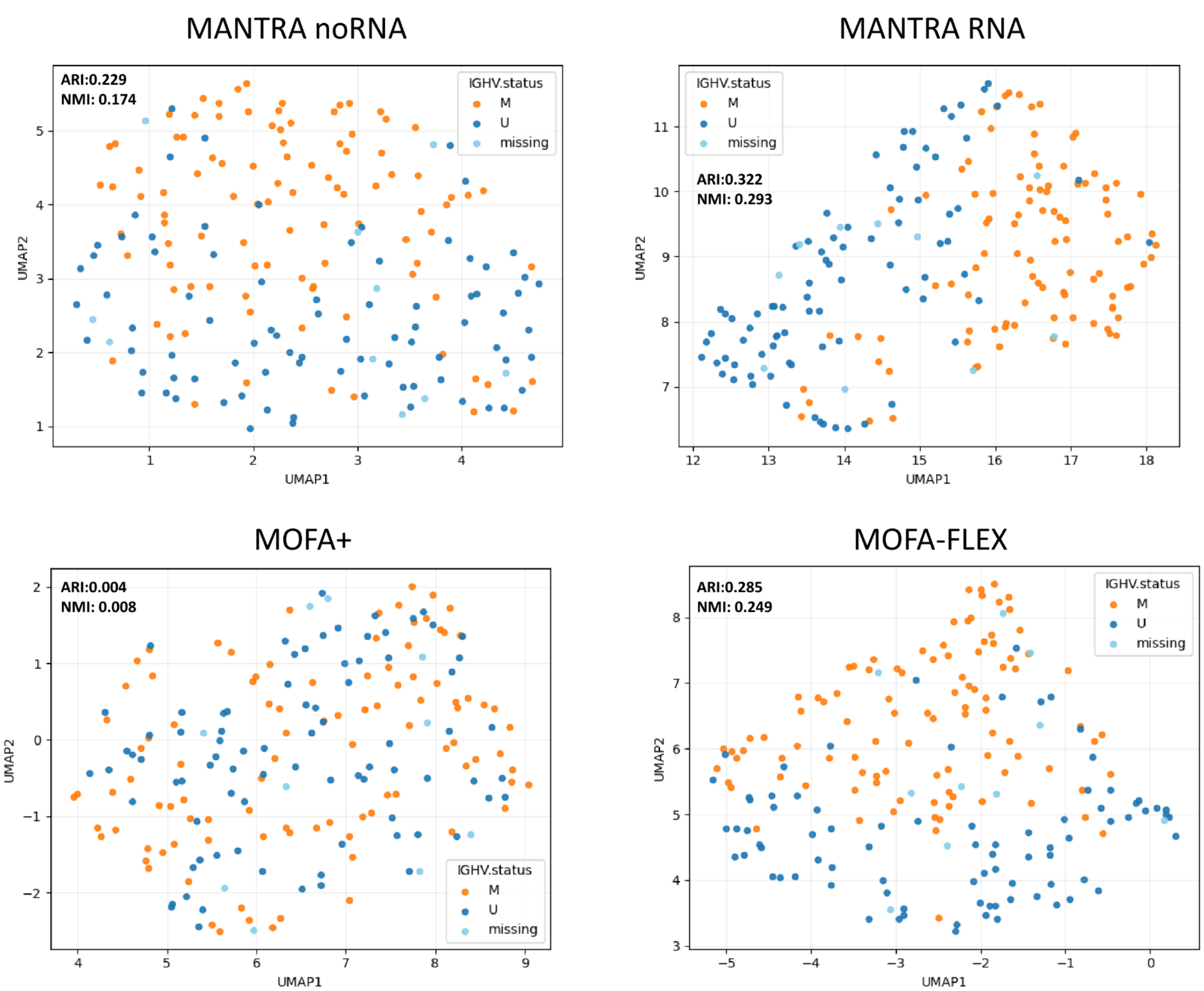

Fig. 4: UMAP of patient embeddings learned by MANTRA (single-view, top left), MANTRA (multiview, top right), MOFA+ (bottom left) and MOFA-FLEX (bottom right). Both MOFA+ and MOFA-FLEX were trained based on all slices of the drug viability tensor as well as the RNA-seq view. Adjusted Rand Index (ARI) and Normalized Mutual Information (NMI) with respect to Leiden clusters were computed to quantify how well individual methods could separate patients grouped by IGHV status.

We apologize for the broken figure references and have corrected these.

Comment: In the model specification section parameters T (capital) and m (small) are not described.
Response: We have extended the section specifying the model and explain in more that m corresponds to the index specifying the modality/data view. Subscripted T is used as an

identifier for a tensor, when referring to the subset of rank-3 tensors in the collection of input tensors.

Comment: In the same section, in equation 1, after summation over r indices, the variable should not remain on the Left Hand Side in superscribe of y.
Response: y on the LHS is indeed independent of r and superscripted with m_T and m_M, corresponding to the m_Tth rank-3 and m_Mth rank-2 tensor in the collection of input tensors.

Comment: Why do the authors choose to compare with MOFA rather than its more advanced developments such as MAFISTO?
Response: We have not compared MANTRA to MEFISTO as the latter tool was designed to account for spatio-temporal data via a GP prior on the factor scores. To add additional comparisons, we now benchmark MANTRA to MOFA-FLEX, a recently released versatile framework for factor-analysis based modelling of multi-omics data [1] and scITD, a tensor-factorisation tool designed for patient derived single-cell RNA-seq data. MANTRA consistently outperformed MOFA-FLEX on a quantitative evaluation of the Cytokine+RNA data. Neither MOFA-FLEX nor scITD were able to recover the patient stratification identified by MANTRA, that we have now validated in an external, independent cohort of patients (also see the response to Reviewer 2 on validation). To facilitate direct comparison to scITD, we also ran MANTRA on the RNA tensor alone. We have included all new figures below, with more detailed descriptions in the revised paper, highlighted in red.

[1] Qoku, A., Rohbeck, M., Walter, F. C., Kats, I., Stegle, O., & Buettner, F. (2025). MOFA-FLEX: A Factor Model Framework for Integrating Omics Data with Prior Knowledge. *bioRxiv*, 2025-11.

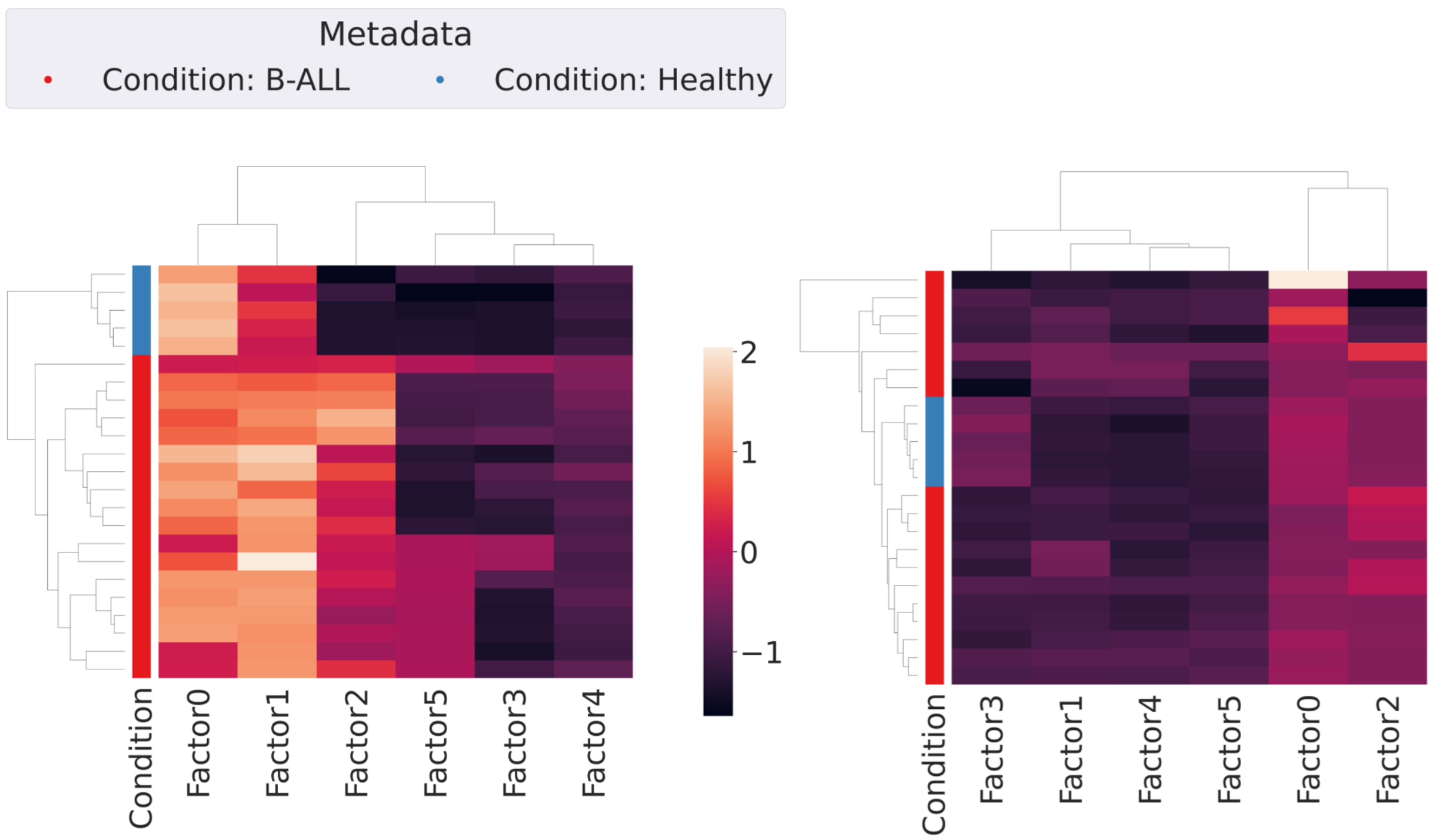

Fig. 5: Factor scores for a single-view MANTRA model learnt on the RNA-seq tensor only (left) and a multi-view MANTRA models integrating RNA-seq and ATAC-seq data (right). Both models clearly separate healthy donors from ALL patients and reveal heterogeneity between patients.

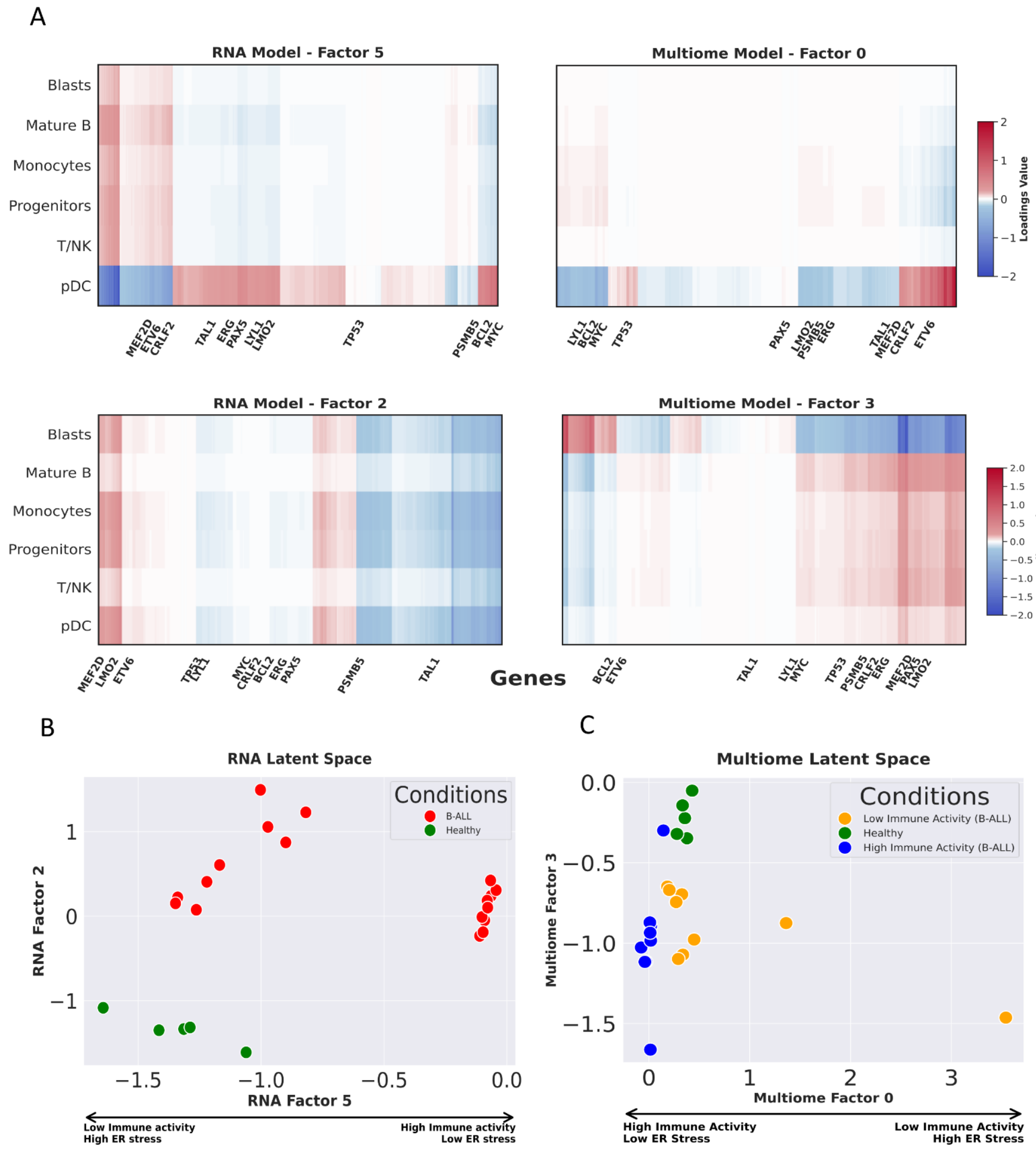

Fig. 7: Characterization of the MANTRA factors explaining variation between healthy donors and patients (Factor 2/RNA and Factor 3/Multiome) as well as factors delineating two B-ALL subpopulations (Factor 5/RNA and Factor 0/Multiome). A) Cell-type loading plots for the RNA model (left) show that Factor 5 is primarily active in pDCs, whereas Factor 2 explains variation in most cell types. Cell-type loading plots for the Multiome model (right) show patterns that are consistent with the RNA model, with cell-type attributions more focussed on individual cell-types than for the RNA model. B) Scatter plots separate healthy donors from B-ALL patients and reveal a B-ALL sub-population characterised by high immune activity/low ER stress. This subpopulation was consistently found for MANTRA models trained on RNA (left) and Multiome (right). Patients from the high immune activity/low ER stress cluster identified in the RNA latent space (right cluster) are shown in blue in the Multiome latent space to illustrate the consistency

between the embeddings. Similarly, patients from the low immune activity/high ER stress cluster identified in the RNA latent space (left cluster) are shown in yellow in the Multiome latent space.

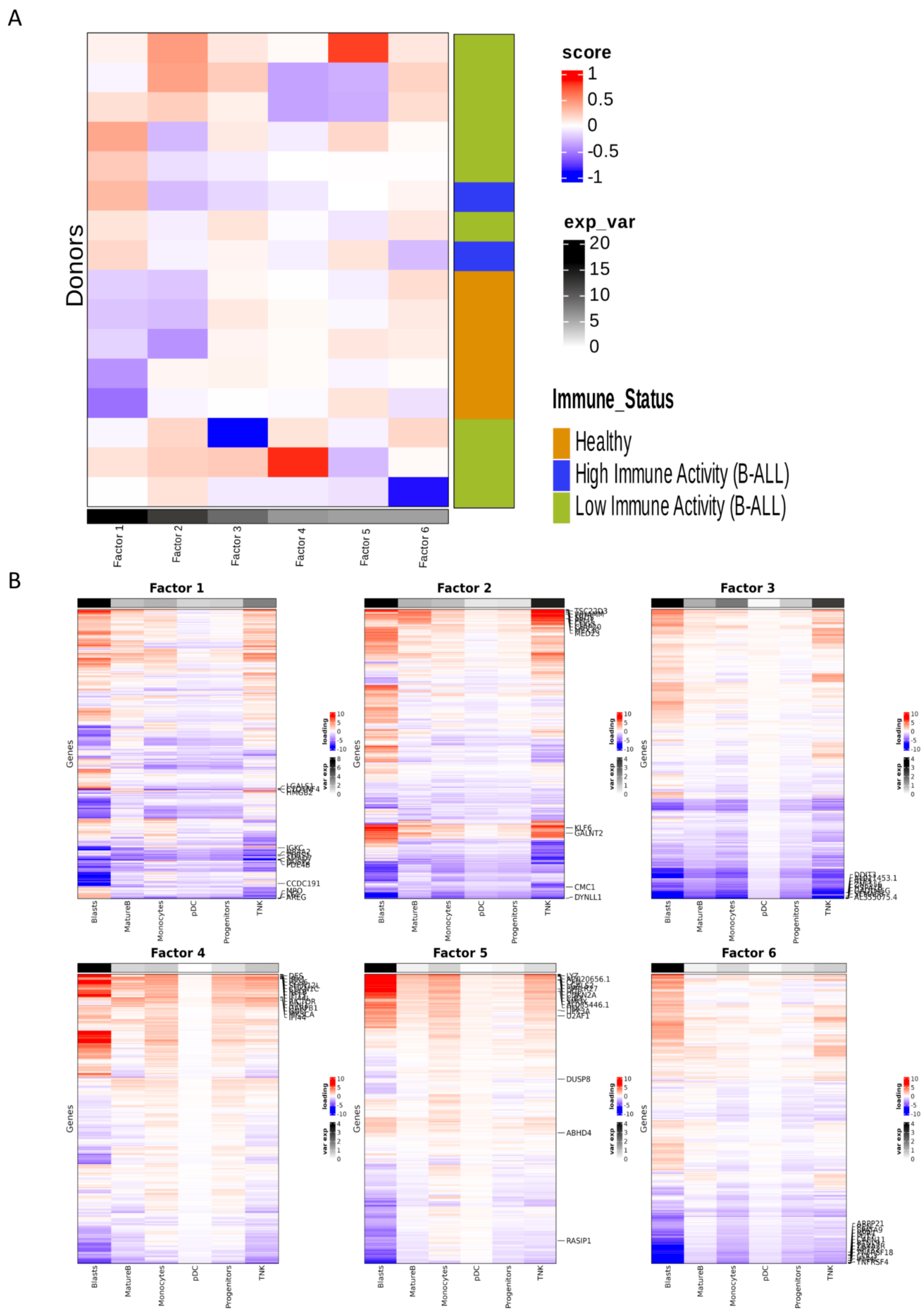

Fig. 6: scITD results on RNA-seq data. A) Donor factor scores. scITD was able to separate healthy from ALL patients (via Factor 1), but could not recover the additional structured variation between patients revealed by MANTRA . B) Cell-type-Gene factor loadings learnt by scITD mainly captured variation driven by blasts and did not identify the pDC-driven factor found by MANTRA.

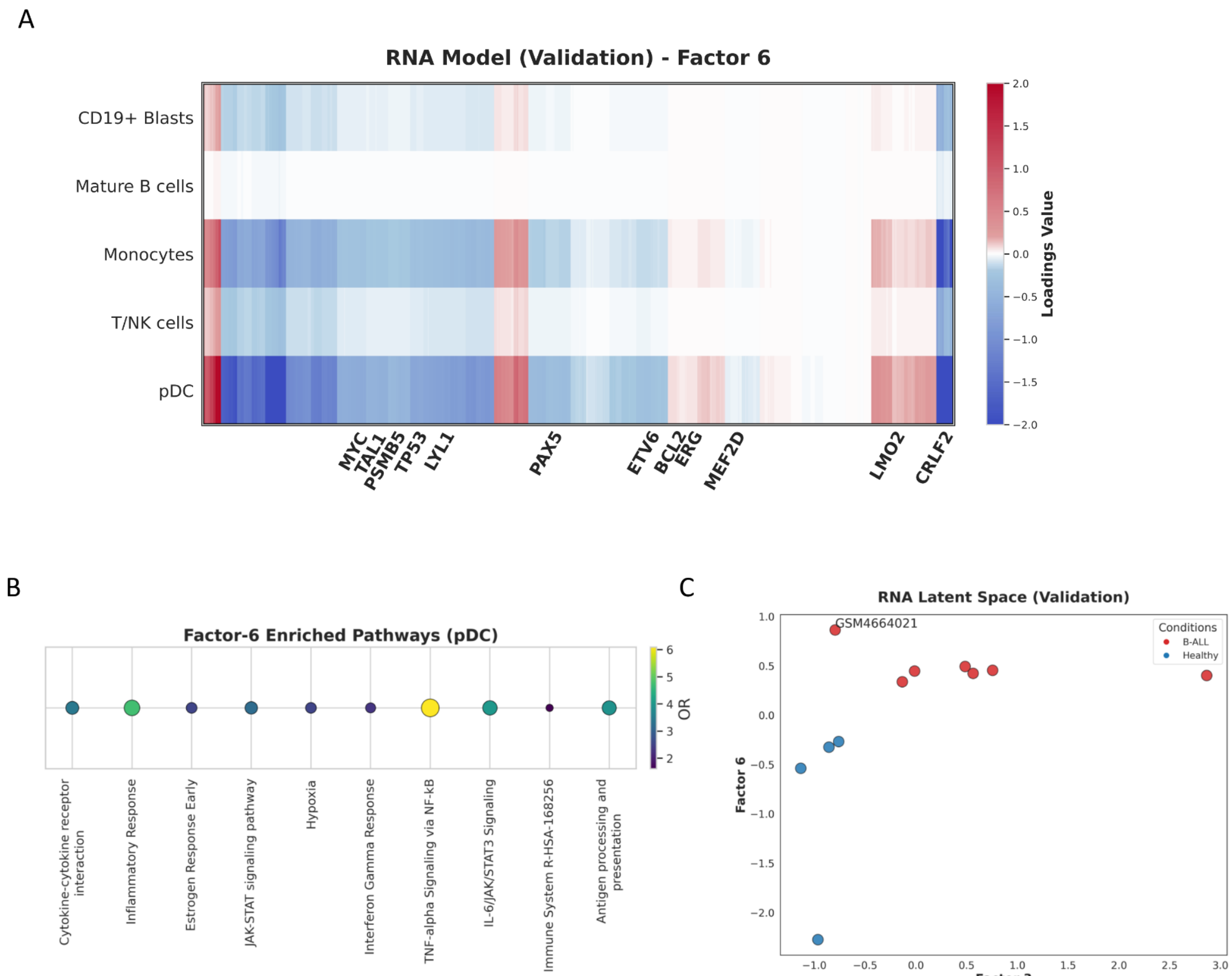

Fig. 9: Validation of the pDC factor by fitting MANTRA the RNA-seq tensor derived from an independent cohort of pediatric B-ALL patients . A) MANTRA again identified a latent factor primarily driven by pDCs. B) ORA of gene loadings again revealed over-representation of pathways related to immune activity,consistent with the findings from the discovery cohort. C) A scatter plot shows good separation between healthy donors and B-ALL patients. The patient with the lowest immune activity (highest score of Factor 6) is highlighted.

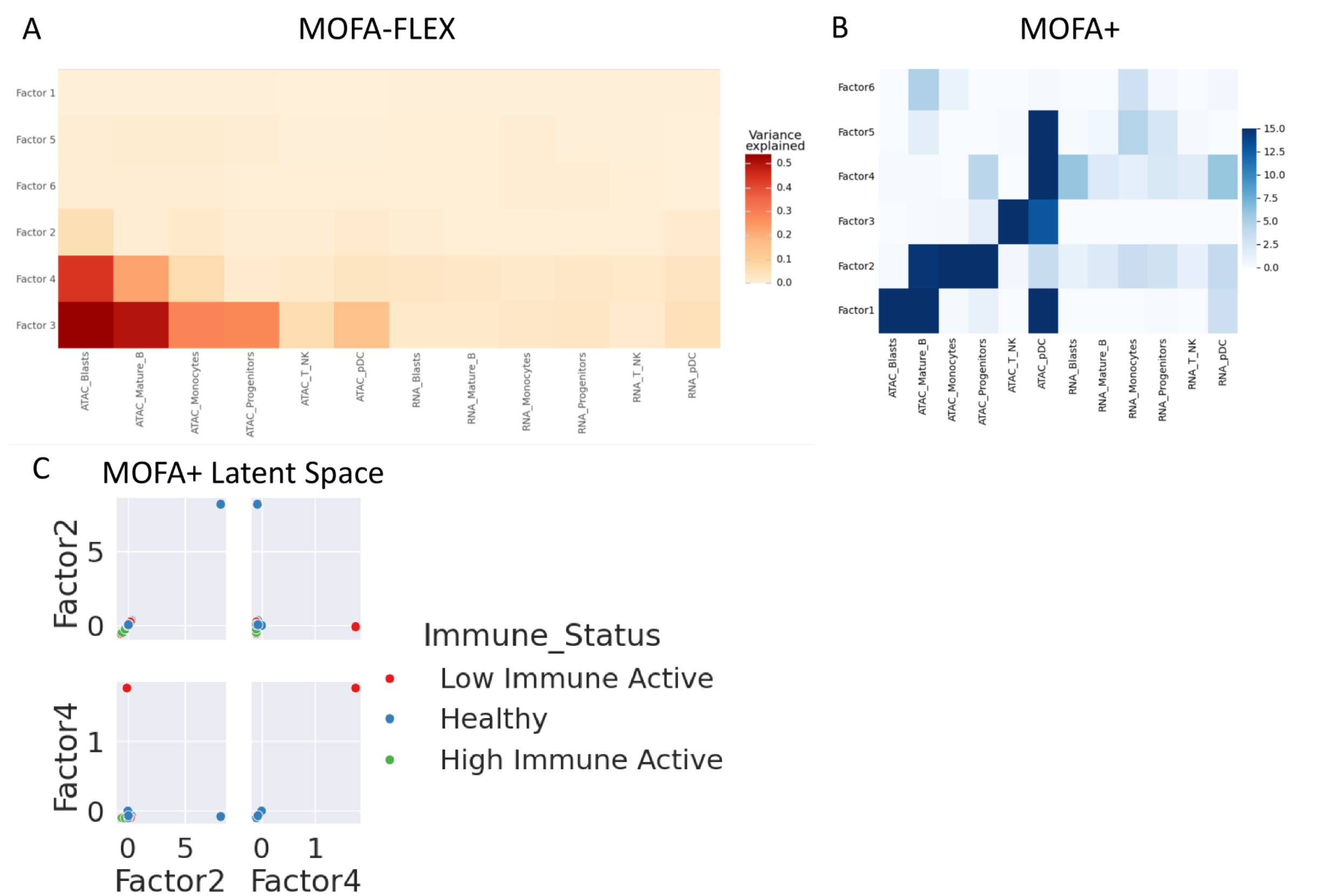

Supplementary Figure S9: MOFA+ and MOFA-FLEX trained on the Multiome data modelling tensor slices as views. None of the factors identified by MOFA-FLEX explains variance in the pDC-RNA view (A). The two factors identified by MOFA+ which do explain some variance in the pDC-RNA view (B) do not capture the immune high/low phenotype revealed by MANTRA (C).

Response to Reviewer #2

Comment: Overall, the ms lacks a more rigorous benchmarking and crucial details. While efforts have been made to perform comparison for the synthetic data with PARAFAC, the details of the data and potential dependence on the data size were not addressed. Similarly, the analysis of the 2 biomedical applications remains on a rather superficial level both on the benchmarking as well as for the biomedical interpretation. For benchmarking, the authors should not only consider MOFA but also more recent developments such totalVI, MOFA+, scITD, MONFIT) and also use additional measures like ARI, silhouette score, or explained variance. Furthermore, a benchmarking should also include dependency on MUSIC model parameter choices, structured sparsity and linearity which should be also addressed in a corresponding limitation analysis.
Response:
On synthetic data: We have now substantially extended our synthetic benchmark to better characterize the behaviour of MANTRA across multiple relevant data characteristics and modelling choices. In particular, we now systematically vary (i) the number of latent factors (default:20), (ii) number of samples (default:20), (iii) sparsity in the data (default:0.1), (iv) signal-to-noise ratio (default:10 dB), (v) n_features (default:5000) and (vii) random noise (default = 1). Sparsity was determined by the following observations in real-world RNA tensor sparsity (%12 in the ALL data) and ATAC tensor sparsity (32% in the ALL data). Additionally we have run an additional experiment to mimic real-world applications where it is unclear what the true rank of the data is. In this experiment, we assess how robust MANTRA and Parafac are against mis-specified ranks. To disentangle the effect of these individual factors, we chose a default (given in parenthesis) and varied one factor at a time. This revealed that MANTRA has comparable performance to Parafac in the high SNR/high sample ratio and a substantially improved reconstruction error in the noisy data regime (low SNR,low sample_size, high std and high sparsity), mimicking real-world datasets with moderate-to-low SNR and moderate-to-high SNR (Fig-2). In addition, due to its sparsity priors it is substantially more robust against rank mis-specification than Parafac as the structured sparsity prior encourages the model to switch off un-needed factors (i.e. drive the factor scores to 0).
We have now added a detailed description of this benchmark to the Methods and Results section.

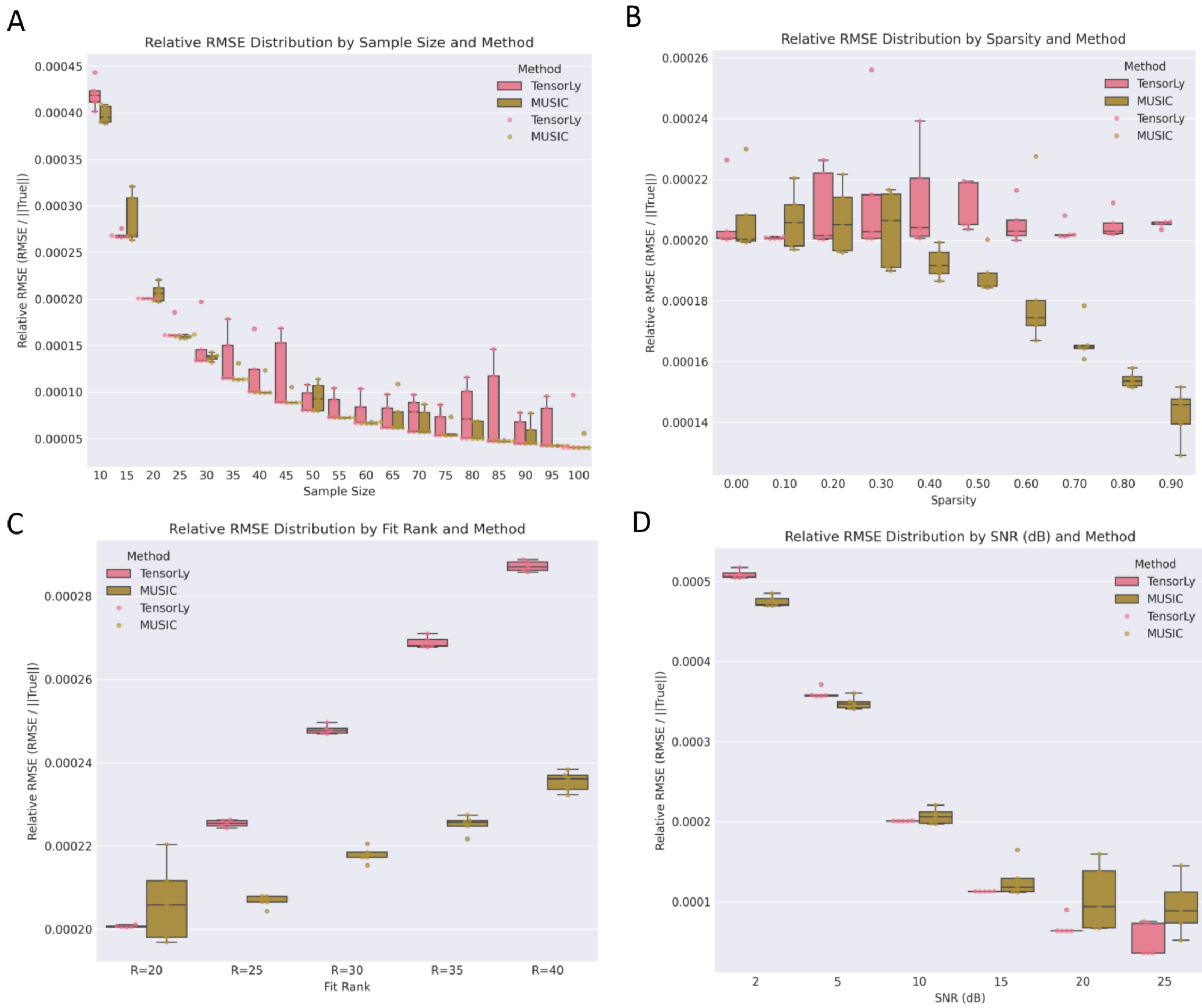

Fig. 2: Relative RMSE across typical data characteristics and modelling choices for Tensorly's Parafac method and MANTRA. Both methods are benchmarked for varying sample size (A), sparsity levels (B), rank (C) and signal-to-noise-ratio (C). RMSE was computed relative to the norm of the simulated (true) tensor to aid interpretability.

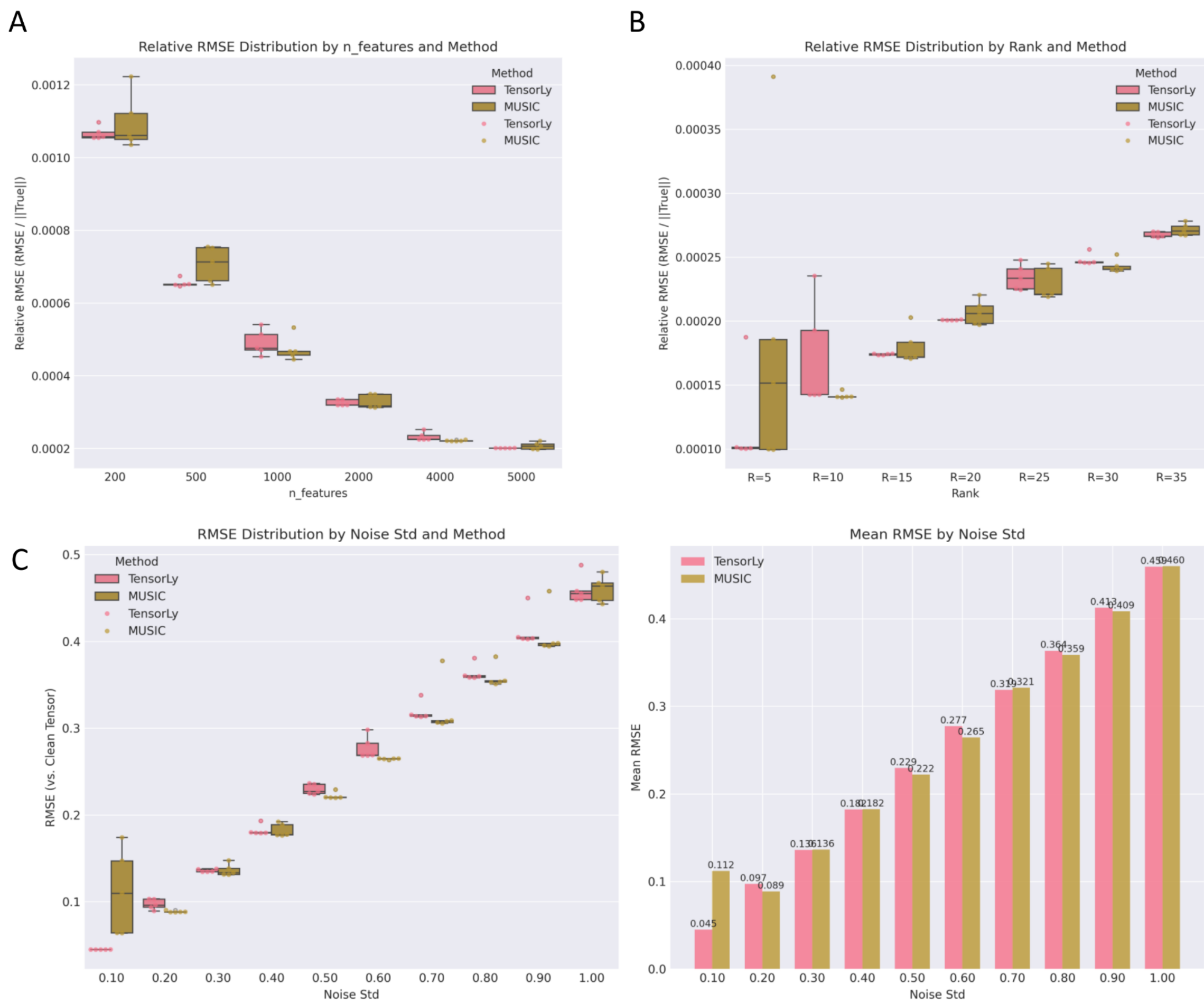

Supplementary Figure S1: Relative RMSE for varying numbers of features(A) and rank for simulated data (B) and for noise Std(C).

On biomedical data: In addition, we have now also included more recent methods for benchmarking the biomedical data. We had used the MOFA+ implementation already in the initial submission, and have added MOFA-FLEX as the most recent release in the family of group-factor-analysis based models. In addition we have added a comparison to scITD for B-ALL patient data (Fig-6). To facilitate this direct comparison, we also ran MANTRA on the RNA-seq B-all patient data alone. We could not benchmark against scITD for the cytokine data as scITD explicitly expects scRNA-seq data of donors as input and has tailored the entire computation pipeline to this type of data. For similar reasons, we could not benchmark against totalVI and MONFIT as the first is a specialised tool for paired transcriptome and protein measurements such as CITE-seq data in single cells and the latter designed to model longitudinal data.

For the cytokine data, we have now included a quantitative comparison to MOFA+ and MOFA-FELX using ARI and NMI on the learnt latent space (Fig.4). This confirmed that MANTRA was

able to learn better representations (in terms of clustering performance) compared to multi-view integration methods.

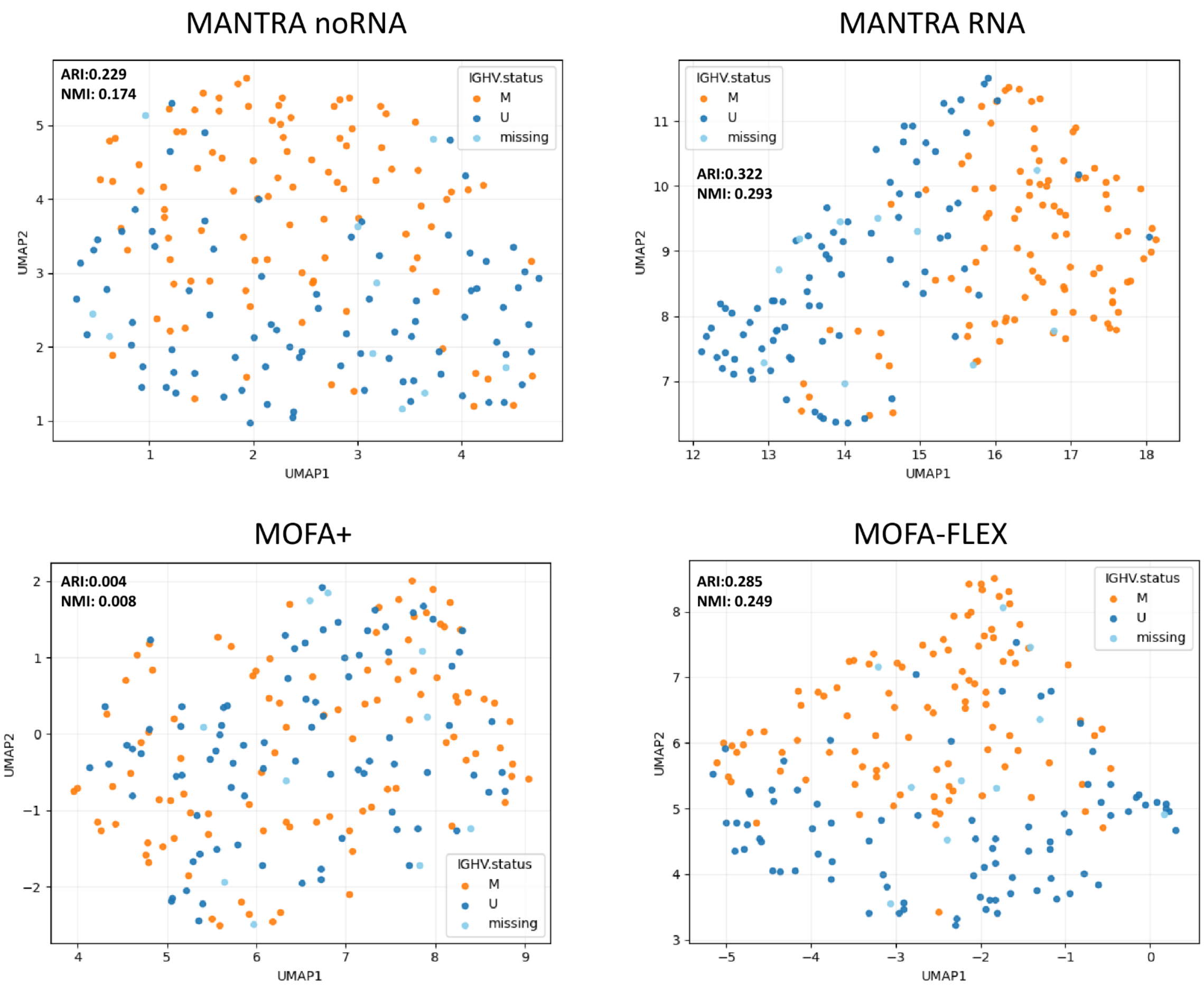

Fig. 4: UMAP of patient embeddings learned by MANTRA (single-view, top left), MANTRA (multiview, top right), MOFA+ (bottom left) and MOFA-FLEX (bottom right). Both MOFA+ and MOFA-FLEX were trained based on all slices of the drug viability tensor as well as the RNA-seq view. Adjusted Rand Index (ARI) and Normalized Mutual Information (NMI) with respect to Leiden clusters were computed to quantify how well individual methods could separate patients grouped by IGHV status.

For the patient B-ALL data, all methods could separate healthy donors from cancer patients, but only MANTRA was able to stratify patients into two subpopulations, driven by a pDC factor. Notably, scITD identified only the major source of variation between patients and healthy donors driven by blasts and and did not identify the pDC factor stratifying patients that MANTRA had revealed, with all scITD factors primarily being active in blasts.

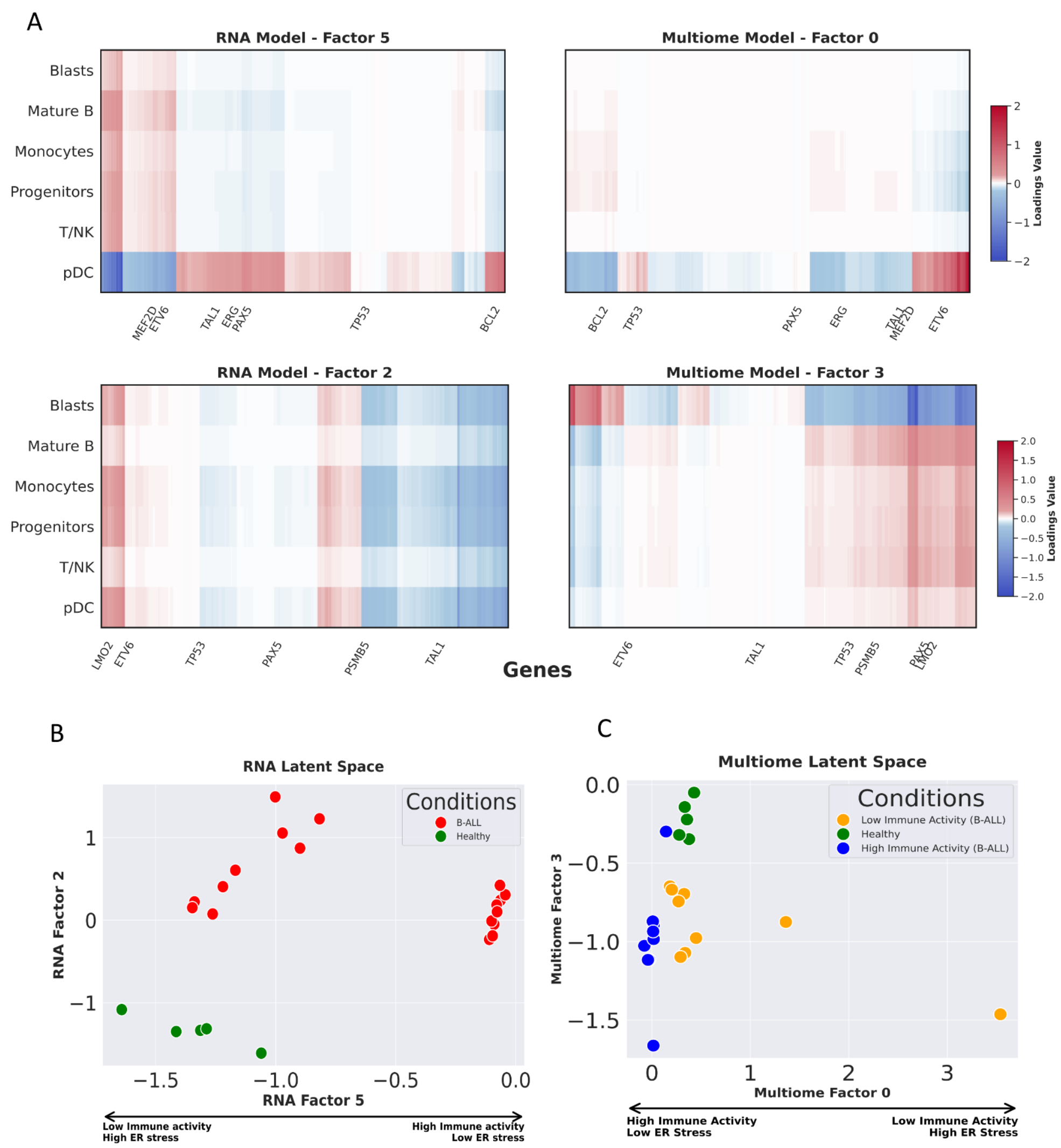

Fig. 7: Characterization of the MANTRA factors explaining variation between healthy donors and patients (Factor 2/RNA and Factor 3/Multiome) as well as factors delineating two B-ALL subpopulations (Factor 5/RNA and Factor 0/Multiome). A) Cell-type loading plots for the RNA model (left) show that Factor 5 is primarily active in pDCs, whereas Factor 2 explains variation in most cell types. Cell-type loading plots for the Multiome model (right) show patterns that are consistent with the RNA model, with cell-type attributions more focussed on individual cell-types than for the RNA model. B) Scatter plots separate healthy donors from B-ALL patients and reveal a B-ALL sub-population characterised by high immune activity/low ER stress. This subpopulation was consistently found for MANTRA models trained on RNA (left) and Multiome (right). Patients from the high immune activity/low ER stress cluster identified in the RNA latent space (right cluster) are shown in blue in the Multiome latent space to illustrate the consistency

between the embeddings. Similarly, patients from the low immune activity/high ER stress cluster identified in the RNA latent space (left cluster) are shown in yellow in the Multiome latent space.

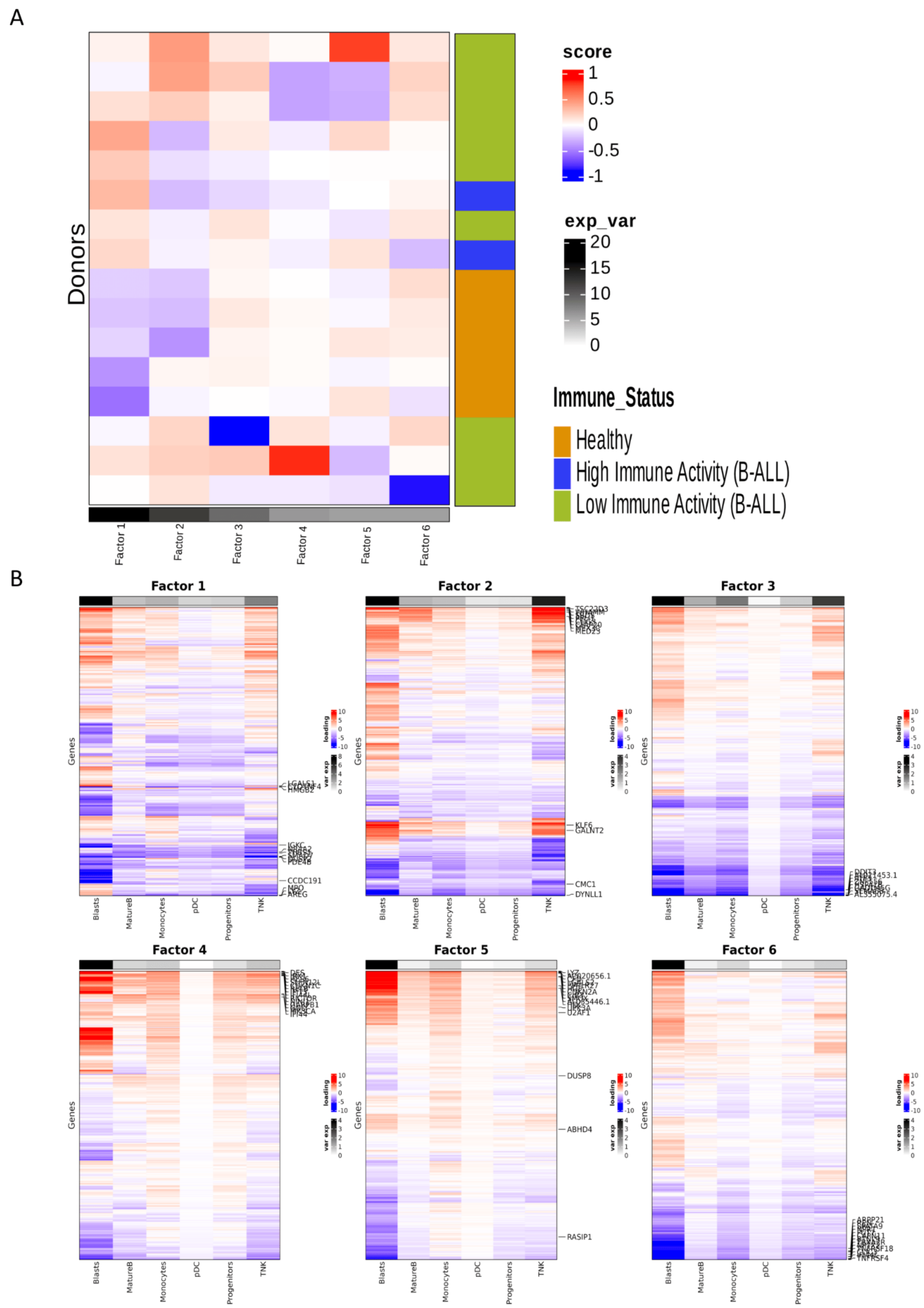

Fig. 6: scITD results on RNA-seq data. A) Donor factor scores. scITD was able to separate healthy from ALL patients (via Factor 1), but could not recover the additional structured variation between patients revealed by MANTRA . B) Cell-type-Gene factor loadings learnt by scITD mainly captured variation driven by blasts and did not identify the pDC-driven factor found by MANTRA.

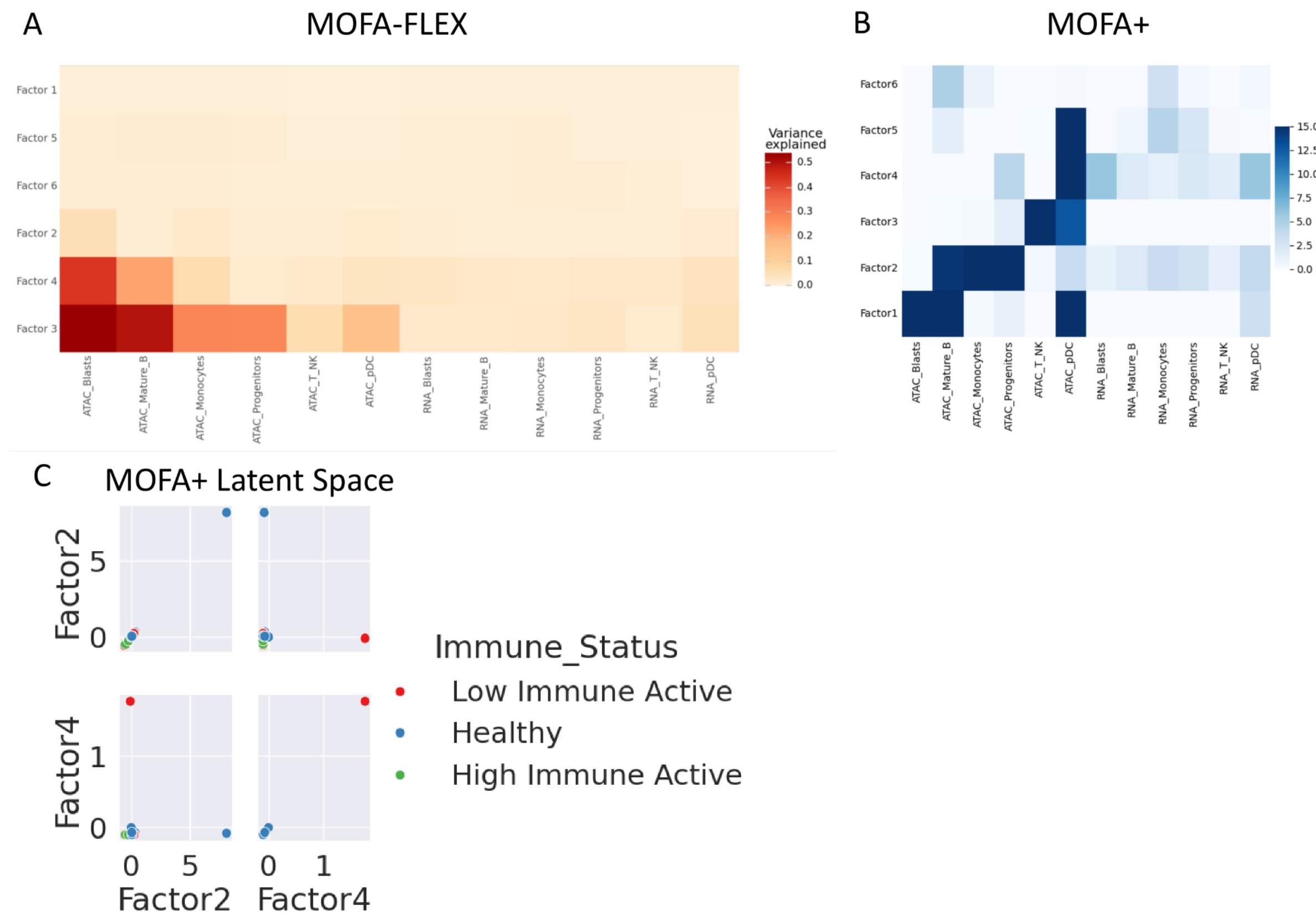

Supplementary Figure S9: MOFA+ and MOFA-FLEX trained on the Multiome data modelling tensor slices as views. None of the factors identified by MOFA-FLEX explains variance in the pDC-RNA view (A). The two factors identified by MOFA+ which do explain some variance in the pDC-RNA view (B) do not capture the immune high/low phenotype revealed by MANTRA (C).

Sensitivity analysis: Finally, we have also performed a sensitivity analysis of MANTRA's hyperparameters, namely the noise priors α and β. This revealed that while varying α from 1.0 to 5.0 and correspondingly adjusting β from 0.5 to 2.5 resulted in remarkably consistent performance across sparsity levels (with RMSE differences ≤ 0.008), the specific combination of $\alpha = 1.0$, $\beta = 0.5$ demonstrated the best overall performance, achieving the lowest average RMSE at the highest sparsity level (0.3) with 0.4554 ± 0.0248. The stability of results across this parameter range suggests MANTRA is robust to moderate variations in noise prior specification, though optimal performance favors the more conservative priors $\alpha = 1.0$, $\beta = 0.5$.

Average MANTRA RMSE Across Sparsity Levels (Mean ± SD)

Hyperparameters (a,b)	Sparsity 0.0	Sparsity 0.1	Sparsity 0.2	Sparsity 0.3
(1.0, 0.5)	0.4639 ± 0.0287	0.4633 ± 0.0236	0.4702 ± 0.0209	0.4554 ± 0.0248
(2.0, 1.0)	0.4640 ± 0.0287	0.4633 ± 0.0236	0.4699 ± 0.0209	0.4555 ± 0.0248
(3.0, 1.5)	0.4640 ± 0.0288	0.4633 ± 0.0236	0.4700 ± 0.0210	0.4555 ± 0.0248
(5.0, 2.5)	0.4639 ± 0.0287	0.4634 ± 0.0237	0.4698 ± 0.0209	0.4556 ± 0.0248

Comment: The overserved improved classification of patients looks indeed promising but so far it is more an interesting observation and would require some validation either by targeted experiments or independent data. In this respect, a more detailed biomedical description as well as discussion is lacking like for the IGHV and Trisomy 12 status in the disease context.
Response: We agree that external validation of our findings would substantially strengthen the manuscript. Following your suggestion, we have identified an independent cohort of pediatric B-ALL patients, profiled with single cell RNA-seq [2]. Since no multiome data was available for this validation cohort, we first conducted some additional experiments on the discovery cohort. Specifically, we ran MANTRA on the scRNA-seq data to assess whether this data-view was sufficient to identify the sub-population identified based on the multiome. Indeed, MANTRA found a pDC factor that clearly separated 2 B-ALL sub-populations. We then performed a more in-depth biological characterization of the gene loadings of the pDC factor via gene-set analysis, revealing immune-active pathways such as Interferon Alpha Response and TNF-alpha signaling via NF-kB that were active in this factor. This was consistent with our findings from the multiome data; we also confirmed that the identified sub-populations indeed matched. Next, we set out to assess whether MANTRA could also identify a pDC factor characterized by immune-active pathways in the validation data. Indeed, we found that one of the factors learnt by MANTRA was primarily active in pDCs. We next investigated whether this factor was again driven by pathways related to immune activation. Supporting our hypothesis derived from the discovery cohort, pathway analysis of the loadings again identified immune-active pathways.

To further assess the consistency between the MANTRA models learnt on the discovery and validation cohorts respectively, we projected the donors from the validation cohort into the MANTRA space learnt on the discovery cohort (Fig. S8). To this end, we froze both cell type loadings and gene loadings, learning only patient-level scores by optimizing the ELBO. Projected donor scores matched those immune activity levels learnt directly on the validation cohort. That is, validation patients for which MANTRA inferred high pDC immune activity when trained directly on the validation cohort, clustered with discovery patients with high pDC immune activity when projected onto the MANTRA space learnt from the discovery cohort. Similarly, healthy donors from the validation cohort clustered with healthy donors from the discovery

cohort. Taken together this confirmed that pDCs are a driver of inter-patient heterogeneity in pediatric B-ALL patients, separating patients with pDCs in a high immune active and low immune active state.

Importantly, when applying MANTRA to a second validation cohort of pediatric T-ALL patients as negative control, we did not find such pDC factor driving inter-patient variability, suggesting that our finding is specific to B-ALL (Fig. S9).
We have also added a more detailed biomedical description on the interpretation of the blast factor.

[2] Bailur, Jithendra Kini, et al. "Risk-associated alterations in marrow T cells in pediatric leukemia." *JCI insight* 5.16 (2020): e140179.

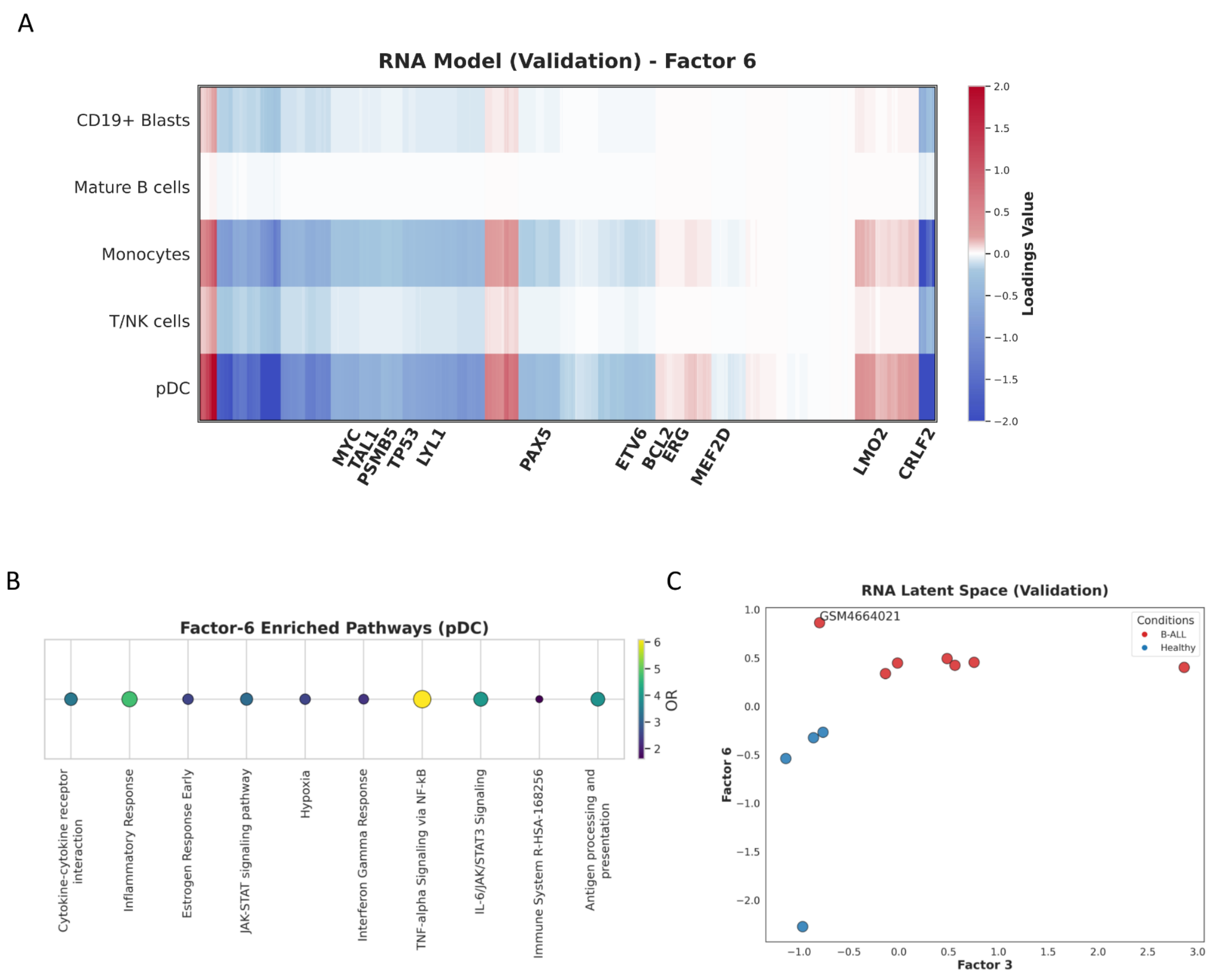

Fig. 9: Validation of the pDC factor by fitting MANTRA the RNA-seq tensor derived from an independent cohort of pediatric B-ALL patients . A) MANTRA again identified a latent factor primarily driven by pDCs. B) ORA of gene loadings again revealed over-representation of pathways related to immune activity, consistent with the findings from the discovery cohort. C) A

scatter plot shows good separation between healthy donors and B-ALL patients. The patient with the lowest immune activity (highest score of Factor 6) is highlighted

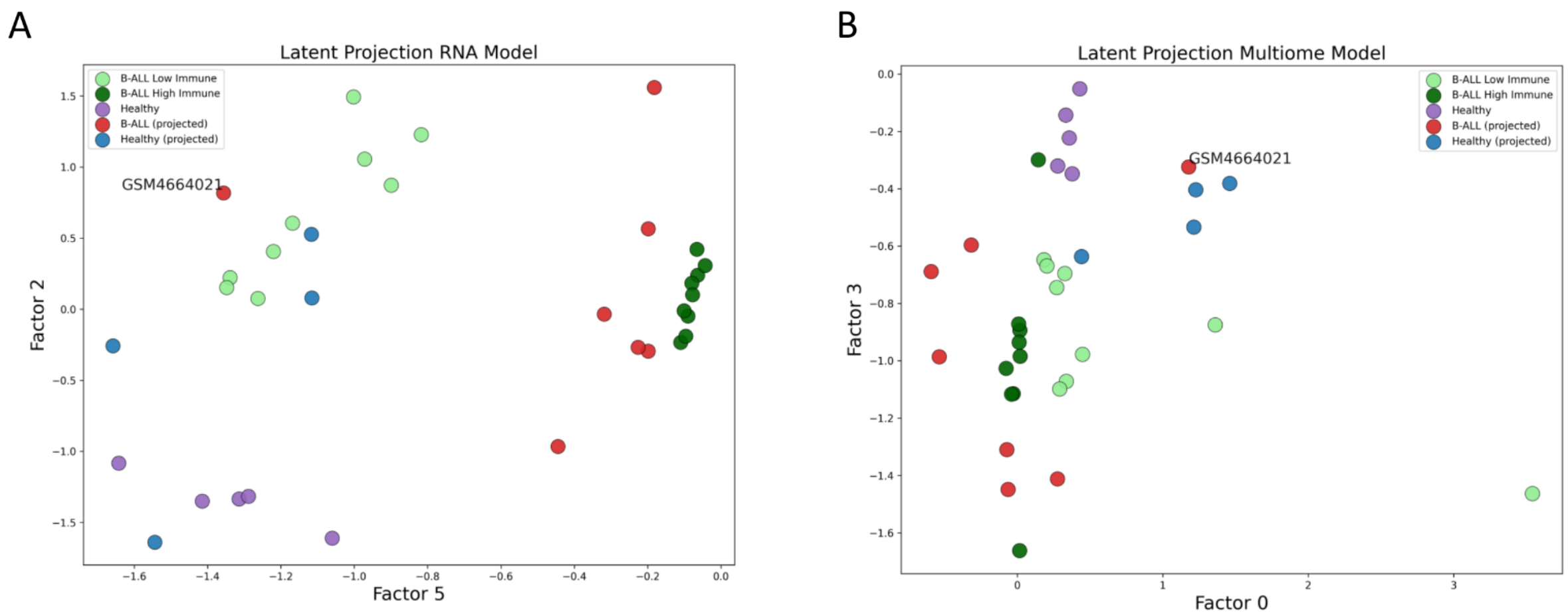

Supplementary Figure S8: Embeddings of MANTRA models trained on the RNA tensor of the discovery cohort only (A) and trained on the Multiome (B). Samples from the validation cohort were projected into this space by freezing the feature embeddings and cell-type embeddings. Healthy donors from the validation cohort cluster with healthy donors from the discovery cohort; patients form the validation cohort cluster largely with the high immune subtype, but notably the sample with the lowest immune activity in the MANTRA model trained directly on the validation cohort (GSM4664021) clusters with the low immune patients of the discovery cohort.

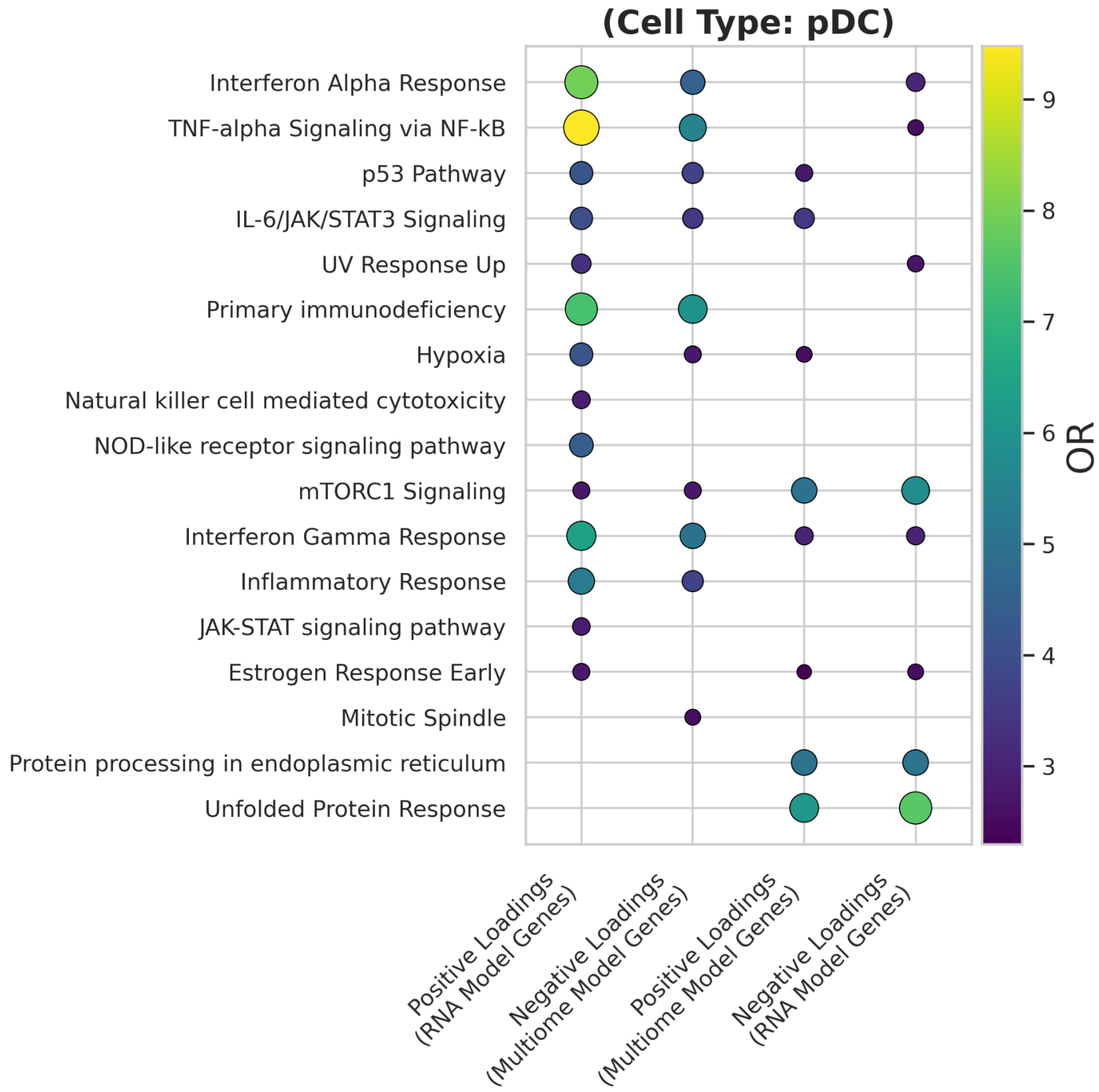

Fig. 8: Pathway enrichment analysis of the loadings of the pDC factor via over-representation analysis (ORA). We performed separate ORA enrichments for positive and negative loadings, for both the RNA-based MANTRA model as well as the multi-view MANTRA model.

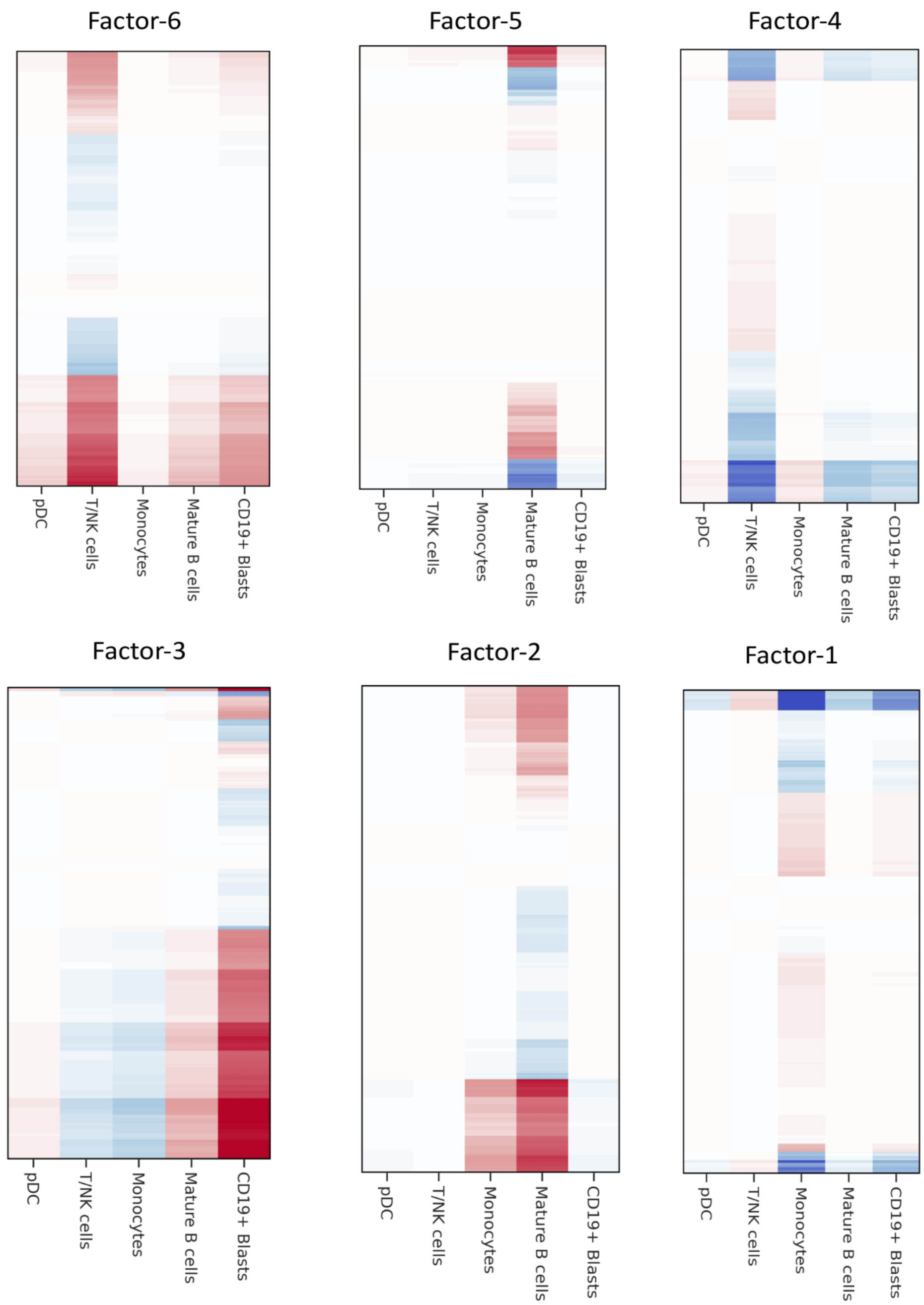

Supplementary Figure S9: Loadings of a MANTRA model trained on a cohort of T-ALL patients. None of the factors was active in pDCs, suggesting that the pDC axis of variation identified by MANTRA in B-ALL is specific to B-ALL.

Comment: The manuscript could also be better organised. For instance, the Methods section seems to be misplaced, and some figures are not referred to or are not included in the general story (see also diverse comments on minor points below). Some figures require also more explanations and should be discussed in more detail.
Response: We have now substantially revised the manuscript, fixed the inconsistencies and typos from the Minor Comments and made sure all figures are referenced and the overall story is told in a more coherent manner with more in-depth biomedical discussion.

Minor Comments:

• Typo in introduction: "unsupervised modelling approaches are can reveal..." → remove "are".

We have fixed this.

• Typo in first sentence of results and first sentence in 2.1: space between "MUSIC" and the "," should be removed. This also holds for "MUSIC 's" ... so probably the latex shortcut must be modified.

Fixed.

• The synthetic data used for performance test should be better detailed (and also mentioned in the Results text and not only in the Methods? What size were the tensors? How does the size affect the comparison?

Fixed, see also the response above.

• Fig. 3 seems to be referred as Fig. 4 in the text? Colour schemes for the column and raw colours are not defined. Maybe worth to put a label to the colour bar? The claim of the improved patient embedding should be better quantified by at least giving the cluster composition. The meaning for Fig. 4 remains unclear?

We have extended this section and revised it substantially.

• Missing or broken figure ref in "In this multi- view setting, MUSIC uncovered a clear separation of patients based on their IGHV mutation status and Trisomy 12 status, two well-characterized prognostic markers in CLL (Fig. ??)." on page 6.

Fixed and revised.

• Typo in caption of Fig. 5 "We can see than MOFA is not able to disentangle ... " - change "than" into "that".

Fixed.

• Supplementary Figures should be referred to as Supplementary figures and not consecutive figs.

Fixed.

• For me the separation between Healthy and ALL patients in Fig. 6 (a) seems to be driven by Factor 3 and not Factor 0? More explanation needed here as well as for the patterns in Fig. 6b.

Fixed and revised with substantially more details, new analyses and external validation.

• Caption of Fig. 7 seems to be incomplete.

Fixed.

• "What do the authors mean with "Special cases of MUSIC are group factor analysis as implemented in MOFA ..."?

If MANTRA is being run on a collection of rank-2 tensors only, it becomes a standard group factor analysis as implemented in MOFA.

• "Space" issue at the end of page 6 after "(Fig. 6)" and "."

Fixed.

• Use "\times" instead of "x".

Fixed.

• Methods seems to be a bit deplaced ... should be wither before results or after Discussion.

Thank you. We now follow the guidelines of MSB, which places Methods after Discussion.

• Choices for alpha and betha for the inverse Gamma distribution should be commented.

We used standard weakly informative choices for alpha and beta.

3rd Mar 2026

Manuscript Number: MSB-2025-13234R
Title: Interpretable multi-omics integration across mixed-order tensors with MANTRA
Author: Kevin de Azevedo
Yusuf Oruc
Florian Buettner

Dear Florian,

Thank you for submitting your revised manuscript. We have now received feedback from the original Reviewer #2, who agreed to evaluate your revised study. Since Reviewer #1 was unavailable for this round, we also asked Reviewer #2 to assess your responses to the points raised by Reviewer #1. Overall, Reviewer #2 is supportive and believes that the revision has addressed most of the concerns raised by both reviewers.

Before we can formally accept your manuscript, we ask that you address the following:

1. The remaining, relatively minor issues identified by Reviewer #2.

2. Although not explicitly requested in the previous review, we encourage you to consider strengthening the "computational benchmarking" suggested by Reviewer #2. This, however, is not mandatory for acceptance.

On a more editorial level:

1. If possible, kindly provide the manuscript in DOCX format instead of LaTeX.

2. Remove the "Authors' contribution" section from the manuscript file.

3. Please remove all figures from the manuscript file and retain the main figure legends at the end of the manuscript.

4. For main figures, please provide individual production-quality figure files in .eps, .tif, or .jpg format (one file per figure).

5. Figure 7: Label C is missing from the figure caption; please provide it.

6. Add missing callouts for Figure 2A-D, Figure 6A-B, Figure 7C, Figure 9A-C, and Appendix Figures S3 and S6.

7. Please address the following issues in the figure legends:
- Figure titles for Figures 1-9 are not provided; kindly add them.
- For Figures 2A-D, please define the box plots (minima, maxima, centre, box bounds, whiskers, percentiles) in the legends.

8. All supplementary materials and figures should be compiled into a single separate "Appendix" file. Please adjust the nomenclature and in-text callouts accordingly (e.g., Appendix Figure Sx). Appendix figure legends should be placed below the corresponding figures in the Appendix. The Appendix should be titled "Appendix for [manuscript title]" and begin with a table of contents listing all included items along with their corresponding page numbers.

9. The references must be formatted according to the Molecular Systems Biology style. Please list up to 10 co-authors before adding "et al." Citations should be listed in alphabetical order.

10. Please provide:
-A standfirst text summarizing the study in one or two sentences (approximately 250 characters including spaces), three to four bullet points highlighting the main findings, and
- A synopsis image (550 px width × 400-600 px height, PNG format) for promotion on our homepage. You may refer to published papers for examples.

Please provide a revised manuscript with marked changes, along with a point-by-point response to the reviewer' comments.

Use the following link to submit your revised paper:

https://msb.msubmit.net/cgi-bin/main.plex

As a matter of course, please make sure that you have correctly followed the instructions for authors as given on the submission

website.

Thank you for submitting this paper to Molecular Systems Biology.

Kind regards,
Jingyi

Jingyi Hou, PhD
Senior Editor
Molecular Systems Biology

If you do choose to resubmit, please click on the link below to submit the revision online before 2nd Apr 2026.

https://msb.msubmit.net/cgi-bin/main.plex

*** PLEASE NOTE *** As part of the EMBO Press transparent editorial process initiative (see our Editorial at https://dx.doi.org/10.1038/msb.2010.72 , Molecular Systems Biology will publish online a Review Process File to accompany accepted manuscripts. When preparing your letter of response, please be aware that in the event of acceptance, your cover letter/point-by-point document will be included as part of this File, which will be available to the scientific community. More information about this initiative is available in our Instructions to Authors. If you have any questions about this initiative, please contact the editorial office (msb@embo.org).

Reviewer #2:

First, I'd like to thank the authors for their substantial revisions that address most of my concerns. In particular, the benchmarking and the inclusion of the validation cohort data makes the ms significantly more substantial.

For the benchmarking, the authors may still consider:

- a synthetic mixed-order multi-view benchmark that would further reinforce the core conceptual contribution (would be nice but not urgent),

- a computational runtime or computational scalability comparisons that would allow the readers to get an idea about computational demands and safe them to perform this analysis by themselves (would improve the benchmarking significantly).

The discussion briefly addresses linearity and interpretability trade-offs. However, a more explicit limitation paragraph could further strengthen the manuscript, particularly regarding, identifiability and rotational invariance; situations where mixed-order modeling may not outperform matrix approaches; and computational scalability.

Minor points:

- The white arrows in the black box of Fig. 1 are not self-explanatory and not clear. Are they needed?
- Fig. 6: In (A) the font size is too small and needs adaptation. In (B) the "Immune_Status" legend looks stretched and should be corrected.

Thus, the authors have addressed my major concerns, and the revised manuscript is substantially improved in methodological rigor, benchmarking breadth, and biological validation.

While minor enhancements (e.g., scalability discussion, explicit limitations) could further strengthen the manuscript, the core scientific and technical claims are now convincing.

****

Reviewer #2's evaluation of your response to Reviewer #1's comments:

"Please find below my remaining comments regarding the point-by-point responses to Reviewer 1:

The comment on benchmarking is addressed - where the comments of reviewer 2 wrt to computational benchmarking remain.
Furthermore, in Fig. 2 there is still "MUSIC" instead of new name "MANTRA" mentioned. In the caption there is a typo "aer" -> "are" (which is not on the rebuttle letter but in the ms).
The concerns about Fig. 3 are solved.
The supplementary Fig. 2 in the rebuttal letter is the actual Supplementary Fig. S3 in the ms. This figure is not referred in the text and not described which makes the necessary comment invisible (same holds true for S6!).
Concerns about Fig. 4 are resolved.
For me also the concerns on model parameters are addressed.
The response wrt to Eq. 1 is rather minimalistic and should be clarified (maybe also in the text)
Argument for the lacking MAFISTO comparison is valid.

Thus, the bottom line is that the main points have been addressed but that some final edits are needed."

Point-by-point response

We thank the reviewer #2 for the constructive feedback.
We now have revised the paper and added a multi-view benchmark and scalability analysis.

We respond to all comments below in a point-by-point manner, with our response highlighted in blue, and have revised the manuscript accordingly (changes in the manuscript are highlighted in red).

Response to Reviewer #2:

Comment: For the benchmarking, the authors may still consider:
- a synthetic mixed-order multi-view benchmark that would further reinforce the core conceptual contribution (would be nice but not urgent),

Response: We thank the reviewer for this helpful suggestion. To further evaluate MANTRA's ability to recover view-specific latent structure, we added an additional multiview simulation benchmark in which the underlying ranks differed between views. This setting is particularly relevant for multi-omics data, where individual modalities may exhibit different latent complexities and where some factors may only be relevant to specific views.

To this end, we generated two synthetic tensors sharing the same sample and condition modes but with different simulated ranks. Each dataset contained 20 samples, 5 conditions, and 3,000 or 5,000 features in the two views. Sparse Gaussian factors were generated with sparsity 0.3 using rank pairs R5+R10, R5+R20, R5+R30, R10+R20, and R10+R30, where, for example, R5+R10 denotes rank 5 in the first view and rank 10 in the second view. For each setting, we ran five random seeds and fitted the models at the larger of the two simulated ranks.

We compared native multi-view MANTRA with TensorLy PARAFAC applied to a concatenated tensor, since PARAFAC does not directly support multiple views. As shown in the newly added Supplementary Figure S2, MANTRA more effectively removed unnecessary factors when rank differences between views were large and consistently achieved lower per-view RMSE than PARAFAC. These results further support that MANTRA can suppress view-irrelevant factors while maintaining accurate view-specific reconstruction, highlighting its suitability for sparse and interpretable multi-view integration.

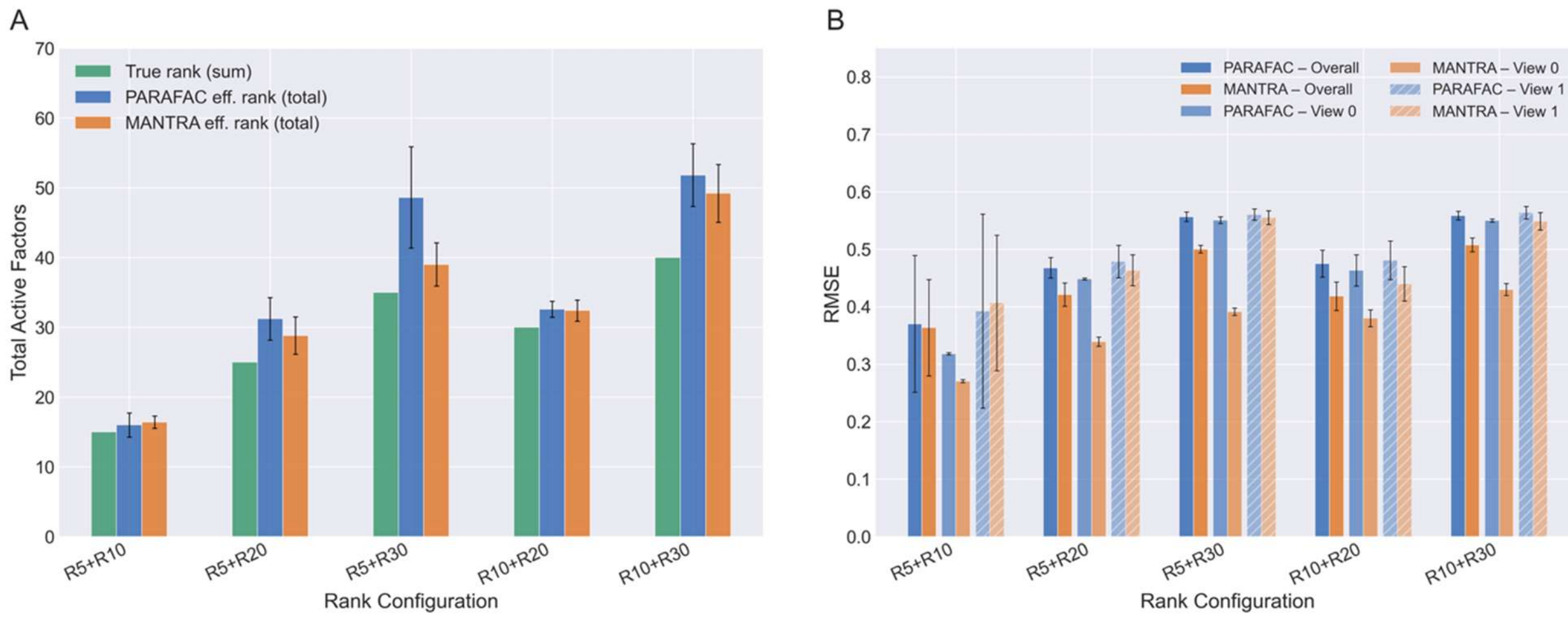

Supplementary Figure S2: Multi-view simulation experiment evaluating whether MANTRA can recover view-specific latent structure while shrinking factors that are not relevant for a given view. Rank configurations indicate the simulated rank of each view; for example, R5 + R10 denotes a simulation with rank 5 in View 0 and rank 10 in View 1. (A) Comparison of the summed simulated rank with the total effective rank inferred by PARAFAC and MANTRA. (B) Overall and per-view RMSE comparison across rank configurations. MANTRA estimates effective ranks closer to the simulated rank configurations and generally achieves lower RMSE than PARAFAC, particularly for individual views. These results demonstrate that MANTRA can learn sparse and interpretable view-specific representations while maintaining accurate reconstruction performance.

Comment: - a computational runtime or computational scalability comparisons that would allow the readers to get an idea about computational demands and safe them to perform this analysis by themselves (would improve the benchmarking significantly).

Response: We thank the reviewer for this comment. To evaluate the computational scalability of MANTRA, we measured both per-iteration runtime and total wall-clock time while systematically varying the number of features, samples, and latent rank. Synthetic datasets were generated with 20 samples, 5 conditions, 5,000 features, rank 20, sparsity levels of 0.1 and 0.3, and a target SNR of 10 dB. We varied feature size (1,000–10,000), sample number (10–200), and rank (10–50), performing three independent trials per setting using different random seeds.

MANTRA was trained for 5,000 epochs with early stopping disabled to ensure consistency across runs. That choice was motivated by the fact that the early stopping parameters would introduce another layer of complexity to the scalability analysis. Therefore; no early stopping was used in this benchmark. We compared MANTRA against TensorLy PARAFAC and observed that MANTRA maintained relatively stable per-epoch and wall-clock runtimes across the tested settings, whereas PARAFAC runtime increased more strongly with increasing sample size and rank. These results indicate that MANTRA remains computationally scalable across a broad range of data dimensions and latent complexities.

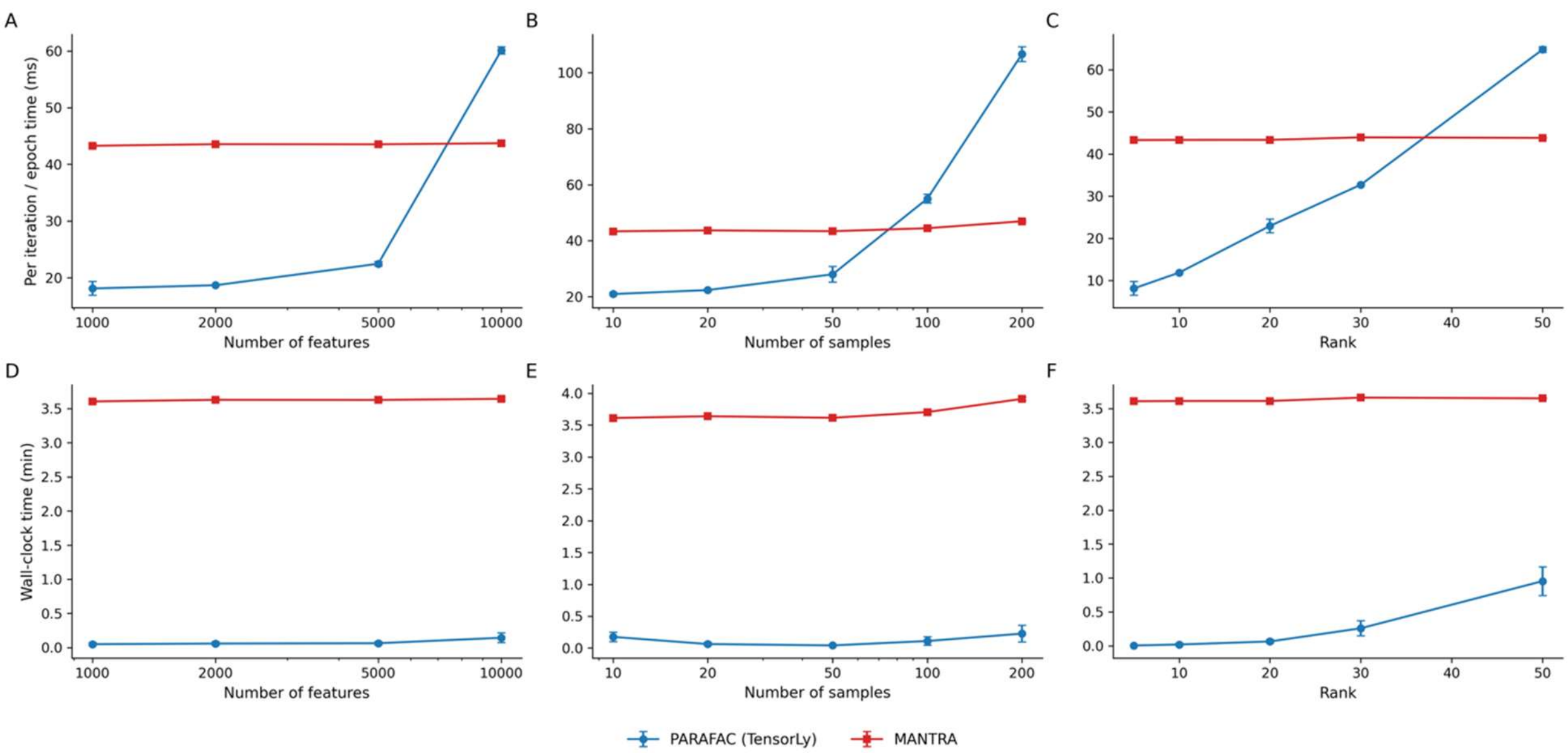

Supplementary Figure S3: Computational scalability of MANTRA. Per-iteration or per-epoch runtime is shown in milliseconds (ms) for increasing numbers of features **(A)**, samples **(B)**, and latent rank **(C)**. Total wall-clock runtime is shown in minutes (min) for the corresponding experiments with varying numbers of features **(D)**, samples **(E)**, and rank **(F)**. MANTRA showed relatively stable per-epoch runtime across increasing feature numbers, sample sizes, and ranks, whereas PARAFAC runtime increased more strongly, particularly for larger sample sizes and higher ranks. Similarly, MANTRA maintained a nearly constant total wall-clock time across the tested settings. These results indicate that MANTRA remains computationally scalable across the simulated ranges of features, samples, and latent ranks considered here.

Comment: The discussion briefly addresses linearity and interpretability trade-offs. However, a more explicit limitation paragraph could further strengthen the manuscript, particularly regarding, identifiability and rotational invariance; situations where mixed-order modeling may not outperform matrix approaches; and computational scalability.

Response:
Now we have added the discussion for the trade-offs:

Despite its conceptual and practical advantages, MANTRA has certain limitations that warrant consideration. First, similar to other latent variable and factorization frameworks, the model faces inherent challenges regarding identifiability and rotational invariance. Second, there are specific settings where mixed-order modeling may not substantially outperform standard matrix-based approaches. In datasets where structural dependencies are negligible, or where extreme sparsity and high noise levels impede the robust estimation of multi-dimensional components, simpler matrix factorizations may prove equally effective or more stable. Finally, while the fully probabilistic Bayesian inference introduces a higher baseline wall-clock time compared to standard deterministic methods like PARAFAC, MANTRA exhibits highly favorable scaling properties. Thus, while applying the model to population-level atlases may eventually necessitate further algorithmic optimizations, MANTRA efficiently scales to large, complex multi-omics datasets.

Minor points:

- The white arrows in the black box of Fig. 1 are not self-explanatory and not clear. Are they needed?

The issue has been resolved.

- Fig. 6: In (A) the font size is too small and needs adaptation. In (B) the "Immune_Status" legend looks stretched and should be corrected.
Immune status legend has been fixed.

Thus, the authors have addressed my major concerns, and the revised manuscript is substantially improved in methodological rigor, benchmarking breadth, and biological validation.

While minor enhancements (e.g., scalability discussion, explicit limitations) could further strengthen the manuscript, the core scientific and technical claims are now convincing.

****

Reviewer #2's evaluation of your response to Reviewer #1's comments:

"Please find below my remaining comments regarding the point-by-point responses to Reviewer 1:

The comment on benchmarking is addressed - where the comments of reviewer 2 wrt to computational benchmarking remain. Furthermore, in Fig. 2 there is still "MUSIC" instead of new name "MANTRA" mentioned. In the caption there is a typo "aer" -> "are" (which is not on the rebuttle letter but in the ms).
The figure was fixed and the typo has been handled.
The concerns about Fig. 3 are solved.

The supplementary Fig. 2 in the rebuttal letter is the actual Supplementary Fig. S3 in the ms. This figure is not referred in the text and not described which makes the necessary comment invisible (same holds true for S6!).
All the figures have been referred in the text now. The issues have also handled.

Concerns about Fig. 4 are resolved.
For me also the concerns on model parameters are addressed.
The response wrt to Eq. 1 is rather minimalistic and should be clarified (maybe also in the text)
It has been resolved.
Argument for the lacking MAFISTO comparison is valid. Thus, the bottom line is that the main points have been addressed but that some final edits are needed."

3rd Jun 2026
Manuscript number: MSB-2025-13234RR
Title: Interpretable multi-omics integration across mixed-order tensors with MANTRA

Dear Florian,

Thank you again for sending us your revised manuscript. We are now satisfied with the modifications made and I am pleased to inform you that your paper has been accepted for publication.

Your manuscript will be processed for publication by EMBO Press. It will be copy edited and you will receive page proofs prior to publication. Please note that you will be contacted by Springer Nature Author Services to complete licensing and payment information.

You may qualify for financial assistance for your publication charges - either via a Springer Nature fully open access agreement or an EMBO initiative. Check your eligibility: https://link.springer.com/journal/44320/how-to-publish-with-us

Should you be planning a Press Release on your article, please notify the production team via this form in order to coordinate publication and release dates: https://forms.cloud.microsoft/e/eH4A8JVP6g

If you have any questions, please do not hesitate to contact the Editorial Office. Thank you for your contribution to Molecular Systems Biology.

Kind regards,
Jingyi

Jingyi Hou, PhD
Senior Editor
Molecular Systems Biology

>>> Please note that it is Molecular Systems Biology policy for the transcript of the editorial process (containing referee reports and your response letter) to be published as an online supplement to each paper. If you do NOT want this, you will need to inform the Editorial Office via email immediately. More information is available here: https://link.springer.com/partners/embo-press/editorial-policies#Peer%20review

EMBO Press Author Checklist

Corresponding Author Name: Florian Buettner	
Journal Submitted to: MSB	
Manuscript Number: MSB-2025-13234RR	

USEFUL LINKS FOR COMPLETING THIS FORM
The EMBO Journal - Author Guidelines
EMBO Reports - Author Guidelines
Molecular Systems Biology - Author Guidelines
EMBO Molecular Medicine - Author Guidelines

Reporting Checklist for Life Science Articles (updated January
This checklist is adapted from Materials Design Analysis Reporting (MDAR) Checklist for Authors. MDAR establishes a minimum set of requirements in transparent reporting in the life sciences (see Statement of Task: 10.31222/osf.io/9sm4x). Please follow the journal's guidelines in preparing your
Please note that a copy of this checklist will be published alongside your article.

Abridged guidelines for figures
1. Data
The data shown in figures should satisfy the following conditions:
- the data were obtained and processed according to the field's best practice and are presented to reflect the results of the experiments in an accurate and unbiased manner.
- ideally, figure panels should include only measurements that are directly comparable to each other and obtained with the same assay.
- plots include clearly labeled error bars for independent experiments and sample sizes. Unless justified, error bars should not be shown for technical
- if n<5, the individual data points from each experiment should be plotted. Any statistical test employed should be justified.
- Source Data should be included to report the data underlying figures according to the guidelines set out in the authorship guidelines on Data

2. Captions
Each figure caption should contain the following information, for each panel where they are relevant:
- a specification of the experimental system investigated (eg cell line, species name).
- the assay(s) and method(s) used to carry out the reported observations and measurements.
- an explicit mention of the biological and chemical entity(ies) that are being measured.
- an explicit mention of the biological and chemical entity(ies) that are altered/varied/perturbed in a controlled manner.
- the exact sample size (n) for each experimental group/condition, given as a number, not a range;
- a description of the sample collection allowing the reader to understand whether the samples represent technical or biological replicates (including how many animals, litters, cultures, etc.).
- a statement of how many times the experiment shown was independently replicated in the laboratory.
- definitions of statistical methods and measures:
  - common tests, such as t-test (please specify whether paired vs. unpaired), simple χ2 tests, Wilcoxon and Mann-Whitney tests, can be unambiguously identified by name only, but more complex techniques should be described in the methods section;
  - are tests one-sided or two-sided?
  - are there adjustments for multiple comparisons?
  - exact statistical test results, e.g., P values = x but not P values < x;
  - definition of 'center values' as median or average;
  - definition of error bars as s.d. or s.e.m.

Please complete ALL of the questions below.
Select "Not Applicable" only when the requested information is not relevant for your study.

Materials

Newly Created Materials	Information included in the manuscript?	In which section is the information available? (Reagents and Tools Table, Materials and Methods, Figures, Data Availability Section)
New materials and reagents need to be available; do any restrictions apply?	Not Applicable	

Antibodies	Information included in the manuscript?	In which section is the information available? (Reagents and Tools Table, Materials and Methods, Figures, Data Availability Section)
For **antibodies** provide the following information:<br>- Commercial antibodies: RRID (if possible) or supplier name, catalogue number and or/clone number<br>- Non-commercial: RRID or citation	Not Applicable	

DNA and RNA sequences	Information included in the manuscript?	In which section is the information available? (Reagents and Tools Table, Materials and Methods, Figures, Data Availability Section)
**Short novel DNA or RNA including primers, probes**: provide the sequences.	Not Applicable	

Cell materials	Information included in the manuscript?	In which section is the information available? (Reagents and Tools Table, Materials and Methods, Figures, Data Availability Section)
**Cell lines:** Provide species information, strain. Provide accession number in repository **OR** supplier name, catalog number, clone number, and/**OR** RRID.	Not Applicable	
**Primary cultures:** Provide species, strain, sex of origin, genetic modification status.	Not Applicable	
Report if the cell lines were recently **authenticated** (e.g., by STR profiling) and tested for mycoplasma contamination.	Not Applicable	

Experimental animals	Information included in the manuscript?	In which section is the information available? (Reagents and Tools Table, Materials and Methods, Figures, Data Availability Section)
**Laboratory animals or Model organisms:** Provide species, strain, sex, age, genetic modification status. Provide accession number in repository **OR** supplier name, catalog number, clone number, **OR** RRID.	Not Applicable	
**Animal observed in or captured from the field:** Provide species, sex, and age where possible.	Not Applicable	
Please detail **housing and husbandry conditions**.	Not Applicable	

Plants and microbes	Information included in the manuscript?	In which section is the information available? (Reagents and Tools Table, Materials and Methods, Figures, Data Availability Section)
**Plants:** provide species and strain, ecotype and cultivar where relevant, unique accession number if available, and source (including location for collected wild specimens).	Not Applicable	
**Microbes:** provide species and strain, unique accession number if available, and source.	Not Applicable	

Human research participants	Information included in the manuscript?	In which section is the information available? (Reagents and Tools Table, Materials and Methods, Figures, Data Availability Section)
If collected and within the bounds of privacy constraints report on age, sex and gender or ethnicity for all study participants.	Not Applicable	

Core facilities	Information included in the manuscript?	In which section is the information available? (Reagents and Tools Table, Materials and Methods, Figures, Data Availability Section)
If your work benefited from core facilities, was their service mentioned in the acknowledgments section?	Not Applicable	

Design

Study protocol	Information included in the manuscript?	In which section is the information available? (Reagents and Tools Table, Materials and Methods, Figures, Data Availability Section)
If study protocol has been **pre-registered, provide DOI in the manuscript**. For clinical trials, provide the trial registration number **OR** cite DOI.	Not Applicable	
Report the **clinical trial registration number** (at ClinicalTrials.gov or equivalent), where applicable.	Not Applicable	

Laboratory protocol	Information included in the manuscript?	In which section is the information available? (Reagents and Tools Table, Materials and Methods, Figures, Data Availability Section)
Provide DOI OR other citation details if **external detailed step-by-step protocols** are available.	Not Applicable	

Experimental study design and statistics	Information included in the manuscript?	In which section is the information available? (Reagents and Tools Table, Materials and Methods, Figures, Data Availability Section)
Include a statement about **sample size** estimate even if no statistical methods were used.	Not Applicable	
Were any steps taken to minimize the effects of subjective bias when allocating animals/samples to treatment (e.g. **randomization procedure**)? If yes, have they been described?	Not Applicable	
Include a statement about **blinding** even if no blinding was done.	Not Applicable	
Describe **inclusion/exclusion criteria** if samples or animals were excluded from the analysis. Were the criteria pre-established?<br><br>If sample or data points were omitted from analysis, report if this was due to attrition or intentional exclusion and provide justification.	Not Applicable	
For every figure, are **statistical tests** justified as appropriate? Do the data meet the assumptions of the tests (e.g., normal distribution)? Describe any methods used to assess it. Is there an estimate of variation within each group of data? Is the variance similar between the groups that are being statistically compared?	Not Applicable	

Sample definition and in-laboratory replication	Information included in the manuscript?	In which section is the information available? (Reagents and Tools Table, Materials and Methods, Figures, Data Availability Section)
In the figure legends: state number of times the experiment was **replicated** in laboratory.	Not Applicable	
In the figure legends: define whether data describe **technical or biological replicates**.	Not Applicable	

Ethics

Ethics	Information included in the manuscript?	In which section is the information available? (Reagents and Tools Table, Materials and Methods, Figures, Data Availability Section)
Studies involving **human participants**: State details of **authority granting ethics approval** (IRB or equivalent committee(s), provide reference number for approval.	Not Applicable	
Studies involving **human participants**: Include a statement confirming that **informed consent** was obtained from all subjects and that the experiments conformed to the principles set out in the WMA Declaration of Helsinki and the Department of Health and Human Services Belmont Report.	Not Applicable	
Studies involving **human participants:** For publication of **patient photos**, include a statement confirming that consent to publish was obtained.	Not Applicable	
Studies involving experimental **animals**: State details of **authority granting ethics approval** (IRB or equivalent committee(s), provide reference number for approval. Include a statement of compliance with ethical regulations.	Not Applicable	
Studies involving **specimen and field samples:** State if relevant **permits** obtained, provide details of authority approving study; if none were required, explain why.	Not Applicable	

Dual Use Research of Concern (DURC)	Information included in the manuscript?	In which section is the information available? (Reagents and Tools Table, Materials and Methods, Figures, Data Availability Section)
Could your study fall under dual use research restrictions? Please check biosecurity documents and list of **select agents and toxins** (CDC): https://www.selectagents.gov/sat/list.htm	Not Applicable	
If you used a select agent, is the security level of the lab appropriate and reported in the manuscript?	Not Applicable	
If a study is subject to dual use research of concern regulations, is the name of the **authority granting approval and reference number** for the regulatory approval provided in the manuscript?	Not Applicable	

Reporting

The MDAR framework recommends adoption of discipline-specific guidelines, established and endorsed through community initiatives. Journals have their own policy about requiring specific guidelines and recommendations to complement MDAR.

Adherence to community standards	Information included in the manuscript?	In which section is the information available? (Reagents and Tools Table, Materials and Methods, Figures, Data Availability Section)
State if relevant guidelines or checklists (e.g., **ICMJE, MIBBI, ARRIVE, PRISMA**) have been followed or provided.	Not Applicable	
For **tumor marker prognostic studies,** we recommend that you follow the **REMARK** reporting guidelines (see link list at top right). See author guidelines, under 'Reporting Guidelines'. Please confirm you have followed these guidelines.	Not Applicable	
For **phase II and III randomized controlled trials**, please refer to the **CONSORT** flow diagram (see link list at top right) and submit the CONSORT checklist (see link list at top right) with your submission. See author guidelines, under 'Reporting Guidelines'. Please confirm you have submitted this list.	Not Applicable	

Data Availability

Data availability	Information included in the manuscript?	In which section is the information available? (Reagents and Tools Table, Materials and Methods, Figures, Data Availability Section)
Have **primary datasets** been deposited according to the journal's guidelines (see 'Data Deposition' section) and the respective accession numbers provided in the Data Availability Section?	Not Applicable	
Were **human clinical and genomic datasets** deposited in a public access-controlled repository in accordance to ethical obligations to the patients and to the applicable consent agreement?	Not Applicable	
Are **computational models** that are central and integral to a study available without restrictions in a machine-readable form? Were the relevant accession numbers or links provided?	Yes	Data Availability Section
If publicly available data were reused, provide the respective **data citations in the reference list**.	Yes	Materials and Methods
